# Preclinical and Clinical Development of Plant-Made Virus-Like Particle Vaccine against Avian H5N1 Influenza

**DOI:** 10.1371/journal.pone.0015559

**Published:** 2010-12-22

**Authors:** Nathalie Landry, Brian J. Ward, Sonia Trépanier, Emanuele Montomoli, Michèle Dargis, Giulia Lapini, Louis-P. Vézina

**Affiliations:** 1 Medicago Inc., Québec, Canada; 2 Research Institute of the McGill University Health Center, Montreal General Hospital, Montreal, Canada; 3 Molecular Epidemiology Research Division, Department of Physiopathology, Experimental Medicine and Public Health, University of Siena, Siena, Italy; Erasmus Medical CenteThe Netherlands

## Abstract

The recent swine H1N1 influenza outbreak demonstrated that egg-based vaccine manufacturing has an Achille's heel: its inability to provide a large number of doses quickly. Using a novel manufacturing platform based on transient expression of influenza surface glycoproteins in *Nicotiana benthamiana*, we have recently demonstrated that a candidate Virus-Like Particle (VLP) vaccine can be generated within 3 weeks of release of sequence information. Herein we report that alum-adjuvanted plant-made VLPs containing the hemagglutinin (HA) protein of H5N1 influenza (A/Indonesia/5/05) can induce cross-reactive antibodies in ferrets. Even low doses of this vaccine prevented pathology and reduced viral loads following heterotypic lethal challenge. We further report on safety and immunogenicity from a Phase I clinical study of the plant-made H5 VLP vaccine in healthy adults 18–60 years of age who received 2 doses 21 days apart of 5, 10 or 20 µg of alum-adjuvanted H5 VLP vaccine or placebo (alum). The vaccine was well tolerated at all doses. Adverse events (AE) were mild-to-moderate and self-limited. Pain at the injection site was the most frequent AE, reported in 70% of vaccinated subjects versus 50% of the placebo recipients. No allergic reactions were reported and the plant-made vaccine did not significantly increase the level of naturally occurring serum antibodies to plant-specific sugar moieties. The immunogenicity of the H5 VLP vaccine was evaluated by Hemagglutination-Inhibition (HI), Single Radial Hemolysis (SRH) and MicroNeutralisation (MN). Results from these three assays were highly correlated and showed similar trends across doses. There was a clear dose-response in all measures of immunogenicity and almost 96% of those in the higher dose groups (2×10 or 20 µg) mounted detectable MN responses. Evidence of striking cross-protection in ferrets combined with a good safety profile and promising immunogenicity in humans suggest that plant-based VLP vaccines should be further evaluated for use in pre-pandemic or pandemic situations.

**Trial Registration:**

ClinicalTrials.gov NCT00984945

## Introduction

The recent swine H1N1 influenza pandemic (pH1N1) revealed the limitations of the current influenza vaccine manufacturing technologies. On May 26, 2009, the WHO recommended rapid development of vaccines and the first reassortant virus was available on May 27 2009 [1]. Although Wyman and colleagues had predicted in 2007, that egg-based manufacturing would be able to supply at least 60 million vaccine doses within 5 months of the declaration of a pandemic [2], the actual vaccine output in the 2009-10 pH1N1 pandemic was much lower. In fact, only 3 million doses of live attenuated vaccine were available at 5 months. The first doses for split vaccine became available shortly thereafter but at levels far below expectations. Fortunately, the pH1N1 strain had a low mortality rate compared to the 1918-19 pandemic strain. Had the pH1N1 pandemic had a higher severity index, the global human cost of the delays in egg-based vaccine production could have been catastrophic.

It is ironic that the world's attention had been focused on H5N1 viruses for more than a decade when pH1N1 emerged. It is important that the global sense of relief at the relatively benign nature of the pH1N1 pandemic should not lull us into complacency regarding H5N1 influenza and other potential pandemic strains. Although human-to-human spread has eluded H5N1 viruses to date, a total of 498 human cases have been reported to the WHO with a crude fatality rate of 59% (as of May 6, 2010). Continued hyper-mutation of H5N1 strains is occurring worldwide in avian populations and reassortment with an influenza strain actively circulating in humans is always a serious threat. Jackson S and colleagues have recently shown that such reassortment can readily occur between human H3N2 and avian H5N1 strains in ferrets [3]. In parallel, Li and colleagues have shown that reassortment between a low pathogenicity avian H5N1 strain and a human H3N2 strain can yield a new strain highly pathogenic for mice [4]. This latter reassortant H5N1 strain had acquired only the PB2 segment from the human strain. Regardless of the origin, mechanism of emergence or precise genetic makeup of the ‘next’ pandemic strain, our recent experience with pH1N1 demonstrate clearly that the current, egg-based manufacturing system would not be able to respond quickly enough in the face of a highly pathogenic influenza virus adapted for rapid human-to-human spread.

We have recently described a plant-based manufacturing technology that can produce vaccine doses within one month of the sequencing of a pandemic strain [5]. This is accomplished by the cloning the novel hemagglutinin gene into a well-characterized vector followed by transient expression in *Nicotiana benthamiana* plants. Five to six days after infection, plants are harvested and the Virus-Like Particle (VLP) vaccine is purified. This paper describes the efficacy of two doses of plant-made VLPs bearing the H5 from A/Indonesia/5/05 against cross-clade lethal challenge in ferrets. We also describe the first use of any plant-made VLP vaccine in humans: in this case, safety and early immunogenicity following two intramuscular (IM) doses of an H5-VLP candidate vaccine. In addition to routine safety monitoring, we also determined whether or not IgG and IgE antibodies specific for plant glycans were induced by this novel vaccine candidate.

## Methods

The protocol for this trial and supporting CONSORT checklist are available as supporting information; see Checklist S1 and Protocol S1.

### Vaccine production

The H5 VLP vaccine was produced as described in D'Aoust *et al* [6]. Briefly, whole *N. benthamiana* plants (41–44 days old) were vacuum infiltrated in batches with an *Agrobacterium* inoculum containing the H5 expression cassette. Six days after infiltration, the aerial parts of the plants were harvested and homogenized in one volume of buffer [50 mM Tris, 150 mM NaCl: 0.04% (w/v) Na_2_S_2_O_5_, pH 8.0]/kg biomass. The homogenate was pressed through a 400 µm nylon filter and the fluid was retained. The solution was brought to pH 5.3±0.1 with 5 M acetic acid and heated to 41±2°C for 15 minutes to allow aggregation of insolubles which were then pelleted at room temperature in a continuous-flow SC6 centrifuge at 1.2 L/min. The supernatant was mixed with diatomaceous earth (1% w/v), adjusted to pH 6.0±0.1 with TRIS base and passed through a 0.45/0.2 micron filter. The extract was then concentrated by tangential flow filtration (TFF) on a 500,000 Da MWCO membrane and diafiltered against 50 mM NaPO4, 500 mM NaCl and 0.005% (v/v) Tween 80 (pH 6.0). Formaldehyde was added to reach 0.0125% final concentration and the remaining insolubles removed by microfiltration.

This clarified extract was then passed through a Poros HQ column equilibrated at pH 7.5 with 50 mM Tris-HCl -0.01% Tween 80. The flow-through was captured on a Poros HS column equilibrated in 50 mM NaPO4, 0.01% Tween 80 (pH 6.0)(Applied Biosystems, USA). After washing with 50 mM NaPO4, 65 mM NaCl, 0.01% Tween 80 (pH 6.0), the VLPs were eluted with 50 mM NaPO4, 500 mM NaCl, 0.01% Tween 80 (pH 6.0) and then captured on a Poros EP 250 coupled to bovine fetuin (30 mg fetuin/mL Poros EP 250 matrix)(Desert Biologicals, Australia) as recommended by the manufacturer and equilibrated in 50 mM NaPO4, 150 mM NaCl (pH 6.0). The column was washed with 50 mM NaPO4, 400 mM NaCl, (pH 6.0) and the VLPs were eluted first with 1,5 M NaCl, and then water containing 0.0005% Tween 80. The purified VLPs were concentrated by TFF on a 300,000 Da MWCO membrane, diafiltered against formulation buffer (100 mM PO4, 150 mM NaCl, 0.01% Tween 80 at pH 7.4) and passed through a 0.22 µm filter for sterilisation.

### Vaccine characterization

Electron microscopy was performed as previously described by D'Aoust *et al* [6]. A quantitative SRID assay was performed essentially as described by *Wood et al* [7] with the following modifications. Reference antibodies and HA antigen reagents for the influenza A/Indonesia/5/2005 strain were supplied by the US FDA CBER (Kensington, MD). The pre-treatment buffer for both the H5 VLP and reference antigens included 1% Triton X-100. The SRID assay was used to estimate HA content of the H5 VLP vaccine.

SDS–PAGE analysis of VLP preparations was performed on pre-cast gels, Criterion™ XT 4–12% Bis-Tris (Bio-Rad Laboratories Hercules, CA). Samples were mixed with 4X SDS sample buffer with DTT (EMD Chemicals Inc., Gibbstown) and 2.5 µg of protein was loaded per lane. Gels were processed according to the manufacturer's instructions and stained with BioSafe™ Coomassie G-250 (Bio-Rad Laboratories Hercules, CA).

Endotoxin levels were determined by the *Limulus* amebocyte lysate test kit (QCL-1000, Lonza, Wakersville, MD) using the internal Escherichia coli 0111:B4 control.

Detection of residual DNA was performed with the PicoGreen® fluorescent dye assay (Invitrogen Canada, Burlington, ON) and measured by fluorometry using Lambda DNA for the standard curve (Invitrogen Canada, Burlington, ON).

### Ferret Vaccination and Challenge

The ferret study was approved by the Institutional Animal Care and Use Committee (IACUC) of the Southern Research Institute (SRI Birmingham, AL). Male Fitch ferrets were castrated, descented and demonstrated to be seronegative negative for representative circulating human influenza A strains prior to shipment to Southern research Institue(, 6–8 months old, 0.8–1.6 kg, Triple F Farms, Sayre, PA). The ferrets were vaccinated twice intramuscularly on days 0 and 21 with H5 VLP vaccine (0.7, 1.8, 3.7 or 11.0 µg HA formulated with alum (Alhydrogel®: 0.5 mg aluminium per 0.5 mL dose) or with placebo (PBS + alum). Eight animals per group in the 1.8 or 3.7 µg vaccine and placebo groups were challenged intranasally 45 days after the 21 day boost with a lethal dose of A/Vietnam/1203/04 H5N1 clade 1 virus (10 X Ferret Lethal Dose_50_). Animals were monitored for weight loss, temperature and loss of activity weekly during vaccination and daily during challenge. All surviving animals were euthanized 14 days post-challenge. Three challenged animals in each group were sacrificed 3 days after challenge and their lungs and nasal turbinate tissues were collected, weighted, snap frozen in liquid nitrogen until used for virus titres. Homogenized samples were serially diluted 10-fold and inoculated into viable 10 to 11 day old embryonated hen's eggs and viral titers were measured by the Egg Infectious Dose_50_ (EID_50_) assay. Data are expressed as log_10_EID_50_/mL using the Reed-Muench method [8]. Blood was collected from anesthetized ferrets via the anterior vena cava before the first and second immunization as well as 14 days after the second immunization. Sera were stored in aliquots at −20°C until use.

### Assessment of immune response

#### Hemagglutination Inhibition assay

The Hemagglutination inhibition (HI) assay was performed according to WHO recommendation [9] using whole inactivated virus for H5N1 strains: A/Indonesia/5/05 (homologous subclade 2.1 strain: CBER), A/Vietnam/1203/2004 (subclade 1: CBER), A/Anhui/01/2005 (subclade 2.2: NIBSC, Potters Bar, UK) and A/turkey/Turkey/1/05 (subclade 2.3: NIBSC). Briefly, sera were pre-treated with receptor-destroying enzyme II (RDE II) (Denka Seiken Co., Tokyo, Japan) overnight at 37°C and then PBS (Thermo Scientific, Rockford, IL) was added to create two dilutions (1∶8 and 1∶10). Sera were serially diluted 2-fold in V-bottom microtiter plates. Twenty five µL of test virus (2–8 HAU/50 µL) were added to each well and plates were incubated at room temperature for 30 min (ferret sera) or 90 min (human sera) prior addition of 0.5% horse erythrocytes (Lampire Biologicals, Pipersville, PA). Plates were incubated at room temperature for 60 to 90 min and HI titers were defined as the reciprocal of the highest dilution causing complete inhibition of hemagglutination. Seroconversion was defined as a fourfold increase in HI titer or from baseline (≤8) to HI titers ε32. Seroprotection was defined as the proportion of subjects with HI titer ≥40. For the Phase 1 clinical trial, HI titers was evaluated according to the EU EMEA CHMP criteria [10] for non-elderly adults (seroprotection >70%; seroconversion or significant increase>40%; geometric mean titers ratio GMR increases >2.5).

#### Microneutralisation Assay

Microneutralization (MN) assays were performed as previously described [11] 21 days after the booster injection, using living H5N1 virus A/Indonesia/5/05 (homologous strain of subclade 2.1, provided by the CDC, Atlanta, GA) and the living H5N1 virus A/Vietnam/1203/2004 (heterologous strain of clade 1, provided by the CDC).

#### Single Radial Hemolosyis Assay

Single radial hemolysis (SRH) was performed at the University of Siena, Italy, against whole inactivated A/Indonesia/5/2005 (H5N1) virus (subclade 2.1 strain: CBER). The SRH was modified slightly from the method described by Schild et al [12]. Briefly, 2 sets of SRH plates were prepared using 10% turkey erythrocytes (v/v of assay buffer) with 2000 Hemagglutinin units (HU)/mL of whole inactivated virus. Serum samples were heat inactivated at 56°C for 30 min, and serial 2-fold dilutions were prepared with assay buffer. Serum dilutions were added to the plates (6 µL final volumes) on duplicate plates and both pre and post-vaccination sera were titrated simultaneously. Laboratory staff was blind to group assignment. The diameters of the haemolytic areas were measured using a Transidyne Calibrating Viewer (Transidyne General Corporation, Ann Arbor, MI). Haemolysis Area <4 mm^2^ was considered negative, between 4 and 25 mm^2^ was considered positive but not protective and >25 mm^2^ was considered protective, as per EU EMEA CHMP guidelines [10]. Each test run included negative and positive controls: the latter was hyperimmune sheep serum provided by CBER.

### Antibody Response to Plant Glycans

Ninety-six-well plates were coated overnight at 4°C with 50 µL of either avidin (1 mg/mL) derived from egg white: (Sigma-Aldrich, St-Louis, MO) or recombinant avidin expressed in corn as a source of plant glycans (1 mg/L: Sigma-Aldrich) in 50 mM sodium carbonate buffer (pH 9.6). The presence of plant-specific xylose and fucose on the recombinant corn avidin was established by Western blot. Plates were washed with PBS containing 0.1% Tween-20 (PBS-T) and blocked with 1% casein in PBS-T (blocking solution) for 1 h at 37°C. The plates were then incubated with serial two-fold dilutions of sera in blocking solution for 1 h at 37°C. The plates were washed (PBS-T) and incubated for 1 h at 37°C with HRP-conjugated donkey anti-human IgG (H+L) (Jackson ImmunoResearch Inc.) or HRP-conjugated goat anti-human IgE (Sigma-Aldrich). Both conjugates were diluted at 1∶10,000 in blocking solution. HRP activity was detected by addition of 100 µl SureBlue TMB Microwell Peroxidase Substrate (KPL, Kirkegaard & Perry Laboratories, Inc.). After 20 minutes at room temperature, the enzymatic reaction was stopped with 100 µl 1N HCl and OD was determined at 450 nm. Differences pre- and post-immunization were calculated as the highest titer giving a difference >0.1 OD. Rabbit antibodies raised against xylose or fucose residues (AgriSera) were used as controls. ‘Positive’ reactions were defined as a two-fold difference between the titer for corn avidin versus egg avidin.

### Phase I clinical trial

This randomized, double-blind, placebo-controlled Phase I clinical trial was performed at the McGill University Health Centre (MUHC) Vaccine Study Center, (Pierrefonds, QC) to assess the safety and immunogenicity of the plant-produced H5 VLP vaccine (registered at ClinicalTrials.gov: NCT00984945). The trial was approved by both the Canadian Biologic and Genetic Therapies Directorate and the Research Ethics Committee of the MUHC (Final approval, document Dated of September 2^nd^ 2009.

### Subjects

Forty eight healthy adults aged 18–60 years were recruited. The principal exclusion criteria included significant medical or neuropsychiatric illness, immunosuppression or immunodeficiency; ongoing febrile illness; history of autoimmune disease; history of H5N1 vaccination; any vaccination within a 30 day period prior to enrolment, or planned vaccination between the first vaccination up to blood sampling at Day 42; use of any investigational or non-registered product within 90 days prior to study enrolment or planned use during the study; systemic glucocorticoid therapy; coagulation disorders or treatment with anticoagulants; history of allergy to constituents of H5 VLP (H5N1) vaccine or tobacco; history of severe allergic reactions or anaphylaxis; receipt of a blood transfusion or immunoglobulins within 90 days of enrolment; pregnancy; lactation; or cancer or treatment for cancer within 3 years of vaccine administration. Written informed consent was obtained from all subjects.

### Vaccine

H5 VLP vaccine was produced as described above and formulated with 1% Alhydrogel® prior to vaccination (0.5 mg aluminium per 0.5 mL dose). Groups of 12 subjects received vaccines containing 5, 10 or 20 µg of HA (as assessed by SRID assay). The placebo group received PBS buffer (100 mM phosphate, 150 mM NaCl, 0.01% Tween 80) + alum (as above).

### Procedure

Starting at the lowest dose of H5 vaccine (5 µg/dose), groups of 16 subjects were randomized using block permutation to receive either active vaccine (n = 12) or placebo (n = 4) and the study was completed in 3 waves. At each dose level, safety data for the first 7 days after vaccination was reviewed by an independent panel before the next group of 12+4 subjects was dosed. The same staggered approach was used for booster immunizations. All subjects were immunized by a nurse masked to group assignment. All immunizations were administered in the deltoid muscle and subjects were observed for at least 2 hours after each immunization for any signs or symptoms of local or systemic reaction. Vital signs were assessed hourly during this period. Serum was collected before and 21 days after each immunization and aliquots were held at 2 to 8°C for biochemistry and haematologic analyses. Additional aliquots were stored at −20°C until analyzed for immunologic assays. At the time of writing, a 6-month follow-up period is ongoing.

### Safety analysis

A memory aid, rulers and thermometers were given to all subjects to record adverse events occurring up to Day 21 after each vaccination. The occurrence of solicited local and systemic reactions was recorded daily for 7 days following each dose (See Results section for the list of solicited reactions). Severity was assessed using a grading scale of 0 to 3 and each reported adverse event (AE) was reviewed by a masked study investigator (BJW) for clinical significance and to assign a probability that each AE was vaccine-related.

### Statistical analysis

HI titers in the ferret study were assessed using the Student's *t*-test. For the Phase I clinical trial, demographic data were compared among treatment group using Fisher's exact test for categorical factors and Kruskal-Wallis test for continuous factors. AEs were coded using the Medical Dictionary for Regulatory Activities (MedDRA®), version 12.1. The incidence of solicited signs and symptoms and treatment-emergent AEs were compared using Fisher's exact tests (each vaccine dose level versus placebo). Results were summarized using point estimates and 2-sided 95% confidence interval (95% CI). For the clinical trial, the principal HI endpoints were the geometric mean titer (GMT) and geometric mean increase (GMI); seroconversion was defined as the proportion of subjects with a fourfold increase in HI titers or a HI titer ≥32 when pre-vaccination titer was <8, seroprotection was defined as the proportion of subjects with HI titers ≥40. In line with European regulatory guidance [10], the principal SRH endpoints were the geometric mean area (GMA) and the geometric mean of the increase (GMI); seroconversion was defined as the proportion of subjects with ≥50% increase in area post-vaccination or with GMA ≥25 mm^2^ when pre-vaccination SRH was negative; seroprotection was defined as the proportion of subjects with an SRH titer ≥25 mm^2^. For MN titers, as no protective threshold titer has been defined yet, endpoints were the geometric mean titer (GMT) and seroconversion was defined as the proportion of subjects with a fourfold increase in MN titer or a MN titer ≥40 when pre-vaccination titer was <10. For all three serological assays, the proportion of subjects showing an antibody response above baseline was reported. Comparisons between HI, SRH and MN results were performed based on the log10 values of the titers at Day 42 and by calculating the Spearman correlation coefficient for selected pairs.

## Results

### Vaccine production

**Figure 1:**
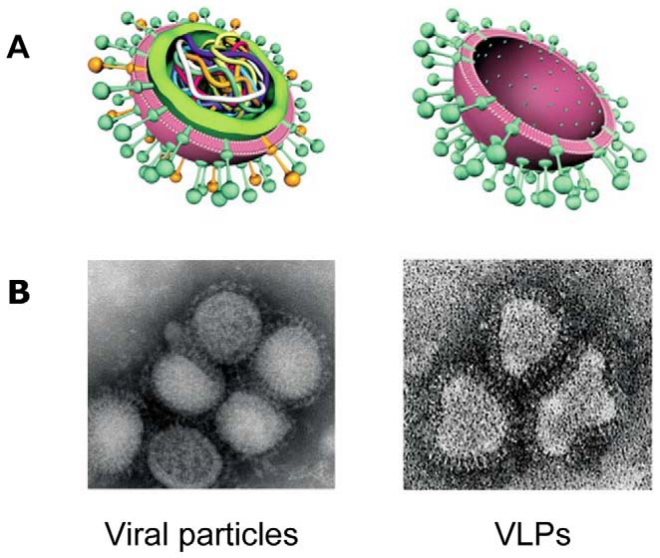
Schematic representation of the structural characteristics of viral particles and plant-made VLPs. A. Cross-section showing internal differences. B. Transmission electron microscopy images of influenza viruses and plant-made VLPs.

The H5 VLP vaccine was produced in accordance with Good Manufacturing Procedures (GMP) for Phase 1 clinical grade material. The vaccine contained the HA protein of the A/Indonesia/5/05 H5N1 anchored in plasma membrane from *N. benthamiana* cells (Figure 1). Purity assessed by Coomassie-stained gel was ε91% HA. Endotoxin and DNA content were below typical limits for an injectable biological product of this type. Vaccine dosages were adjusted based on the Single Radial ImmunoDiffusion (SRID) assay described above.

### The H5 VLP vaccine provides cross-clade protection in ferrets

The VLP vaccine induced detectable HI titers at all doses tested (0.7 to 11((g) (Table 1). Twenty-one days after the first dose, 80% and 87.5% of the ferrets in the 0.7 µg and 1.8 µg groups respectively and 100% of the animals in the 3.7 and 11.0 µg groups had developed detectable HI responses against the homologous strain (A/Indonesia/05/05: clade 1). The highest HI titer (GMT = 40, 95%CI 17–95) after the first dose was observed in the 11 µg group. Twenty-one days after the second dose, 100% of ferrets had detectable HI antibodies with GMTs ranging from 255 (95% CI 94–691) in the 0.7 µg group to 560 (95% CI 234–1324) in the 11 µg group.

**Table 1:** Serum HI titers against homologous and heterologous H5N1 strains in ferrets vaccinated with clinical grade material.

Vaccine Dose^a^	Vaccination	HI titers^d^ (95% CI) *(number of responders)*			
		Indo/5/05 (clade 2.1)	Turkey/Turkey/1/05 (clade 2.2)	Anhui/1/05 (clade 2.3)	VN/1203/04 (clade 1)
0.7 µg VLP	1^st^ dose^b^	**17** (7–45)*(4/5)*	**23** (4–130)*(4/5)*	**5** (3–8)*(1/5)*	**<10** *(0/5)*
	2^nd^ dose^c^	**255** (94–691)*(5/5)*	**32** (6–178*(4/5)*	**48** (8–307)*(4/5)*	**21** (6–78)*(4/5)*
1.8 µg VLP	1^st^ dose^b^	**23** (16–34)*(14/16)*	**14** (7–29)*(9/16)*	**6** (4–8)*(6/16)*	**5** (5–6)*(1/16)*
	2^nd^ dose^c^	**429** (204–902)*(8/8)*	**124** (61–252)*(8/8)*	**74** (26–210)*(7/8)*	**24** (9–67)*(6/8)*
3.7 µg VLP	1^st^ dose^b^	**27** (22–34)*(16/16)*	**31** (19–53)*(15/16)*	**6** (4–8)*(6/16)*	**6** (5–7)*(1/16)*
	2^nd^ dose^c^	**382** (236–619)*(8/8)*	**78** (27–229)*(7/8)*	**78** (42–145)*(8/8)*	**28** (11–71)*(7/8)*
11.0 µg VLP	1^st^ dose^b^	**40** (17–95)*(5/5)*	**19** (3–120)*(3/5)*	**5** (3–8)*(1/5)*	**<10 ** *(0/5)*
	2^nd^ dose^c^	**560** (234–1342)*(5/5)*	**106** (53–211)*(5/5)*	**97** (54–173)*(5/5)*	**42** (16–113)*(5/5)*

a All vaccines were formulated with Alhydrogel 1% (0.5 mg per dose).

b HI titers measured 21 days after vaccination.

c HI titers measured 14 days after boost vaccination.

d Geometric Mean Titer measured on all animals. HI negative animals were given an arbitrary value of 4 or 5 depending on starting dilution in the HI assay.

The H5 VLP vaccine also induced detectable cross-reactive HI antibodies in ferrets. Not surprisingly, cross-reactive HI titers were highest after the second dose (Table 1). The highest GMT was seen for a clade 2.2 strain (A/turkey/Turkey/1/05) followed by a clade 2.3 strain (A/Anhui/1/05). All ferrets of the 11 µg group had detectable HI titers for these two strains but responses were more variable at lower doses (0.7, 1.8 and 3.7 µg). None of the placebo-immunized ferrets had detectable HI antibodies for any strain (data not shown).

HI titers were overall somewhat lower for the clade 1 challenge strain (A/Vietnam/1203/04) than for clade 2 strains. Twenty-one days after the second dose, HI titers to A/Vietnam/1203/04 were detectable in only 75–87.5% of the challenged ferrets (See Table 1). The highest GMT in challenged groups (GMT = 28, 95% CI 11–71) was seen in the 3.7 µg group.

**Figure 2:**
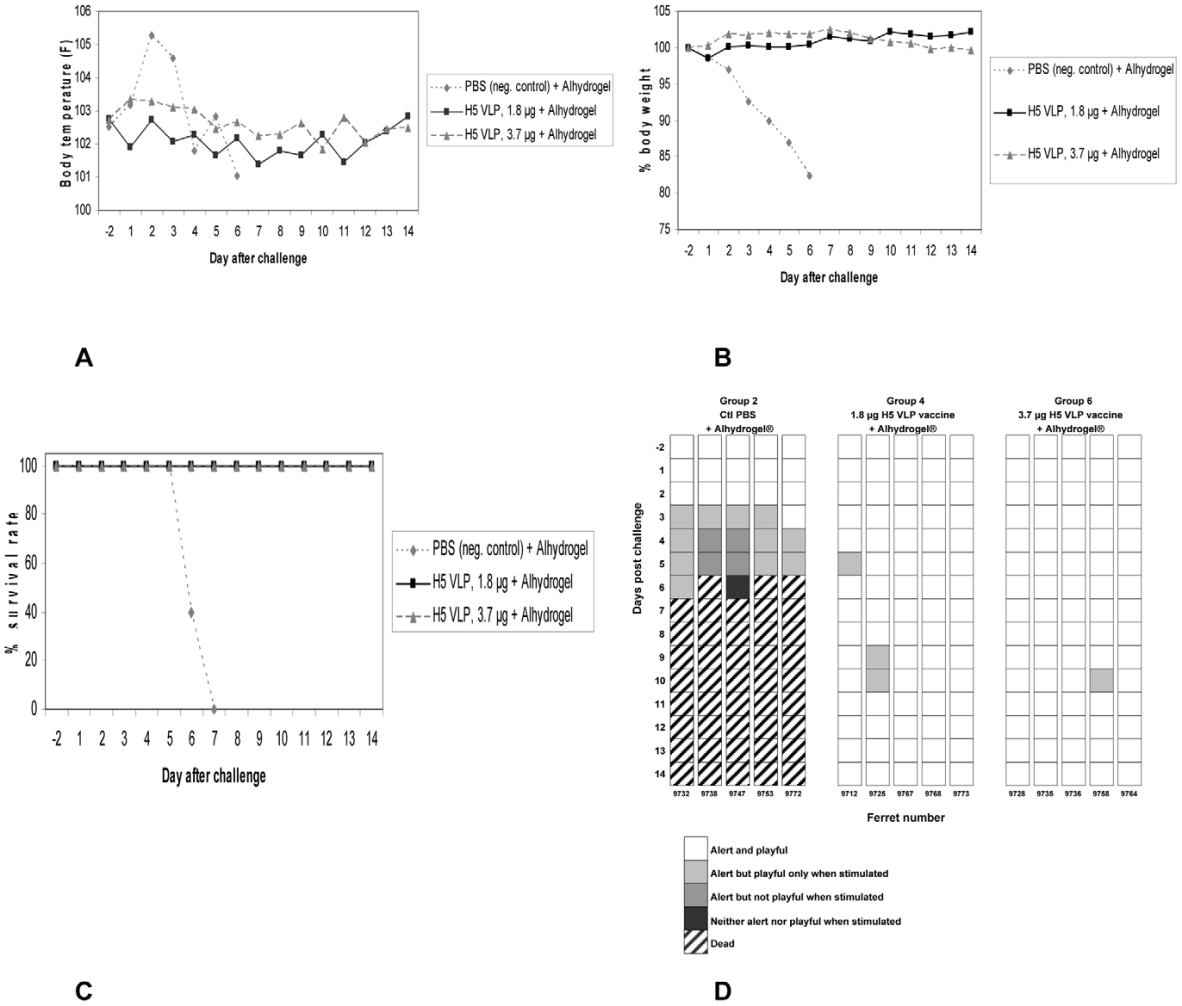
Protection against lethal challenge of control and vaccinated ferrets after challenge with A/Vietnam/1203/04 (H5N1) virus. Ferrets were immunized twice with the H5 VLP vaccine (A/Indonesia/5/05) or placebo (alum) and were challenged with 10 FLD_50_ of the A/Vietnam/1203/04 H5N1 strain 45 days after the booster injection. A. Mean temperature (5 ferrets per group) B. Percent weight loss (at day 6, 3 ferrets were found dead and the 2 remaining were euthanized due to ≥20% body weight loss C. Survival D. Activity score.

The challenge with A/Vietnam/1203/04 in the 1.8 and 3.7 µg and placebo groups revealed complete protection from clinical illness in the vaccinated animals only. In the first days after challenge, ferrets in the placebo group had a marked increase in fever followed by a decrease in body temperature while vaccinated animals had no important body temperature fluctuations (Figure 2A). Activity scores in the placebo animals also fell sharply, starting 3 days after challenge. At day 6, 3 of placebo recipients were found dead and the other two were euthanized because they had lost >20% of their body weight. All of the vaccinated ferrets survived the lethal challenge (Figure 2B and 2C) and suffered little observable morbidity (Figure 2D). There was no clear difference in fever, body weight loss or activity scores in the ferrets vaccinated with either the 1.8 or 3.7 µg dose. Upper respiratory tract (URT) and lung tissues harvested in each of the challenge groups 3 days after challenge revealed detectable virus in the URT all placebo recipients (3/3) but only 1/3 and 2/3 in the 1.8 µg and 3.7 µg groups respectively (Table 2).

**Table 2:** Responses to VLP vaccination in ferrets.

Experiment	Pre-challenge HI GMT^a^ (fraction of vaccine responders)		Mean viral load^b^ (number of positive animals)		Mean % body weight change	Survival
	Indo/5/05	VN/1203/04	URT	lungs		
After 2^nd^ dose						
1.8 µg VLP	429 (8/8)	24 (6/8)	3.5 (1/3)^c^	2.0 (2/3)	+2.1	5/5
3.7 µg VLP	382 (8/8)	28 (7/8)	2.5 (2/3)^c^	<1.5 (0/3)	−0.3	5/5
PBS	<8 (0/8)	<8 (0/8)	3.2 (3/3)	2.25 (1/3)	−17.7	0/5

a HI titers measured on sera taken 14 days after last vaccination.

b Values are expressed as log_10_EID_50_/ml, mean calculated on positive animals only. Individual values by dose and location: 1.8 µg URT (3.5, < LOD,< LOD) lungs (1.98, 2.0, <LOD); 3.7 µg URT (2.5, 2.5, < LOD) lung (< LOD, < LOD,< LOD), placebo URT (3.75, 3.33, 2.5) lungs (2.25, 2 x< LOD).

c Virus titration performed on animals sacrificed 3 days post-challenge.

### Safety and reactogenicity of the H5 VLP vaccine in humans

**Figure 3:**
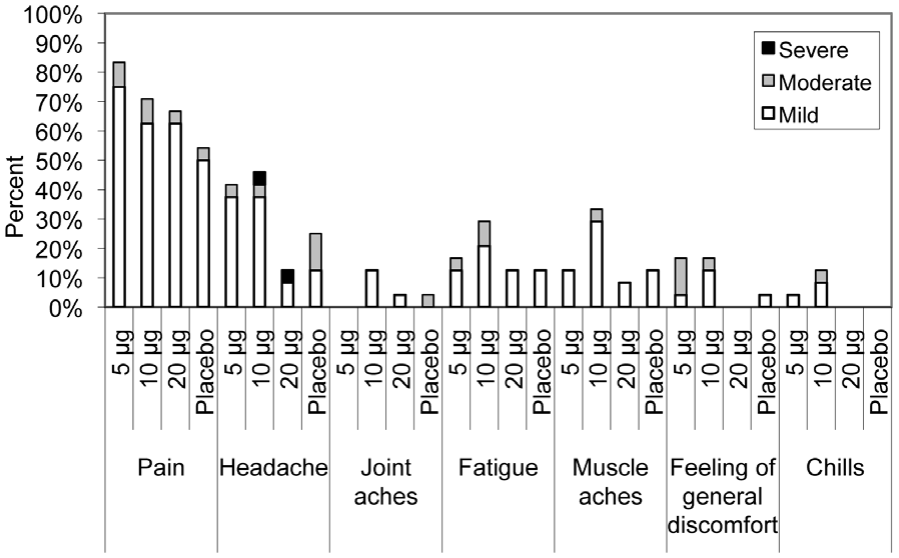
Rates and severity of local and systemic adverse events (AEs) during the first 7 days after the first and second doses. Symptoms are graded on the following scale: mild  =  subject is aware of the AE but it causes no limitation of usual activities, moderate  =  subject is aware of the AE and the event causes some limitation of usual activities and severe  =  AE is of such severity that the subject is unable to carry out usual activities.

The H5 VLP vaccine was well tolerated. Although the sample size was limited for this first-in-human study, 12 subjects per treatment group should permit the capture of AEs having 10% or 5% incidences with probabilities of ∼72% and ∼46% respectively. There were no Serious AEs reported up to Day 42 (21 days after second dose). The 6-month follow-up period is still ongoing at time of writing. Pain at injection site, redness and headache were the most commonly reported local and systemic reactions (Table 3). Fever >38°C was not reported in any subject. There were no significant differences in the incidence of either local or systemic reactions between any vaccine group and the placebo group except for redness in the 20 µg group after the first dose only (*p* = 0.04). Local and systemic reactions were mostly mild (Figure 3) and of short duration. The only reactions reported as ‘severe’ were transient headaches in 1/12 (8.3%) in the 20 µg group after first dose and in the 10 µg group after the second dose. However, there was no statistical difference in the overall incidence of headache between the vaccine and placebo recipients.

**Table 3:** Adverse events per group by treatment.

	First dose				Second dose			
Adverse event	5 µg	10 µg	20 µg	Placebo	5 µg	10 µg	20 µg	Placebo
**Local reaction**								
Redness	2 (16.7; 0.16–1.00)	4 (33.3; 0.40–1.00)	9 (75.0; 0.66–1.00)	3 (25.0; 0.29–1.00)	8 (66.7; 0.63–1.00)	2 (16.7; 0.16–1.00)	5 (41.7; 0.48–1.00)	5 (41.7; 0.48–1.00)
Swelling	1 (8.3; 0.03–1.00)	0	4 (33.3; 0.40–1.00)	2 (16.7; 0.16–1.00)	2 (16.7; 0.16–1.00)	5 (41.7; 0.48–1.00)	1 (8.3; 0.03–1.00)	3 (25.0; 0.29–1.00)
Pain	11 (91.7; 0.72–1.00)	8 (66.7; 0.63–1.00)	8 (66.7; 0.63–1.00)	7 (58.3; 0.59–1.00)	9 (75.0; 0.66–1.00)	9 (75.0; 0.66–1.00)	8 (66.7; 0.63–1.00)	6 (50.0; 0.54–1.00)
**Systemic reactions**								
Fever	0	0	0	0	0	0	0	0
Headache	4 (33.3; 0.40–1.00)	6 (50.0; 0.54–1.00)	2 (16.7; 0.16–1.00)	4 (33.3; 0.40–1.00)	6 (50.0; 0.54–1.00)	5 (41.7; 0.48–1.00)	1 (8.3; 0.03–1.00)	2 (16.7; 0.16–1.00)
Joint aches	0	1 (8.3; 0.03–1.00)	1 (8.3; 0.03–1.00)	0	0	2 (16.7; 0.16–1.00)	0	1 (8.3; 0.03–1.00)
Fatigue	0	4 (33.3; 0.40–1.00)	1 (8.3; 0.03–1.00)	1 (8.3; 0.03–1.00)	4 (33.3; 0.40–1.00)	3 (25.0; 0.29–1.00)	2 (16.7; 0.16–1.00)	2 (16.7; 0.16–1.00)
Muscle aches	2 (16.7; 0.16–1.00)	5 (41.7; 0.48–1.00)	2 (16.7; 0.16–1.00)	3 (25.0; 0.29–1.00)	1 (8.3; 0.03–1.00)	3 (25.0; 0.29–1.00)	0	0
Feeling of general discomfort	2 (16.7; 0.16–1.00)	2 (16.7; 0.16–1.00)	0	1 (8.3; 0.03–1.00)	2 (16.7; 0.16–1.00)	2 (16.7; 0.16–1.00)	0	0
Chills	0	2 (16.7; 0.16–1.00)	0	1 (8.3; 0.03–1.00)	1 (8.3; 0.03–1.00	1 (8.3; 0.03–1.00	0	0

Data are number(%; 95% CI). Adverse events up to 7 days after vaccination are reported.

### Immunogenicity of the H5 VLP vaccine in humans

**Figure 4:**
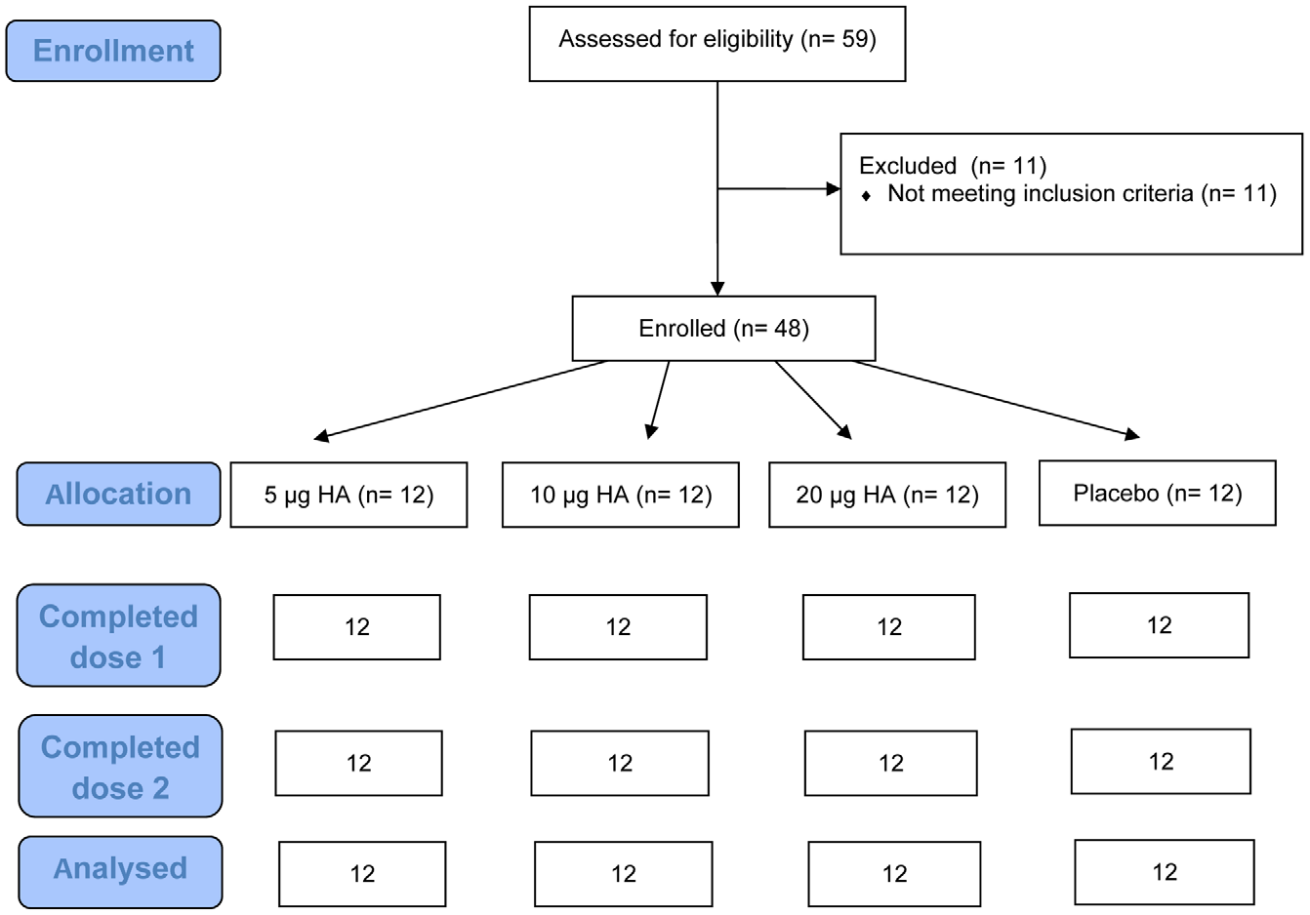
Subjects disposition. A total of 48 subjects were enrolled in the study and randomized in a 1∶1∶1∶1 ratio to receive two vaccinations of either 5, 10 or 20 µg of H5 VLP or placebo mixed with Alhydrogel. Vaccinations were administered 21 days apart.

All 48 subjects were included in the safety and immunogenicity analysis (Figure 4). Within each tested dose group, demographic characteristics were similar (Table 4). Before vaccination, 1/48 (2.1%), 5/48 (10.4%) and 0/48 (0%) of the subjects had detectable antibody titers as measured by the HI, SRH and MN assays respectively (Table 5). After a first dose, the highest GMT and GMA results were observed in the 20 µg group but differences with the placebo group were not statistically significant.

**Table 4:** Age and sex distribution in different dosing groups during phase 1 clinical trial.

	H5 VLP Vaccine			Placebo
	5 µg	10 µg	20 µg	
**Age**				
N	12	12	12	12
Mean (std. dev.)	45 (8)	42 (11)	33 (13)	40 (12)
(Min.;Max.)	(29; 56)	(21; 57)	(21; 53)	(21; 59)
**Gender N(%)**				
Male	5 (41.7%)	3 (25.0%)	7 (58.3%)	6 (50.0%)
Female	7 (58.3%)	9 (75.0%)	5 (41.7%)	6 (50.0%)

**Table 5:** Evaluation of the antibody response after first and second dose using three immunological assays.

Parameter	H5 VLP vaccine			Placebo
	5 µg HA, n = 12	10 µg HA, n = 12	20 µg HA, n = 12	n = 12
**Baseline (D0)**				
HI				
GMT	4.0	4.0	4.3 (3.7–5.1)	4.0
Number of subject with positive response (%)	0	0	8.3 (5.5–57.2)	0
Seroprotection (%)	0	0	0	0
**SRH**				
GMA	6.2 (4.4–8.8)	7.8 (5.0–12.1)	7.3 (4.6–11.4)	5.4 (4.4–6.6)
Number of subject with positive response (%)	8.3 (5.5–57.2)	16.7 (2.1–48.4)	16.7 (2.1–48.4)	0
Seroprotection (%)	0	16.7 (2.1–48.4)	16.7 (2.1–48.4)	0
**MN**				
GMT	5.0	5.0	5.0	5.0
Number of subject with positive response (%)	0	0	0	0
**3 weeks after first dose (D21)**				
**HI**				
GMT	4.7 (3.3–6.7)	5.5 (4.0–7.6)	7.5 (4.4–12.9)	4.5 (4.0–10.0)
Number of subject with positive response (%)	8.3 (0.2–38.5)	25 (5.5–57.2)	50 (21.1–78.9)	8.3 (0.2–38.5)
Seroprotection (%)	0	0	8.3 (0.2–38.5)	0
Seroconversion (%)	0	0	8.3 (0.2–38.5)	0
GMI	1.2 (0.8–1.7)	1.4 (1.0–1.9)	1.7 (1.0–3.2)	1.1 (0.9–1.3)
**SRH**				
GMA	9.7 (6.6–14.4)	10.0 (6.3–15.6)	11.2 (6.4–19.7)	6.3 (4.4–8.9)
Number of subject with positive response (%)	41.7 (15.2–72.3)	33.3 (9.9–65.1)	41.7 (15.2–72.3)	16.7 (2.1–48.4)
Seroprotection (%)	8.3 (0.2–38.5)	16.7 (2.1–48.4)	25.0 (5.5–57.2)	0
Seroconversion (%)	16.7 (2.1–48.4)	8.3 (0.2–38.5)	25.0 (5.5–57.2)	16.7 (2.1–48.4)
GMI	1.6 (1.1–2.5)	1.3 (1.1–1.6)	1.5 (0.7–3.5)	1.2 (0.9–1.6)
**MN**				
GMT	6.9 (4.5–10.6)	5.0	7.6 (5.1–11.2)	5.0
Number of subject with positive response (%)	25 (5.5–57.2)	0	33.3 (9.9–65.1)	0
Seroconversion (%)	8.3 (0.2–38.5)	0	16.7 (2.1–48.4)	0
**3 weeks after second dose (D42)**				
**HI**				
GMT	11.9 (5.6–25.5)	18.2 (9.8–33.8)	29.5 (10.7–80.9)	4.0
Number of subject with positive response (%)	66.7 (34.9–90.1)	100 (73.5–100)	75 (42.8–94.5)	0
Seroprotection (%)	16.7 (2.1–48.4)	25.0 (5.5–57.2)	50.0 (21.1–78.9)	0
Seroconversion (%)	16.7 (2.1–48.4)	25.0 (5.5–57.2)	58.3 (27.7–84.8)	0
GMI	3.0 (1.4–6.4)	4.5 (2.4–8.4)	6.8 (2.3–20.3)	1.0
**SRH**				
GMA	15.5 (9.3–26.0)	16.4 (9.4–28.7)	26.2 (13.7–50.1)	7.1 (4.4–11.4)
Number of subject with positive response (%)	50.0 (21.1–78.9)	50.0 (21.1–78.9)	75.0 (42.8–94.5)	25.0 (5.5–57.2)
Seroprotection (%)	41.7 (15.2–72.3)	41.7 (15.2–72.3)	75.0 (42.8–94.5)	8.3 (0.2–38.5)
Seroconversion (%)	41.7 (15.2–72.3)	50.0 (21.1–78.9)	58.3 (27.7–84.8)	25.0 (5.5–57.2)
GMI	2.5 (1.5–5.0)	2.1 (1.4–3.7)	3.6 (2.4–8.7)	1.3 (0.8–2.4)
**MN**				
GMT	16.8 (8.9–31.7)	28.3 (14.9–53.8)	48.1 (20.1–114.9)	5.7 (4.7–7.1)
Number of subject with positive response (%)	83.3 (51.6–97.9)	100 (73.5–100)	91.7 (61.5–99.8)	16.7 (2.1–48.4)
Seroconversion (%)	41.7 (15.2–72.3)	50.0 (21.1–78.9)	66.7 (34.9–90.1)	0

Note: Data in parenthesis are 95% CI. HI denotes Hemagglutination Inhibition assay, SRH Single Radial Hemolysis assay, MN MicroNeutralisation assay, GMT Geometric Mean Titer. GMI, Geometric Mean of the Increase.

HI titers rose to ≥40 in 16.7, 25 and 50% of the subjects after the second dose in the 5, 10 and 20 µg groups respectively (Table 5). No HI antibodies were detected in sera from subjects of the placebo group. In the vaccinated groups, the GMTs rose between the first and second doses (*p*<0.0005) and there was a clear dose response. After the second dose, HI GMTs were 11.9 (95% CI 5.6–25.5), 18.2 (95% CI, 9.8–33.9) and 29.5 (95% CI, 10.7–80.9) in the 5, 10 and 20 µg groups respectively.

GMI and seroconversion rates were calculated for the 3 serological assays used: HI, MN and SRH. The HI assay provides an estimate of IgG that can prevent agglutination of erythrocytes driven by HA of both wild and attenuated viruses. This assay has limited sensitivity for avian strains [13] but has been used extensively by regulatory agencies and has established correlates of protection for seasonal human strains. The MN assays provides an estimate of antibodies that can prevent infection of cells by live viruses. This assay is likely a more physiologic measurement of anti-viral activity than HI, but correlate of protection are not yet fully understood. The SRH assay measures antibodies that promote complement-mediated hemolysis induced by influenza antigen-antibody complexes. Results in this assay are influenced by both surface glycoproteins (HA and neuraminidase) and internal viral proteins [13].

Using HI results, the seroconversion rates after the second dose were 16.7, 25 and 58.3% for the 5, 10 and 20 µg groups respectively (Table 5). Calculated seroconversion rates were higher using both the MN (41.7, 50 and 66.7% respectively) and SRH results (41.7, 50 and 58.3% respectively). Surprisingly, the SRH assay suggested that 4/12 (25%) of the placebo group had seroconverted 21 days after the second dose (Table 5). There were no presumably ‘false’ seroconversions using the other tests (HI and MN), raising questions about the specificity of SRH assay.

Using HI results, seroprotection rates after the second dose were 16.7, 25 and 58.3% in the 5, 10 and 20 µg groups respectively. Seroprotection rates in these same groups at day 42 were higher by both MN (41.7, 50 and 66.7% respectively) and SRH (41.7, 41.7 and 75.0% respectively) (Table 5). In terms of compliance with CHMP guidelines [10], 2 out of three criteria (seroconversion >40% and GMI>2.5) were met after the second 20 µg dose using HI data, while all three criteria were met using the SRH data. (seroprotection >70%, seroconversion >40% and GMI >2.5). The MN assay consistently yielded higher titers than the HI assay (Tables 5 and 6), supporting the suggestion that the HI assay is less sensitive than the MN assay for the detection of anti-H5 antibodies [13], [14]. At 21 days after the second dose, 75.0, 75.0 and 91.7% of the subjects in the 20 µg group had detectable antibody titers as measured by the HI, SRH and MN assays respectively.

**Table 6:** Cross-reactive antibodies in human subjects at Day 42 for various H5N1 strains.

	H5N1 strains											
	A/Indonesia/5/05 (clade 2.1)			A/turkey/Turkey/1/05 (clade 2.2)			A/Anhui/1/05 (clade 2.3)			A/Vietnam/1203/04 (clade 1)		
Outcome	5 µg	10 µg	20 µg	5 µg	10 µg	20 µg	5 µg	10 µg	20 µg	5 µg	10 µg	20 µg
**HI**												
Seroconversion (%)	16.7	25.0	58.3	0	8.3	0	8.3	8.3	25.0	0	0	0
≥1∶40 (%)	16.7	25.0	50.0	0	8.3	0	8.3	0	16.7	0	0	0
GMT	11.9	18.2	29.5	4.9	6.0	7.0	6.0	7.1	8.7	4.3	4.5	4.8
**MN**												
Seroconversion (%)	25.0	25.0	58.3							0	0	8.3
≥1∶40 (%)	25.0	25.0	58.3							0	0	8.3
GMT	14.2	28.3	48.7							6.55	6.74	12.1

A good correlation was observed between the immune response measured by the HI and SRH assays (Spearman r_s_ = 0.616, *p*<0.0001) and between the SRH and MN assays (Spearman r_s_ = 0.602, *p*<0.0001). The strongest correlation was seen between the immune response measured by HI and MN assays (Spearman r_s_ = 0.795, *p*<0.0001).

Cross-reactive antibodies towards H5N1 strains both within (subclade) and across clades were detected with the HI and MN assays (Table 6). When measured by HI, cross-reactivity was highest for the clade 2.3 strain (A/Anhui//1/05) followed by clade 2.2 strain (A/turkey/Turkey/1/05). It is interesting that HI antibodies against the clade 1 challenge strain (A/Vietnam/1203/04) were barely detectable in any of the assays used (Table 6).

### Immunogenicity of plant-specific glycans in humans

The presence of glycans bearing the plant-specific α-1,3-fucose and β-1,2-xylose residues and terminal N-acetylglucosamine in the H5 VLP vaccine was confirmed by Western blot and mass spectrometry (data not shown). During the Phase I clinical trial, we sought to determine if IM administration of the vaccine induced either IgG or IgE responses to these plant-specific glycans. Although subjects with histories of severe allergies were excluded from the trial, subjects reporting mild to moderate allergies to plant derivatives (eg: seasonal allergies, hay fever, allergy to ragweed, grape or eggplant) were included. Table 7 shows the number of subjects with allergic histories who were enrolled (45.8%), including many with histories involving plant derivatives (39.5%). As shown in Table 8, subjects with allergies to plant derivatives were found in all groups but the highest incidence was in the placebo recipients (58.3%). Before vaccination, a total of 7 subjects across all groups had detectable IgG titers to plant glycans. Among these seven, only two reported mild allergies to plant derivatives. After two doses of H5 VLP vaccine, only one subject with a history of allergies (1/12) and four subjects without a history of allergies (4/24) had small but detectable increases in serum IgG specific for plant glycans. One non-allergic subject in the placebo group (1/12) also had an increase in IgG to plant glycans. No subject in the study, with or without a history of allergies, had a detectable IgE response to plant glycans either before or after vaccination. No subject reported allergic symptoms in the 21 days following each dose.

**Table 7:** Allergies reported by subjects.

Group	At screening, number of subjects reporting ongoing allergy (number;%)	
	Any known allergy	Allergy to any plant derivative (1)
5 µg H5 VLP	5 (41.7%)	5 (41.7%)
10 µg H5 VLP	5 (41.7%)	3 (25.0%)
20 µg H5 VLP	4 (33.3%)	4 (33.3%)
Placebo	8 (66.6%)	7 (58.3%)
Total in vaccine groups	14 (38.8%)	12 (33.3%)
Total in all groups	22 (45.8%)	19 (39.5%)

(1) as expressed by seasonal allergies, hay fever, allergy to ragweed or allergy to grapes or eggplant.

**Table 8:** Antibodies to plant-specific sugar moieties.

Group	Before immunisation (D0)		After two immunisations (D42)			
	Number of subjects with detectable IgGs to plant-specific sugar moieties		Number of subject with an increase in IgGs to plant-specific sugar moieties		Number of subject with an increase in IgEs to plant-specific sugar moieties	
	Total subjects	In subjects who reported known allergy to plant component	Total subjects	In subjects who reported known allergy to plant component	Total subjects	In subjects who reported known allergy to plant component
5 µg H5 VLP	1/12	1/5	2/12	1/5^a^	0/12	0/5
10 µg H5 VLP	3/12	1/3	1/12	0/3	0/12	0/3
20 µg H5 VLP	2/12	0/4	2/12	0/4	0/12	0/4
Placebo	1/12	0/7	1/12	0/7	0/12	0/7
Total	7/48 (14,6%)	2/19 (10,5%)	6/48 (12,5%)	1/19 (5,2%)	0/48 (0%)	0/19 (0%)

a Not the same subject who had detectable Abs to plant-specific sugar moieties before immunisation.

## Discussion

The 2009-10 H1N1 pandemic demonstrated that traditional egg-based manufacturing technologies are not currently in a position to provide the volume of vaccines required to respond to a global pandemic strain in a timely fashion. In fact, it is unlikely that egg-based production will ever be able to respond rapidly enough to influence the first wave of a rapidly spreading pandemic virus. Our recently described candidate H5 VLP vaccine produced in plants [5] is a potentially powerful new tool to address these shortcomings. In our initial report, we demonstrated that transient expression of the HA protein from H5/Indonesia/5/05 in *N. benthamiania* could generate large numbers of H5-bearing VLPs that were highly immunogenic in mice. Administered IM, these H5 VLPs provided excellent protection from challenge [6]. In a second report, we demonstrated that the first doses of a plant-made VLP candidate vaccine can be produced within 3 weeks of the identification of a new pandemic strain [5]. Since no genetic adaptation of the virus is needed and only the HA sequence from the new strain is required to initiate vaccine production, this plant-based platform is an interesting alternative to current technologies to increase global vaccine manufacturing capacity. In the current work, we present data from *in vitro* neutralization studies and *in vivo* challenge in ferrets that demonstrate cross-clade protection induced by our candidate vaccine. We also present safety and early immunogenicity data from a Phase I, dose-escalation trial in humans. Significantly, this is the first ever report of administration of a plant-made VLP vaccine to humans.

Safety is a critical issue for vaccines since public confidence can make or break immunization programs [15]. There have been no safety signals in the preclinical studies of our candidate H5 VLP vaccine (mice, rats, ferrets) and there were no statistical differences in the incidence or severity of local and systemic reactions between the vaccine and placebo recipients in the Phase I trial reported herein. When symptoms occurred, they were typically mild and of short duration. Since this was a first-in-human study and as the platform produces glycoproteins with plant-specific N-glycans particular attention was given to the development of hypersensitivity reactions and to the development of antibodies against plant glyco-epitopes. i.e. carbohydrate determinants with core β(1,2)- xylose and α(1,3)- fucose residues. This phase I clinical trial specifically excluded subjects with background of severe allergic reactions but allowed subjects with known mild to moderate allergies to plant derivatives. There were no new hypersensitivity reactions observed during the trial or convincing evidence of the induction of antibodies directed against plant N-glycans. Naturally-occurring IgGs to plant-specific carbohydrate determinants have been reported to occur in 25-50% of healthy human subjects [16]. This observation is consistent with our finding that 7/48 subjects (14.6%) had detectable IgGs to plant N-glycans before vaccination. Only 2 of these seven subjects with measurable antibodies to plant glycans (4.2%) were among the 19 subjects (39.5%) who declared one or more allergies to plant derivatives. These data demonstrate that IgGs to plant N-glycans can be found in subjects who do not suffer from allergies.

None of the two subjects who had both known allergies to plant derivatives and detectable levels of IgGs to plant-specific N-glycans before vaccination had increases in this type of IgGs, but one with known allergies in the VLP group showed an increase, as did 4 other subjects which had no known allergies to plant derivatives. Thus 13,9% of the VLP subjects showed a detectable increase while 8,3% of the placebo subjects showed a similar increase, and this difference has no statistical significance (*p* = 1.0). IgEs to plant-specific glycans could not be found in any of the 48 subjects. In light of all these results, it was concluded that in the context of this study, immunization with the plant-made HA VLPs did not trigger a response to plant-specific carbohydrate determinants, and known allergies to plant derivatives and/or presence of detectable levels of IgGs to plant-specific glycans did not create a predisposition to an immune response to the N-glycans present on the HA protein of the VLP vaccine. Although the number of subjects in this Phase I preclude a definitive conclusion with regards to the allergy risk from plant-produced vaccines, our data are reassuring for the continued development of these vaccines. The lack of both allergic reactions and increases in IgE following vaccination may be explained by the fact that the H5 glycans present in this VLP vaccine bear terminal N-acetylglucosamine (GlcNAc). which could conceivably have shielded the more proximal β(1,2)- xylose and α(1,3)-linked fucose residues, thereby preventing the cross-linking of IgEs and degranulation of mast cells and basophils as reported by Altmann [17].

Prior to the Phase I trial, ferret studies of the H5 VLP vaccine demonstrated the induction of good levels of neutralizing antibodies both within- and across clades as well as complete protection from cross-clade lethal challenge. It is interesting that both clinical and virologic data in the ferret study revealed excellent protection from the A/Vietnam challenge despite relatively low HI antibody titres (Table 1). This observation confirms that we do not yet fully understand the correlates of protection for influenza virus infection [18]. Although preliminary data suggest that the plant-made H5 VLPs have powerful effects on the human innate immune system (data not shown), it is not yet known if this vaccine stimulates only antibody production or both humoral and cellular arms of the immune system in either ferrets or in humans. Studies of both innate and adaptive cell-mediated responses induced by the H5 VLP vaccine are currently underway, as suggested in the CHMP guideline for licensing of pandemic vaccines [10].

As a secondary outcome, the Phase I trial provided a ‘first look’ at the immunogenicity of the H5 VLP vaccine at doses of 5, 10 and 20 µg adjuvanted with alum. Our decision to use an alum-adjuvanted formulation in the Phase I study was based, in part, on lower antibody responses in ferrets exposed to the candidate VLP vaccine without alum (data not shown). Furthermore, several human trials of egg-based split and whole inactivated virion H5N1 vaccines had suggested modest benefits of aluminium adjuvants [11], [19], [20], [21], [22]. It is noteworthy that our candidate H5 VLP vaccine induced potent antibody responses in mice in the absence of alum [6]. Future studies in ferrets as well as our planned Phase II studies in humans will include groups without alum.

Immunogenicity criteria for licensing candidate influenza vaccines vary slightly between jurisdictions but are largely based on serologic responses measured by two assays, Hemagglutination Inhibition (HI) and Single Radial Hemolysis (SRH). Although the HI assay has long been the standard serologic test for influenza, it may be less sensitive for avian strains such as H5N1 [13], [14]. The SRH assay is more sensitive for H5N1 strains [23], [24] but may also detect antibodies to internal proteins and therefore overestimate the humoral response to surface glycoproteins [13]. Because of the inherent limitations of these assays, vaccine developers increasingly rely on the MN as a more functional measure of immunogenicity [24], [25]. Finally, most of the reagents available for all of these assays have been developed and standardized for vaccines produced in eggs and may, as a result, be biased to recognize antibodies against antigens from influenza viruses grown in eggs. In light of these limitations and uncertainties, we used all three serological assays to evaluate the humoral response induced by the plant-made H5 VLP vaccine in humans. Although premature in some respects, it may be useful to discuss these data in the context of the current CHMP criteria for licensure of pandemic vaccines [10]

Using this approach, the 12 subjects in the 20 µg group met 2/3 of the CHMP criteria based on HI testing (seroconversion >40%, GMI >2.5) and 3/3 based on the SRH assay (seroprotection >70%, seroconversion >40%, GMI >2.5). As noted above however, the SRH assay also detected higher rates of apparent seroconversion and seroprotection in the placebo group than the HI test (Table 5). Overall, the results of the three assays were highly correlated although HI results were always lower than those obtained with MN or SRH. The known lack of sensitivity of the HI test for anti-H5 antibodies [23], [24] is the most likely explanation for these differences. It is harder to explain the observations that 2/12 of the subjects in the 20 µg group had seroprotective SRH titers before vaccination and 2/12 subjects in the placebo group had a 150% increase in SRH area during the 42 days study period (Table 5). Given that the SRH assay will also detect antibodies directed against non-HA antigens (eg: NA and internal proteins) it is possible that these paradoxical SRH results were the result of exposure to circulating H1N1 influenza viruses with high homology for the neuraminidase protein (or other internal proteins). This hypothesis is supported to some extent by the observation that the placebo group also experienced small increases in apparent antibody titers using the MN assay, which can also detect antibodies to a broader range of targets than just HA. Irrespective of the CHMP criteria, it is interesting that 87.5% of the subjects who received two doses of either the 10 or 20 µg VLP vaccine mounted a detectable HI response and 95.8% mounted detectable MN responses. These data are particularly encouraging in light of the fact that traditional, egg-based split H5N1 vaccines showed only modest immunogenicity in humans at doses as high as 45 µg with or without alum as an adjuvant [11], [20]. Furthermore, a recent meta analysis examining the relationship between HI titer and clinical protection against influenza including data from 5899 adult subjects and 1304 influenza cases suggested that the 50% protection threshold traditionally associated with HI titers of 1/40 [25] might be better set at 1/20 [26]. Such a shift in the protective threshold would also suggest that the 50% protection threshold was achieved by the 20 µg dose group in our Phase I.

Overall, these results are reassuring for the continued clinical development of this candidate H5 VLP vaccine. Although evidence is accumulating that newer adjuvants can significantly boost H5N1 responses when combined with split-virus antigens [27]–[28], experience with the pH1N1 pandemic revealed serious limitations for egg-based antigen production in terms of both speed and volume. Our plant-produced H5 VLP vaccine has the potential to respond to both of these deficiencies. Even at the current estimate of 20 µg per dose, the surge capacity, speed of response and simplicity of the plant-based technology, combined with the good safety profile shown in this Phase I study suggest that plant-made VLP vaccines should be further evaluated for use in pre-pandemic and pandemic situations.

## Supporting Information

Checklist S1 (DOCX)

Protocol S1 (DOCX)
