# Supplementary material for: Preclinical and Clinical Development of Plant-Made Virus-Like Particle Vaccine against Avian H5N1 Influenza
Source: PLoS One. 2010 Dec 22;5(12):e15559. doi: 10.1371/journal.pone.0015559 (PMC3008737; doi:10.1371/journal.pone.0015559)
Supplement: Protocol S1 — (DOCX) [file pone.0015559.s002.docx]

# Protocol Title

“A Phase 1 single centre, double-blind, randomized, placebo-controlled, dose-escalation study of plant-based H5 VLP (virus-like particles), (H5N1) pandemic influenza vaccine adjuvanted with Alhydrogel® (aluminium hydroxide) and administered to healthy adults 18-60 years of age“

**Protocol NUMBER:** CP-H5VLP-001

**Investigational PRODUCT:** H5 VLP (H5N1) Pandemic Influenza Vaccine

**DEVELOPMENT PHASE:**  Phase 1

**SPONSOR:** Medicago R&D Inc.

1020, route de l'Église, bureau 600

Sainte-Foy, Québec

Canada G1V 3V9

Tel. (418) 658-9393

Fax (418) 658-6699

**PRINCIPAL COORDINATING**

**INVESTIGATOR:** Dr. Brian Ward – MUHC Vaccine Study Centre

14770 Pierrefonds, Suite 204

Pierrefonds, Québec H9H 4Y6

**SPONSOR RESPONSIBLE**

**MEDICAL OFFICER:** Dr. Scott Halperin

Canadian Centre for Vaccinology

Dalhousie University – IWK Health Centre

5850/5980 University Avenue

P.O. Box 9700

Halifax, Nova Scotia B3K 6R8

**CRO (Data Mgt):** PharmaNet Specialized Pharmaceutical Services

415 McFarlan Road, Suite 201

Kennett Square, PA 19348

**CRO (Statistics and Medical Writing):** Anapharm Inc.

2500, Einstein

Québec (Québec) G1P 0A2

**CRO (Clinical Monitoring):** AC BioResearch Inc.

389 Woodcroft

Hudson, Québec J0P 1H0

**PROTOCOL DATE AND VERSION:** 22 July 09 – Version: 1.0

**Information in this protocol is confidential and should not be disclosed, other than to those directly involved in the execution or the ethical review of the study, without written authorization from Medicago Inc. (hereinafter known as “Medicago”), and its affiliates**

**TABLE OF CONTENTS**

[Protocol Title 1](#_Toc236045324)

[List of Tables 6](#_Toc236045325)

[Protocol Agreement 7](#_Toc236045326)

[Protocol Signature Page 8](#_Toc236045327)

[Contact List 9](#_Toc236045328)

[Synopsis 10](#_Toc236045329)

[List of Abbreviations 18](#_Toc236045330)

[1.0 INTRODUCTION 20](#_Toc236045331)

[1.1 Background 20](#_Toc236045332)

[1.2 Background of the Investigational Product 21](#_Toc236045333)

[1.3 Pre-Clinical Studies 23](#_Toc236045334)

[1.3.1 Ferret Challenge Study 26](#_Toc236045335)

[1.4 Rationale 28](#_Toc236045336)

[2.0 TRIAL OBJECTIVES 29](#_Toc236045337)

[2.1 Primary Objective 29](#_Toc236045338)

[2.1.1 Primary Endpoints 30](#_Toc236045339)

[2.1.2 Summary of Statistical Methodology for the Primary Objective 30](#_Toc236045340)

[2.2 Secondary Objective 30](#_Toc236045341)

[2.3 Exploratory endpoints 31](#_Toc236045342)

[3.0 INVESTIGATOR/S AND TRIAL ORGANIZATION 31](#_Toc236045343)

[4.0 INDEPENDENT ETHICS COMMITTEE (IEC) / INSTITUTIONAL REVIEW BOARD (IRB) 33](#_Toc236045344)

[5.0 INVESTIGATIONAL PLAN 33](#_Toc236045345)

[5.1 Description of the Overall Trial Design and Plan 33](#_Toc236045346)

[5.1.1 Trial Design 34](#_Toc236045347)

[5.1.2 Trial Design and Plan 35](#_Toc236045348)

[5.1.3 Inclusion Criteria 36](#_Toc236045349)

[5.1.4 Exclusion Criteria 36](#_Toc236045350)

[5.1.5 Study Visit Procedures 38](#_Toc236045351)

[5.1.5.1 Screening Procedures (up to 30 days prior to study vaccination) 38](#_Toc236045352)

[5.1.5.2 Dosing Visit (Day 0) 39](#_Toc236045353)

[5.1.5.3 Minimum 2-hour Post-Dose Observation Period 39](#_Toc236045354)

[5.1.5.4 Days 1 and 8 Telephone Contact (acceptable interval +/- 1 day), following vaccination 1 and 2) 40](#_Toc236045355)

[5.1.5.5 Day 21 Visit, second vaccination, (acceptable interval +/- 2 days) and 41](#_Toc236045356)

[Day 42, (acceptable interval +5 days) 41](#_Toc236045357)

[5.1.5.6 Final Visit - Day 228 (Acceptable interval is +14days) 42](#_Toc236045358)

[5.2 Discussion of the Trial Design 42](#_Toc236045359)

[5.3 Selection of the Trial Population 42](#_Toc236045360)

[5.3.1 Recruitment Procedures 42](#_Toc236045361)

[5.3.2 Participant Information and Consent 42](#_Toc236045362)

[5.3.5 Temporary Contraindications 43](#_Toc236045363)

[5.3.6 Removal of Participants from Treatment or Assessment 43](#_Toc236045364)

[5.3.6.1 Conditions for Withdrawal 43](#_Toc236045365)

[5.3.6.2 Lost to Follow-up Procedures 44](#_Toc236045366)

[5.3.6.3 Screen Failures 44](#_Toc236045367)

[5.3.6.4 Follow-up of Discontinuations 44](#_Toc236045368)

[5.3.7 Medical History 44](#_Toc236045369)

[5.4 Modification of the Trial and Protocol 44](#_Toc236045370)

[5.5 Interruption of the Trial 44](#_Toc236045371)

[6.0 TREATMENTS 45](#_Toc236045372)

[6.1 Vaccines Administered 45](#_Toc236045373)

[6.2 Identity of Investigational Product 45](#_Toc236045374)

[6.2.1 Composition 45](#_Toc236045375)

[6.2.2 Preparation and Administration 45](#_Toc236045376)

[6.2.3 Precautions for Use 47](#_Toc236045377)

[6.2.4 Dose Selection and Timing 47](#_Toc236045378)

[6.3 Identity of the Control Product 47](#_Toc236045379)

[6.3.1 Composition 48](#_Toc236045380)

[6.3.2 Preparation and Administration 48](#_Toc236045381)

[6.3.3 Precautions for use 48](#_Toc236045382)

[6.4 Identity of Other Products 48](#_Toc236045383)

[6.5 Product Logistics 48](#_Toc236045384)

[6.5.1 Labelling and Packaging 48](#_Toc236045385)

[6.5.2 Storage and Shipment Conditions 48](#_Toc236045386)

[6.5.3 Product Accountability 49](#_Toc236045387)

[6.5.4 Replacement Doses 50](#_Toc236045388)

[6.5.5 Return of Unused Products 50](#_Toc236045389)

[6.6 Randomization/Allocation Procedures 50](#_Toc236045390)

[6.7 Blinding and Code Breaking Procedures 50](#_Toc236045391)

[6.8 Concomitant Therapy 51](#_Toc236045392)

[7.0 SPECIMENS AND CLINICAL SUPPLIES 51](#_Toc236045393)

[7.1 Management of Samples 51](#_Toc236045394)

[7.1.1 Sample Collection 51](#_Toc236045395)

[7.1.2 Sample Preparation 52](#_Toc236045396)

[7.1.3 Sample Storage and Shipment 52](#_Toc236045397)

[7.2 Clinical Supplies 52](#_Toc236045398)

[8.0 ASSESSMENTS METHODS AND ENDPOINTS 53](#_Toc236045399)

[8.1 Safety Endpoints, Immunogenicity Endpoints, and Assessments Methods 53](#_Toc236045400)

[8.1.1 Endpoints 53](#_Toc236045401)

[8.1.1.1 Primary Endpoints 53](#_Toc236045402)

[8.1.1.2 Secondary Endpoints 53](#_Toc236045403)

[8.1.1.3 Exploratory Endpoints 54](#_Toc236045404)

[8.1.2 Assessment Methods 54](#_Toc236045405)

[8.1.3 Handling of Missing Data and Outliers 54](#_Toc236045406)

[8.2 Efficacy Endpoints and Assessment Methods 54](#_Toc236045407)

[9.0 SERIOUS ADVERSE EVENTS 55](#_Toc236045408)

[9.1 Reporting of Serious Adverse Events 55](#_Toc236045409)

[9.1.1 Adverse Events 55](#_Toc236045410)

[9.1.2 Expectedness of an Adverse Drug Reaction 56](#_Toc236045411)

[9.1.3 Initial SAE Reporting by the Investigator 56](#_Toc236045412)

[9. 1.4 Follow-up Reporting by the Investigator 57](#_Toc236045413)

[9.1.5 Reporting of SAEs Occurring after Subject Trial Termination 57](#_Toc236045414)

[9.1.6 Causal Relationship 57](#_Toc236045415)

[9.1.7 Reporting of SAEs to Health authorities and IEC/IRBs 58](#_Toc236045416)

[9.1.8 Seven (7) - Day Safety Monitoring 58](#_Toc236045417)

[10.0 DATA COLLECTION AND MANAGEMENT 59](#_Toc236045418)

[10.1 Data Collection, CRF Completion 59](#_Toc236045419)

[10.2 Data Management 60](#_Toc236045420)

[11.0 STATISTICAL METHODS AND DETERMINATION OF SAMPLE SIZE 60](#_Toc236045421)

[11.1 Statistical Methods 60](#_Toc236045422)

[11.1.1 Hypotheses and Statistical Methods for Primary Endpoints 60](#_Toc236045423)

[11.1.1.1 Hypotheses 60](#_Toc236045424)

[11.1.2 Statistical Analysis 60](#_Toc236045425)

[11.1.2.1 Immunogencity 60](#_Toc236045426)

[11.1.2.2 Safety 61](#_Toc236045427)

[11.1.3 Hypotheses and Statistical Methods for Exploratory Endpoints 61](#_Toc236045428)

[(Refer to Statistical Analysis Plan) 61](#_Toc236045429)

[11.2 Population to be Analyzed 61](#_Toc236045430)

[11.2.1 Definition of Population 61](#_Toc236045431)

[11.2.1.1 Intent-to-Treat Safety Analysis Set 61](#_Toc236045432)

[11.2.1.2 Full Analysis (Intent-to-Treat) Immunogenicity Population 61](#_Toc236045433)

[11.2.1.3 Per-Protocol Immunogenicity Population 61](#_Toc236045434)

[11.2.2 Populations Used in Analysis 61](#_Toc236045435)

[11.3 Determination of Sample Size and Power Calculation 61](#_Toc236045436)

[12.0 ETHICAL AND LEGAL ISSUES AND INVESTIGATOR / SPONSOR RESPONSIBILITIES 62](#_Toc236045437)

[12.1 Ethical Conduct of the Trial / Good Clinical Practice 62](#_Toc236045438)

[12.2 Source Documents and Source Data 62](#_Toc236045439)

[12.3 Confidentiality of Data and Access to Subject Records 63](#_Toc236045440)

[12.4 Monitoring, Auditing and Archiving 63](#_Toc236045441)

[12.4.1 Monitoring 63](#_Toc236045442)

[12.4.2 Audits and Inspections 63](#_Toc236045443)

[12.4.3 Archiving 64](#_Toc236045444)

[12.5 Financial Contract and Insurance Coverage 64](#_Toc236045445)

[12.6 Stipends for Participation 64](#_Toc236045446)

[13.0 REFERENCES 64](#_Toc236045447)

List of Tables

Table 1 Study Visit Procedures

Table 2 Pre-clinical Studies Conducted with Research Grade Material

Table 3 Serum HI titers against homologous and heterologous H5N1 strains in ferrets vaccinated with clinical grade material

Table 4 Study Design

**List of Figures**

Figure 1 Protection against lethal challenge of control and vaccinated ferrets after challenge with A/Vietnam/1203/04 (H5N1) virus.

**Appendices**

Appendix A Sample Memory Aid – Day 0 – Day 7

Sample Memory Aid - Day 8 -21 and Day 29 – 42

Appendix B Sample Telephone Follow-up Script

Appendix C Sample Seven (7) Day Safety Narrative

# Protocol Agreement

I have read this Medicago protocol no. CP-H5VLP-001

**Title:** A Phase 1 single centre, double-blind, randomized, placebo-controlled, dose-escalation study of plant-based H5 VLP (virus-like particles), (H5N1) pandemic influenza vaccine adjuvanted with Alhydrogel® and administered to healthy adults 18-60 years of age

**I have fully discussed the objectives of this trial and the contents of this protocol with the Sponsor’s representative.**

I understand that the information in this protocol is confidential and should not be disclosed, other than to those directly involved in the execution or the ethical review of the study, without written authorization from Medicago R&D Inc. It is, however, permissible to provide information to a subject in order to obtain consent once IRB/IEC approval is obtained.

I agree to conduct this trial according to this protocol and to comply with its requirements, subject to ethical and safety considerations and guidelines, and to conduct the trial in accordance with ICH guidelines on GCP and applicable regulatory requirements.

I understand that the Sponsor may decide to suspend or prematurely terminate the trial at any time for whatever reason and that such a decision will be communicated to me in writing. Conversely, should I decide to withdraw from execution of the trial I will communicate my intention immediately in writing to the Sponsor.

**Investigator Name and Address**

Brian Ward, M.D.

MUHC Vaccine Study Centre

14770 Pierrefonds Blvd, Suite 204

Pierrefonds, Québec H9H 4Y6

**Signature Date**

**__________________________________________________________________________**

**Signature Date**

**Sponsor Representative**

# Protocol Signature Page

**Protocol Title:**

A Phase 1 single centre, double-blind, randomized, placebo-controlled, dose-escalation study of plant-based H5 VLP (virus-like particles), (H5N1) pandemic influenza vaccine adjuvanted with Alhydrogel® and administered to healthy adults 18-60 years of age

**PROTOCOL NUMBER:** CP-H5VLP-001

**PROTOCOL DATE: 22 July 2009**

**SIGNATURES:**

**Protocol Author:**

**Signature Date**

**Protocol Reviewed by:**

**Signature, (CRO - Statistics) Date**

**Protocol Reviewed by:**

**Sponsor Responsible Medical Officer Date**

**Protocol Authorized by:**

**Vice-president, Product Development Date**

**Acting Vice-president, QA and Regulatory Affairs Date**

# Contact List

**Conduct of the Study**

For questions regarding the conduct of this study please contact:

**Project Leader:** Sonia Trepanier, Ph.D.

MEDICAGO R & D. INC.

1020, route de l’Eglise, bureau 600

Sainte-Foy, Québec G1V 3V9

Tel: (418) 658-9393 Ext. 137

Fax: (418) 658-6699

Mobile: (418) 655-7158

E-mail: [trepaniers@medicago.com](mailto:trepaniers@medicago.com)

**Study Monitor:** Gail Clements, M.Sc., B.N. C.C.R.A.

AC BioResearch Inc.

389 Woodcroft

Hudson, Québec J0P 1H0

E-mail:[gail.clements@sympatico.ca](mailto:gail.clements@sympatico.ca)

Tel: (450) 458-2726

Cell: (514) 910-9562

Fax: (450) 458-1761

In case of a Serious Adverse Event (SAE), the Investigator will be able to contact the study Medical Monitor at all times at one of the following numbers:

Scott Halperin, M.D.,

Tel: (902) 470-8502

**After hours:** Mobile: (902) 449-8502 or Pager: (902) 458-0492

# Synopsis

| Company: | | Medicago R & D Inc. | |
| --- | --- | --- | --- |
| Investigational Product/ Drug Product: | | Plant-based H5 VLP (Virus-like particles), (H5N1) pandemic influenza vaccine | |
| Active Substance(s): | | Recombinant H5 protein (hemagglutinin) assembling into VLPs | |
| Title of the Trial: | | A Phase 1 single centre, partially double-blind, randomized, placebo-controlled, dose-escalation study of plant-based H5 VLP (virus-like particles), (H5N1) pandemic influenza vaccine adjuvanted with Alhydrogel® (aluminium hydroxide) and administered to healthy adults 18-60 years of age | |
| Development Phase: | | Phase 1 | |
| Principal/Coordinating Investigator: | | Brian Ward, M.D. | |
| Investigators and Trial Centers: | | MUHC Vaccine Study Centre | |
| Expected Start Date | | September, 2009 | |
| Planned Trial Period: | | Forty-two (42) days – vaccinations, safety assessment and serology sampling. Six (6) months, Day 228, safety assessment and serology sampling | |
| Duration of Treatment: | | An intra-muscular injection on Day 0 and Day 21 post-vaccination | |
| Expected Completion Date: | | November, 2009, vaccinations completed. Final safety visit expected March, 2010 | |
| Primary Objective: | | The primary objective is to assess the safety and tolerability of two consecutive doses of plant-based H5 VLP, (H5N1) pandemic influenza vaccine combined with Alhydrogel®, given 21 days apart, at three dose levels: 5µg, 10µg and 20µg., compared to the placebo, (100mM phosphate buffer + 150mM NaCl + 0.01% Tween 80) and combined with Alhydrogel®. | |
| **Primary Endpoints:** | | Safety will be evaluated through reported adverse events, physical examination findings; clinical laboratory results and vital signs. | |
| Statistical Methods for the Primary Objective: | | Safety endpoints will be compared (each vaccine dose level versus placebo) using Fisher’s exact tests. | |
| Secondary Objectives: | | The secondary objective is to evaluate the immunogenicity of two consecutive doses of plant-based H5 VLP vaccine combined with Alhydrogel®, at three dose levels: 5µg, 10µg and 20µg, compared to the placebo, (100mM phosphate buffer + 150mM NaCl + 0.01% Tween 80), combined with Alhydrogel®. | |
| Endpoints | | **Safety and Tolerability**   - Percentage, intensity and relationship of immediate complaints, 2 hours post-vaccination - Percentage, intensity and relationship to vaccination of solicited local and systemic signs and symptoms 7 days following each dose of study vaccine - Percentage, intensity and relationship of solicited and unsolicited local and systemic signs and symptoms 21 days following each dose of study vaccine - Occurrences of all adverse events and serious adverse events - The number and percentage of subjects with normal and abnormal haematological and biochemical values at Screening, Days 21 and 42 - The number and percentage of subjects with normal and abnormal urine values at Screening, Days, 21 and 42   **Immunogenicity**  Geometric mean titres: (GMTs) of hemagglutination inhibition (HAI) antibody on Day 0, Day 21, Day 42. Follow-up serology samples for GMTs will be taken at Day 228. GMTs will be analyzed as follows:   - Seroconversion factor or GMFR (Geometric Mean Fold Rise): is the geometric mean of the ratio of GMTs (Day 21/Day 0 and Day 42/Day 0). - Seroconversion rate: the proportion of subjects in a given treatment group with either a ≥ 4-fold increase in reciprocal HAI titres between Day 0 and Days 21 and 42; or a rise of undetectable HAI titre (i.e. < 10) pre-vaccination, (Day 0) to an HAI titer of ≥40 at Day 21 and 42 post-vaccination. - Seroprotection rate: the proportion of subjects in a given treatment group attaining a reciprocal HAI titer of ≥40 at 21 and 42 days post-vaccination (the percentage of vaccine recipients with a serum HAI titer of at least 1:40 following vaccination). | |
|  | | **Exploratory**  Serum PBMCs will be isolated as part of ongoing research into identifying cellular immune responses. Activation of CD8+ T cells of the adaptive immunity is also important in the clearance of viral infection and this feature is not measured by the measurement of specific antibodies. Blood sample for PBMCs isolation will be taken on Days 0, 21, 42 and 228. | |
| **Methodology/Trial Design:** | | This will be a single-centre, randomized, partially double-blind, dose-escalation, placebo-controlled clinical trial of 48 healthy, male and female subjects, 18 to 60, years of age. Subjects will be screened up to thirty (-30) days in advance of study enrolment, demonstrating a satisfactory baseline medical assessment by history, physical examination, haematological and biochemical analysis (stable health status with no exclusionary medical or psychiatric conditions). Forty-eight (48) study subjects will be randomized into 4 treatment groups of 12, as follows:  **Treatment Group 1**: Consisting of twelve (12) subjects who will receive a 5µg intra-muscular dose of H5 VLP pandemic influenza vaccine on Day 0 and Day 21.  **Treatment Group 2**: Consisting of twelve (12) subjects who will receive a 10µg intra-muscular dose of H5 VLP pandemic influenza vaccine on Day 0 and Day 21.  **Treatment Group 3:** Consisting of twelve (12) subjects who will receive a 20µg intra-muscular dose of H5 VLP pandemic influenza vaccine on Day 0 and Day 21.  **Treatment Group 4:** Consisting of 12 subjects who will be randomized to receive an intramuscular injection of the placebo preparation, 100mM phosphate buffer + 150mM NaCl + 0.01% Tween 80 combined with Alhydrogel® on Days 0 and 21.  Subjects in each dose-escalation step will be observed for 2 hours after each dose for any signs and symptoms of local and systemic intolerance to the study vaccine. Vital signs (BP, HR, RR and oral temperature will be taken/assessed hourly. Any unusual signs or symptoms reported during the initial 2 hours of observation will prompt continued close monitoring. All data will be recorded in the source document during the post observation period.  Subjects will subsequently be telephoned by clinic staff one (1) day, approximately 24 hours post-dose and eight (8) days following each dose administration, to review and record the 7 day safety data. Seven-day safety data for subjects in Cohort 1 and 2 will be tabulated and reviewed by the study investigator and two independent external medical advisors, prior to permitting vaccination of the next dose level. Subjects will return to the clinic on Day 21 and Day 42. At that time they will be asked about the occurrence of any adverse events occurring from Day 8 to Day 21 and Day 29 to Day 42; all such safety data will be recorded in the CRF. At the Day 42 clinic visit, study subjects will be provided a MA and will be instructed to record any adverse events or serious adverse events occurring from Day 42 to Day 228, the end of study. Monthly telephone calls for collection of adverse events or serious adverse events will be made to all subjects following the day 42 visit until the time of the final visit at Day 228. They will be requested to bring their MA with them at the time of the final Visit 5 on Day 228. At the time of the final visit, all safety data will be recorded and a final serology sample for immunogenicity will be taken. Immunogenicity will be assessed in serum samples taken on Days 0, 21, 42 and 228. Cellular immune response assays will be taken on Days 0, 21, 42 and 228. | |
| Planned Sample Size: | | 48 male and female subjects | |
| Vaccination and Specimen Collection Schedules and Duration of Follow-up: | | Vaccination Dose 1 will be administered on Day 0 and Dose 2 will be administered on Day 21. Haematological, biochemical and urine samples for analysis, in addition to urine pregnancy tests on females of child-bearing potential, will be taken at Screening, Day 0 and 21 and 42 days post injection  Serology for GMTs will be performed at Day 0, 21 and 42. Follow-up GMTs will be performed at Day 228 post injection | |
| **Investigational Product, Dose, Mode of Administration, Lot Numbers**: | | Plant-based H5 VLP (virus-like particles) (H5N1) pandemic influenza vaccine adjuvanted with Alhydrogel®, intra-muscular injections of 5µg or 10µg or 20µg of H5 protein  Lot Numbers: | |
| **Reference Therapy:** | | 100mM phosphate buffer + 150mM NaCl + 0.01% Tween 80 and will be combined with the adjuvant Alhydrogel® (aluminium hydroxide) | |
| Inclusion Criteria: | | - Male and female adults, 18 to 60 years of age - Healthy as judged by the Principal Investigator (PI) and determined by medical history, physical examination, vital signs, screening laboratories and medical history conducted no more than 30 days prior to study vaccine administration - BMI of ≥18 and ≤29 - Comprehension of the study requirements, expressed availability for the required study period and ability to attend scheduled visits - Accessible by telephone on a consistent basis - In the opinion of the Investigator, competence and willingness to provide written, informed consent for participation after reading the informed consent form. The subject must have adequate opportunity to discuss the study with an Investigator or qualified designee - If female and capable of child-bearing, have a negative urine pregnancy test result at study entry and agree to employ adequate birth control measures for the duration of the study | |
| Safety Assessments | | Clinical safety methods will include repeated oral temperatures and vital signs; repeated blood chemistry and haematology testing and repeated physical examinations. Seven (7) days following vaccination dose one and two, telephone/memory aid follow-up for solicited local and systemic events and unsolicited adverse events, serious adverse events, doctor’s visits, hospitalizations and any new medications taken or changes in concomitant medication; collection of any adverse events and / or serious adverse events throughout the study period. | |
| Exclusion Criteria: | | - Presence of significant acute or chronic, uncontrolled medical or neuropsychiatric illness. “Uncontrolled” is defined as:   1. Requiring a new medical or surgical treatment within one month prior to study vaccine administration   2. Requiring a change in medication dosage in one month prior to test article administration due to uncontrolled symptoms or drug toxicity (elective dosage adjustments in stable subjects are acceptable), or   3. Hospitalization or an event fulfilling the definition of a serious adverse event within one month prior to test article administration - Any medical or neuropsychiatric condition which, in the Investigator’s opinion, would render the subject incompetent to provide informed consent or unable to provide valid safety observations and reporting - Any confirmed or suspected immunosuppressive condition or immunodeficiency including history of human immunodeficiency virus (HIV) infection or presence of lymphoproliferative disease - Presence of any febrile illness, oral temperature of >38.0 C within 24 hours of test article administration. Such subjects may be re-evaluated for enrolment after resolution of illness - History of autoimmune disease - Administration of any vaccine (including any other influenza vaccine) within a 30 day period prior to study enrolment, or planned administration within the period from the first vaccination up to blood sampling at Day 42 or within 30 days prior to blood sampling at Day 228. Immunization on an emergency basis of a tetanus and diphtheria toxoids adsorbed for adult use (Td) will be allowed provided the vaccine is not administered within two weeks prior to test article administration. Receipt of any other emergency immunizations (e.g. rabies) will result in a case-by-case review of continued participation. - Use of any investigational or non-registered product within 90 days prior to study enrolment or planned use during the study period. Subjects may not participate in any other drug study while participating in this study - Treatment with systemic glucocorticoids at a dose exceeding ≥ 10 mg of prednisone per day, or equivalent for more than 7 consecutive days or for 10 or more days in total, within one month of first test article administration, or any other cytotoxic or immunosuppressant drug or any immune globulin preparation within three months of vaccination. Nasal or inhaled glucocorticoids are allowed - Any significant disorder of coagulation or treatment with coumadin derivatives or heparin. Persons receiving prophylactic anti-platelet medications, e.g., low-dose aspirin, and without a clinically apparent bleeding tendency are eligible - History of previous H5N1 vaccination - History of allergy to any of the constituents of H5 VLP (H5N1) study vaccine, Alhydrogel® (aluminium hydroxide), or the phosphate buffer. - History of severe allergic reactions or anaphylaxis - History of tobacco allergy - Have received a blood transfusion or immunoglobulins within 90 days of study entry - If female, and of childbearing potential, has not been consistently using effective birth control for the 28 days prior to study entry. An example of highly effective birth control is oral contraceptives, hormone implants, abstinence (confirmed by Investigator), or male condom plus spermicide. All female subjects, regardless of birth control history must provide a urine sample for pregnancy screening. Effective birth control must be used for the duration of the study. The subject must have no plan to become pregnant during the study period. Females who are post-menopausal (no spotting at all) for at least one (1) year will not require a urine pregnancy test. - Among female subjects, either known pregnancy or urine beta-human chorionic gonadotropin (ß-hCG) test results consistent with pregnancy prior to test article administration on Day 0 - Female subjects who are lactating - Vital sign abnormalities: systolic blood pressure ≥150 mmHg, diastolic blood pressure ≥90 mmHg, resting pulse rate <40 bpm or >100 bpm - Cancer or treatment for cancer within 3 years of test article administration. Persons with a history of cancer who are disease-free without treatment for 3 years or more are eligible. Persons with treated and uncomplicated basal cell carcinoma of the skin are eligible. | |
| **Immunogenicity Assessments** | | Serology samples for hemagglutination inhibition (HAI) antibody titres taken on Day 0, 21, 42 and 228 | |
| **Statistical Methods** | | Demography and subject characteristics: demographic variables, vital signs, medical history will be summarized by each treatment group  Demography will be compared among treatment groups using Fisher’s exact test for categorical factors and the Kruskal-Wallis test for continuous factors. | |
| **Safety Analysis** | | Safety analyses will be carried out on the Safety Sample, defined as all subjects who received any study treatment  An interim clinical study report analyzing safety and immunogenicity at 42 days post-vaccination will be completed.  Safety endpoints will be compared (each vaccine dose level versus placebo) using Fisher’s exact tests. | |
| **Immunogenicity Analysis** | | Primary immunogenicity analyses will be performed on a Per Protocol Set consisting of subjects who completed the study with no major protocol violation.  The geometric Mean Titers (GMTs) with 95% confidence intervals (CI) will be determined at Day 0, Day 21 and Day 42 for each treatment group. Summary statistics of the immunogenicity endpoints (seroconversion factor (GMFR), seroconversion rate and seroprotection rate) will be calculated for each treatment group at each of Day 21 and Day 42.  Seroconversion factor (GMFR) will be compared between treatment groups using ANOVA at each of Day 21 and Day 42. If a statistically significant treatment effect is found, multiple comparisons will be done using Duncan’s multiple range test.  Seroconversion rates and seroprotection rates will be compared (each vaccine dose level versus placebo) using Fisher’s exact tests at each of Day 21 and Day 42. | |

**TABLE 1** **STUDY VISIT PROCEDURES**

| List of Abbreviations | |
| --- | --- |
| Ab | Antibody |
| ADR | Adverse drug reaction |
| AE | adverse event |
| AR | adverse reaction |
| BCA | Bicinchoninic acid |
| BGTD | Biologics and Genetic Therapies Directorate, Health Canada |
| BMI | Body Mass Index |
| B.P. | Blood Pressure |
| bpm | beats per minute |
| CD | Cluster Differentiation |
| CDM | Clinical Data Management |
| CI | Confidence Interval |
| CPMP | Committee for Proprietary Medicinal Products |
| CRF | Case Report Form |
| DOA | Drug of Abuse (urine) |
| ELISA | enzyme-linked immunosorbent assay |
| EMEA | European Agency for the Evaluation of Medicinal Products |
| FLD | Ferret Lethal Dose |
| GCP | Good clinical practice |
| GLP | Good laboratory practice |
| GMFR | Geometric Fold Rise |
| GMP | Good manufacturing practice |
| GMT | Geometric Mean Titre |
| Heart Rate | HR |
| ICF | informed consent form |
| IEC | Independent Ethics Committee |
| i.m. | intramuscular |
| IRB | Investigational Review Board |
| ITTI | Intent-to-Treat Immunogenicity |
| ITTS | Intent-to-Treat Safety |
| LVLS | last visit of last subject |
| MA | Memory Aid |
| MedDRA | Medical Dictionary for Drug Regulatory Affairs |
| min/mins | minute/minutes |
| mcg | microgram |
| mcl | microlitre |
| ml | millilitre |
| Mg | milligram |
| mm | millimetre |
| mmol | millimole |
| MUHC | McGill University Health Centre |
| OTC | Over-the-Counter |
| PBMCs | Peripheral Blood Mononuclear Cells |
| NA | Neuraminidase |
| nm | nanometre |
| PBS | Phosphate buffered saline |
| PE | physical examination |
| PP | Per-Protocol |
| PV | Pharmacovigilance |
| REB | Research Ethics Board |
| Respiratory Rate | RR |
| SAE | serious adverse event |
| SRID | Single radial immunodiffusion |
| TFF | Tangential Flow Filtration |
| TL | telephone log |
| URTI | Upper Respiratory Tract Infection |
| VLP | Virus-like particles |
| WFI | Water for injection |
| WHO | World Health Organization |

1.0 INTRODUCTION

1.1 Background

Human influenza is an acute respiratory disease that is rooted in the distant past and is one of the most important infectious diseases of mankind. The disease is highly contagious, with person-to-person spread by aerosol droplets that mainly infect the epithelial cells of the respiratory tract.^1^ Pandemics of influenza, occur unpredictably when a novel virus with the capacity for person-to-person transmission emerges to which there is little immunity in the population.^2^

Viral influenza is caused by a pleoimorphic segmented negative strand, an enveloped RNA virus with a particle size of 80-120 nm diameter. The two major antigenic types of virus are influenza A and influenza B. Influenza C virus, the third antigenic type, is less frequently observed and less important as a human pathogen than influenza A and B viruses.^3^ Influenza A viruses are classified into subtypes on the basis of two surface antigens: hemagglutinin (H) and neuramininidase (N). The envelope of the virus consists of a lipid bilayer from which HA and the NA glycoproteins project like spike forms. The virus adheres to the host cells through the HA glycoprotein before fusing with it. The NA prevents viral aggregation and assists in release of newly minted viruses from the infected cell. These glycoproteins are the principal determinants for cell entry in infection (HA) and for exit from the cell after virus replication.^1^

Recently circulating strains have possessed one of three H and one of two N antigens, and the subtypes are designated accordingly (e.g., H3N2, H1N1). Antibodies to these antigens, particularly to H antigen, can protect an individual against a virus carrying the same antigen. During inter-pandemic periods, minor H antigen changes ("drift") are common, and the greater the change the less will be the cross-immunity to the new strain conferred by the previously circulating virus. It is this antigenic variation from one influenza virus subtype to another that is responsible for continued reoccurrence of influenza epidemics, necessitation annual reformulation and administration of influenza vaccine.^4^

Infection with influenza viruses results in a spectrum of clinical responses ranging from an asymptomatic infection to a primary viral pneumonia that rapidly progresses to a fatal outcome. The onset of illness is usually abrupt, with the occurrence of headache, chills, and dry cough that is rapidly followed by high fever, significant myalgias, malaise, and anorexia. Sub-sternal tightness and soreness can accompany the cough. The most prominent sign of infection is fever that often peaks within 24 hours to as high as 41ºC, but more commonly is in the 38ºC to 40ºC range. The fever usually begins to decline on the second or third day of illness and is usually gone by the sixth day of illness; median duration is 3 days. The elderly may experience high fever, lassitude, and confusion without overt respiratory signs. Nasal obstruction, rhinorrhea, and sneezing often occur, and pharyngeal inflammation without exudate is common. Conjunctival inflammation and excessive tearing also may occur. The incubation period can be as short as 24 hours or up to 4 or 5 days, depending in part on the dose of virus and the immune status of the host.

As fever declines, the respiratory signs and symptoms such as rhinorrhea and coughing may become more intense. The cough frequently changes from a dry, hacking nature to one that is productive of small amounts of sputum that are usually mucoid but can be purulent. After the fever and upper respiratory tract symptoms resolve, cough and weakness can persist for 1 to 2 additional weeks. Although primary influenza virus pneumonia with a fatal outcome occurs rarely in healthy adults, it can occur in those with pre-existing cardiopulmonary disease. Most fatalities from influenza are the result of secondary bacterial pneumonia, primary viral pneumonia or combined viral-bacterial pneumonia.^5^

The disease caused by the avian (H5N1) virus follows an unusually aggressive clinical course, with rapid deterioration and high fatality. The incubation period may be longer (ranging from 2 to 8 days and possibly as long as 17 days), than for normal seasonal influenza. The possibility of multiple exposures to the virus makes it difficult to define the incubation period precisely. Initial symptoms include a high fever, usually with a temperature higher than 38ºC and influenza-like symptoms. Diarrhoea, vomiting, abdominal pain, chest pain, and bleeding from the nose and gums have also been reported as early symptoms in some patients. Watery diarrhoea without blood appears to be more common in H5N1 avian influenza than in normal seasonal influenza. The spectrum of clinical symptoms may, however, be broader and not all confirmed patients have presented with respiratory symptoms. Common laboratory abnormalities include leucopenia (mainly lymphopenia), mild-to-moderate thrombocytopenia, elevated aminotransferases and with some instances of disseminated intravascular coagulation.^6^

The recent outbreak with H1N1 has stressed the need for the development of effective vaccines against pandemic strains for which the human population could be naïve. The H1N1 strain originated from swine in Mexico in late April 2009 and due to international travel, by June 15^th^, 76 countries have officially reported 35,928 cases of influenza A/(H1N1) infection, including 163 deaths. It is believed that this strain will circulate in the upcoming months and probably affecting people at a higher attack rate than typical seasonal influenza strains.^7^  Accordingly, vaccine manufacturers have started to produce vaccines for this strain but manufacturing difficulties due to the particular low yield of that strain in embryonated eggs and cell culture of impaired the massive distribution of the vaccine in time for the influenza season.^8^ This situation reinforces the need for alternative manufacturing technologies that are not dependent on the growth of whole live viruses.

Also, if the H1N1 strains mature to full pandemic and starts to infect a large number of people, many scientists fear that this strain could recombine with H5N1 strains and lead to a new H5N1 influenza strain that would acquire the capacity for human-to-human transmission.^7^ This process of reassortment has been demonstrated in laboratory animals between H3N2 and H5N1 strains.^9^ The H1N1 strain now circulates in Indonesia, Asia and Egypt where most human cases of H5N1 are found stressing the need for developing and massively producing pandemic vaccines.

Vaccination against pandemic influenza is considered to be the most effective option to limit its spread.^10^ It will continue to be the primary strategy for preventing influenza-associated deaths. Studies directly comparing outcomes in vaccinated vs. unvaccinated groups have shown that the currently available trivalent inactivated influenza vaccine is approximately 68% effective in preventing deaths from the complications of pneumonia infections.^11^

1.2 Background of the Investigational Product

The H5 VLP (H1N1) pandemic influenza vaccine is produced by transient expression of recombinant proteins, (the hemagglutinin from the A/Indonesia/5/05, clade 2.1, H5N1 strain), in non-transgenic plants, *Nicotiana benthamiana*. *Nicotiana benthamiana* is a wild Australian relative of *N. tabaccum* but has no agronomic or food use. It has been chosen for transient expression mainly because its leaves are amenable to vacuum infiltration of *Agrobacterium*. The A/Indonesia/5/05, clade 2.1, H5N1 strain was selected because it was recommended by the World Health Organization (WHO) as a candidate vaccine, in addition to it being one of the most virulent H5N1 strains, having a mortality rate of 80% with 141 confirmed cases in 2008.

The transfer vector used to insert the plasmids (containing the constructs for proteins of interest) into the plant is a bacterium, *Agrobacterium tumefaciens*. The recombinant protein is expressed by the plant cells as self-assembled virus-like particles or VLPs.

Transient expression is a protein expression technology which does not require (or use) the stable integration of a foreign gene in the host genome. Transient expression can be achieved by various means, but generally implies the use of a “transfer” vector, bacterial, viral or purely mechanical, which mediates the passage of genetic information in the form of a polynucleotide (in this example a strand of DNA) into a host cell. Once in the cell, the genetic information is generally used directly as a template for transcription into mRNA molecules which are subsequently translated into a protein by the host cellular machinery.

A binary plasmid is used to develop its genetic construct. A binary plasmid is a plasmid which can multiply (replicate) in two hosts. For all the work involved in the assembly of genetic constructs, the host used is *E. coli.* This bacterium is used as it has a rapid growth and produces large amounts of plasmids. The binary plasmids consists of regions responsible for replication in the two hosts (*E. coli* and *Agrobacterium*), and of regions located within T-DNA borders. T- DNA stands for “transferable DNA” and it is within these borders that expression cassettes can be inserted. Expression cassettes consist of assemblies of DNA transcription and translation elements, one of which is the gene encoding for a target protein, in this case the H5 protein. When genetic constructs are finished, the resulting plasmid is transferred to *Agrobacterium*. *Agrobacterium* is the ‘transfer’ vector used to pass the genetic information into plant cells. The expression system requires the use of a suppressor of silencing as a helper protein. As transient expression results from the massive synthesis of H5 mRNA molecules, plants react by a silencing mechanism which destroys the nascent H5 mRNA molecules. A suppressor of silencing (in this case HcPro) counteracts this internal protection mechanism and allows the H5 mRNAs to remain undamaged by plant endoribonucleases (enzymes which degrade mRNA molecules). As two proteins are thus required for efficient expression of H5, the technology used for the production of H5-VLPs is described as a “co-expression” technology. This also means that two *Agrobacterium* lines have to be produced and used for efficient transient expression of H5. They will be referred to as H5/Agro and HcPro/Agro in the following descriptions.

The cellular mechanism of transient expression is explained in the following paragraph in the context of the H5 protein but is similar for other proteins although final accumulation points may vary.

When infiltrated in the leaves, *Agrobacteria* (H5/Agro and HcPro/Agro) will attach to plant cells and establish a channel across the plant cell wall and membrane (Type IV secretion channel). While the binary plasmids remain in the attached *Agrobacteria*, a single-strand DNA fragment (ss-DNA) of the genetic constructs contained within the T-DNA borders of the binary plasmid will be excised to form a mobile copy. This mobile copy travels to the plant cell through the channel with the help of molecular elements belonging both to the *Agrobacterium* and plant cells. Thus, at this stage of transient expression, two types of mobile copies will pass to the plant cells, those containing the H5 gene, and those containing the HcPro genes.

In the plant cells, both types of mobile copies will likely be transformed into double-stranded DNA molecules before being transcribed into H5/mRNAs and HcPro/mRNAs. These mRNAs will be translated into H5 and HcPro proteins. As explained above, HcPro proteins are required to insure that as much H5/mRNAs as possible will remain undamaged and be translated into the H5 protein.

As the H5 protein contains a signal peptide, it is co-translationally passed into the endoplasmic reticulum where it is matured into a glycoprotein and then into trimeric assembly of the glycosylated monomers that remain anchored to the endomembrane system. It is postulated that these trimers will follow the endo-membrane system of the secretion pathway and gather at specific locations of the plasma membrane called lipid rafts. These are regions associated with particle movements in and out of the cells. The raft regions of the plasma membrane have a unique lipid composition (rich in sterols) and lack the proteins commonly associated with membranes. Rafts saturated in membrane anchored hemagglutinin trimers will then bud out of the cell and form enveloped nano-particles typical of the influenza virus with the hemagglutinin protruding out of the particle as spikes easily visible in electron microscopy. As plant cells are enclosed by cell walls which are made of tightly assembled cellulose fibers and Ca-pectate layers, the virus-like particles or VLPs will accumulate in indentations of the plasma membrane.

1.3 Pre-Clinical Studies

Animal studies have been conducted with research grade H5 VLP vaccine as follows: three (3) in mice, one (1) study in rats and one (1) study in ferrets. A mouse immunogenicity study has been done in order to compare immunogenicity with GMP grade material from the pilot plant and research grade material. Immunogenicity and safety of the vaccine were evaluated in each study done thus far. In addition, one specific study in mice evaluated the efficacy of the vaccine after a challenge with heterologous H5N1 strains.

All studies used the intramuscular (i.m.) route of administration with the exception of one arm of an early mouse study, which used intranasal administration. While results were promising by the intranasal route, it was decided to focus on development of the i.m. route only. The H5 VLP vaccine contains the HA protein of the A/Indonesia/5/05 strain (clade 2.1). The strains used for the challenge were A/Turkey/582/06 (clade 2.2) and A/Vietnam/1203/04 (clade 1). Studies were done with and without an aluminum hydroxide adjuvant, Alhydrogel®. Adjuvant (2%) was mixed 1:1 with the HA vaccine at least 15 minutes prior to use. While research vaccines differed from GMP lots in both scale and details of manufacturing, the results are supportive of safety and efficacy of the product. Details of the pre-clinical studies conducted in animals with research grade H5 VLP vaccine are presented in Table 2 below.

**TABLE 2 Pre-clinical Studies Conducted with Research Grade Material**

| Study # (timeline) | Animal species (# of animals) | Process used for producing vaccine | Study outline | Primary endpoints | Main findings |
| --- | --- | --- | --- | --- | --- |
| PP-rHA5i-003 (Q3-Q4 07) | Mice (60) | Research grade Clarification bench scale,. Purification using only fetuin affinity chromatography at bench scale. | Intramuscular administration: Doses of 0.1, 1, 5 and 12 µg^[[1]](#footnote-2)^ with Alhydrogel, dose of 5 µg without Alhydrogel, recombinant HA used as control Ag. Two doses administered at 21 day interval  Intranasal administration: Doses of 0.1 and 1 µg with Chitosan, dose of 1 µg without adjuvant. Recombinant HA used as control Ag. Two doses administered at 21 day interval | Immunogenicity: Measurements of HI titers, antibody titers and antibody subtyping  Safety: Body weight measurement, examination of injection site, gross necropsy with particular attention to injection site | Immunogenicity: All tested doses of VLPs induced high antibody and HI titers for the homologous stain (A/Indonesia/5/04) after boost. 100% of animals vaccinated with VLP vaccine showed a HI titer of >1/40. Cross-reactivity with other H5N1 strains have been measured. Doses of 1, 5 and 12 induced similar antibody levels.  Safety: Body weights were similar for all tested vaccines over time. No significant body weight reduction was seen after immunization. Gross necropsy did not reveal anything remarkable at the injection site. |
| PP-rHA5i-004 (Q4 07- Q1 08) | Mice (148) | Research grade Clarification and purification using fetuin affinity chromatography all at bench scale | Intramuscular administration: Doses of 1, 5, and15 µg^[[2]](#footnote-3)^ with Alhydrogel, recombinant HA used as control Ag. Two doses administered at 21 day interval. Challenge with 1000 LD50 of clade 2.2 strain (H5N1 A/Turkey/582/06)  done 21 days after boost immunization. Challenge with 10 LD50 of clade 1 strain (A/VN/1203/04) done 75 days after boost immunization. | Efficacy: Survival rate after challenge with heterologous H5N1 strains (clade 2.2 and 1). Viral loads in lungs and nasal turbinates of animals 5 days after challenge.  Immunogenicity: Measurements of HI titers, antibody titers  Safety: Body weight measurement, examination of injection site, particular attention to injection site at necropsy. Gross necropsy not feasible in P4 laboratory. | Efficacy: All vaccinated mice survived the heterologous challenges. Mice immunised with the VLP vaccine showed a significant lower viral load in the URT or the lungs than non-immunised mice or mice immunised with the control Ag.  Immunogenicity: Mice showed cross-reactive HI titer 70 days after the last boost.  Safety: No difference in body weight was seen between groups. |
| PP-rHA5i-005^[[3]](#footnote-4)^ Q2-Q3 08) | Rats (21) | Research grade Clarification and purification using fetuin affinity chromatography all at bench scale | Intramuscular administration: Dose of 15 µg^[[4]](#footnote-5)^ with Alhydrogel. Two doses administered at 21 day interval. | Immunogenicity: Measurements of HI titers, antibody titers  Safety: Body weight measurement, examination of injection site, gross necropsy with particular attention to injection site | Immunogenicity: The dose of 15 µg induced high HI titers (>1/320) after boost. Antibodies were cross-reactive for other H5N1 strains.  Safety: All examined organs were normal at gross necropsy. Injection site was normal at necropsy. |
| PP-rHA5i-006 (Q2-Q3 08) | Ferrets (50) | Research grade  Clarification done in Pilot Plant using current qualified equipment and current cGMP procedures. Purification using fetuin affinity chromatography made at bench scale. | Intramuscular administration: Doses of 1, 5, 7.5, 15 and 30 µg^[[5]](#footnote-6)^ without Alhydrogel, doses of 1, 5 and 15 with Alhydrogel (mixed 1:1 at time of use). Recombinant HA used as control Ag.  Two doses administered at 21 day interval. A total of 9 groups were in the study. | Immunogenicity: Measurements of HI titers, antibody  Safety: Body weight measurement, examination of injection site, gross necropsy with particular attention to injection site. | Immunogenicity: 93% of ferrets immunized with the VLP vaccine mixed with Alhydrogel showed a HI titre after the first dose; those in the 5ug dose showing a HI titre >1/40. All other adjuvanted dose groups attained >1/40 after the boost. Mean GMT increase for all adjuvanted groups ranged from 7.5 to 15.6 times after dose 1 and from 4-10.8 times after the 2^nd^ dose. Cross-reactivity was measured for ferrets immunised with VLP mixed 1:1 with Alhydrogel. Contrary to the mouse model, no antibody response was seen when VLP was not mixed with Alhydrogel. The15 µg dose group had a lower mean geometric titer than the 5 µg dose group, due to 1 ferret with a unexpectedly low immune response. our investigation did not find a definitive cause for this result. The peak antibody response was achieved at the dose of 5 µg with Alhydrogel.  Safety: Body weights were similar between groups. |
| PP-rHA5i-008 (Q4 08- Q1 09) | Mice (45) | Comparison of GMP and research grade products. First GMP lot made with a change of construct is included in the study. Products on stability program have been injected to confirm immunogenicity over time. | Intramuscular administration: Doses of 0.1, 0.5, 1 µg with Alhydrogel and dose of 2.5 µg^[[6]](#footnote-7)^ without Alhydrogel. Two doses administered at 21 day interval. | Immunogenicity: Measurements of HI titres, antibody titers  Safety: Body weight measurement, examination of injection site, gross necropsy with particular attention to injection site. | Immunogenicity: A dose of 0.1 µg of research grade or GMP vaccine lots induced comparable HI titers in mice. This also confirms that there is no impact in changing the DNA construct on the immune response induced in mice. The GMP vaccine (1 µg + Alhydrogel and 2.4 µg without Alhydrogel) also induced measurable HI titers against the heterologous clade 1 H5N1 strain A/Vietnam/1203/04.  Older research grade vaccine lot and recent GMP lots generally induced comparable HI titers against the homologous H5N1 strain A/Indonesia/5/05.  Safety: All examined organs were normal at gross necropsy. Injection site was normal at necropsy. Body weights were similar for all tested vaccine lots over time. No significant body weight reduction was seen after immunization. |

As discussed, the research grade H5 VLP vaccine is comparable to the GMP scale (pilot plant) product. GMP product with the new construct was included in study PP-rHA5i-008 in mice, however as the H5 VLP vaccine is produced by a novel technology (transient expression, plant-based), two additional pre-clinical studies using material produced by the current process, to support the progression to a Phase 1 clinical study; a GLP safety study in rats and a ferret challenge study have been conducted.

Two additional preclinical studies have conducted with clinical grade material: one immunogenicity and efficacy study in ferrets and one GLP safety study in rats. The main results of the efficacy study are shown in Table 3 and Figure 1. The safety study in rats did not reveal any unexpected vaccine-related toxic effects and H5 VLP vaccine showed a good safety profile.

1.3.1 Ferret Challenge Study

An efficacy study in the ferret model has been conducted using the product made in the pilot plant (GMP) and the main results are shown in Table 3 and Figure 1 below. In this study, groups of immunized ferrets given two doses of 1.8 or 3.7 µg or with PBS (negative control) have been challenged intranasally with 10 FLD_50_ of the A/Vietnam/1293/94 H5N1 strain after the boost injection. Doses of 1.8 and 3.7µg have been selected based on results from the previous ferret study. Two additional groups have been used to test the immunogenicity only of 0.7 and 11.0 µg doses, but these doses were not challenged. As shown in Table 3, two doses of the H5 VLP vaccine induced antibodies that were cross-reactive with heterologous H5N1 strains. Also, ferrets immunized with two low doses of 1,8 or 3,7 µg of the H5 VLP vaccine fully protected ferrets against a lethal challenge done with a heterologous H5N1 strain (Figure 1C). Moreover, ferrets did not show apparent morbidity signs suggesting that the animals were not affected by the challenge even if this was done with a heterologous H5N1 influenza strain (Figure 1A, B and D). These results suggest that the H5 VLP vaccine could offer cross-protection against different H5N1 strains and that this vaccine could be effective even if it does not perfectly match the circulating strain. This could be an important feature of the H5 VLP vaccine that could be used in prime-boost scenarios or to immunize selected groups such as health workers in contact with infected subjects.

The H5 VLP vaccine can induce good HI titers after a single dose which would represent a significant advantage over other pandemic vaccines (data not shown, please refer to Investigator’s brochure). The dosages of 5, 10 and 20 µg to be tested in humans were selected based on a dose-response curve obtained from HI titers obtained in ferrets after a single dose of the vaccine.

**Table 3** Serum HI titers against homologous and heterologous H5N1 strains in ferrets vaccinated with clinical grade material

| Vaccine*^a^* (Dose) | Indo/5/2005 | VN/1203/04 | turkey/Turkey/1/05 | Anhui/1/05 |
| --- | --- | --- | --- | --- |
| VLP (0,7 µg) | 254 (160-1024) | 21 (<8-64) | 32 (<8-160) | 48 (<8-160) |
| VLP (1,8 µg) | 429 (160-2048) | 24 (<8-128) | 121 (32-320) | 74 (<8-256) |
| VLP (3,7 µg) | 382 (160-1024) | 28 (<8-128) | 78 (<8-256) | 78 (20-160) |
| VLP (11,0 µg) | 560 (256-1280) | 42 (16-128) | 106 (40-160) | 97 (64-160) |

*^a^* VLPs were formulated with Alhydrogel 1% (0.5 mg per dose)

*^b^* HI GMT ±SEM measured using horse red blood cells, sera collected 14 days post boost immunization

Figure 1 Protection against lethal challenge of control and vaccinated ferrets after challenge with the A/Vietnam/1203/04 (H5N1) virus. Ferrets were immunized twice with the H5 VLP vaccine (A/Indonesia/5/05) or the placebo and were challenged with 10 FLD_50_ of the A/Vietnam/1203/04 H5N1 strain 45 days after boost injection. (A) Mean body weight temperature (5 ferrets per group) (B) Percentage of body weight loss (at day 6, 3 ferrets were found dead and the 2 remaining were euthanized for human reasons as they have achieved of 20% body weight loss) (C) Survival rate (D) Activity score

(B)

 (B)

(A)

(D)

(C)

All pre-clinical studies done to date demonstrate no issues of safety and tolerability. The immunogenicity results demonstrated that the H5 VLP vaccine induces an antibody response that cross-reacts with different H5N1 strains. This was also evidenced by the survival of all mice and ferrets vaccinated with the H5 VLP vaccine (A/Indonesia/5/05/) when challenged with H5N1 strains of a different clade or sub-clade. Refer to the Investigator’s Brochure for further information.

1.4 Rationale

Dating back to the 1500s, there is evidence of widespread influenza disease occurring three or four times during each century that have been manifested by high levels of morbidity and mortality. Such global epidemics or pandemics are caused by the sudden emergence of new influenza A subtypes with major changes in the hemagglutinin (HA) and / or the neuraminidase (NA) surface proteins, known as antigenic shifts.^12^ Type A Influenza is a highly variable RNA virus with a negative sense segmented genome. These properties make it a highly variable virus which undergoes continuous antigenic drift (minor antigenic variation) and infrequent antigenic shift (major antigenic variation). Fifteen hemagglutinin (H1-H15) and ten neuraminidase (N1-N9) subtype antigens of the Influenza A virus have been recognized in nature, with all sub-type antigens occurring in wild bird reservoirs, particularly ducks and shorebirds. The re-assortment of viral genomes that results in antigenic shifts occurs through the mixing of human influenza strains with either avian or swine influenza viruses^.13^  Influenza A is a disease of immense complexity in which the outcome of illness depends on interactions between a virus of diverse and changing genetic and antigenic composition, and the multi-faceted immune system of the host which may not be able to respond adequately within a restricted time frame. The principal determining factor for whether influenza outbreaks occur is the degree of match in specificities (complementarity) between the HA and NA surface antigens) of the virus and the antibodies against them which are current in the population.^1^ The majority of the world population does not have immunity to the new influenza subtypes.

Pandemic influenza occurred at least three times in the 20^th^ century: 1918/19 (mutated H1N1), 1957-58 (H2N2) and 1968-69 (H3N2). Each was associated with high rates of morbidity, social disruption and high economic costs. The Spanish Influenza pandemic of 1918/19 was the most extreme and strikingly virulent in the last century. 30,000 to 50,000 Canadians were among the estimated 20 to 40 million people who died worldwide. This pandemic was associated with an unusually high fatality rate in young healthy adults, the greatest number of deaths occurring in the 20-40 year-old age group.^14^ The arrival of the Spanish flu was linked to the soldiers returning home from the First World War. As demobilized soldiers headed home first by troopship, then by train, influenza was carried to small communities across the country along trade and transportation routes. The first civilian outbreak was reported in Victoriaville, Quebec on 8 September, 1918. It took approximately one month to traverse the nation from coast to coast. By the end of the winter of 1918-19, it is estimated than one in six people in Canada had contracted the disease.^15^ Death from hemorrhagic viral pneumonia was rapid, occurring within one or two days of symptom onset as documented in local archives. Mortality associated with the more recent pandemics of 1957 (69,800 deaths) and 1968 (33,800 deaths) were confined to elderly and chronically ill persons, and severity was reduced, in part, by administration of antibiotic therapy for secondary bacterial infections and by more aggressive supportive care.^16^

Until 1997, avian influenza viruses were thought not to be directly transmissible to humans. In May – December, 1997, a pathogenic avian influenza A/Hong Kong/97 (H5N1) virus jumped the species barrier resulting in the death of six people and 18 hospital admissions in Hong Kong emerging as a pandemic threat to human beings.^17^ In December 1997, veterinary authorities in Hong Kong slaughtered 1.6 million chickens and subsequently no more human influenza cases caused by avian virus were detected. Although the H5N1 viruses lacked the ability to spread efficiently from person-to-person, there was great concern that viruses with pandemic potential could emerge through genetic re-assortment with human influenza viruses circulating in Hong Kong.^18^

Since the first reported human case of H5N1 infection in 1997, the virus has continued to evolve, but is still lacking efficient human-to-human transmissibility. However, since 2003, there have been 429 human cases confirmed by WHO and 262 deaths associated with the infection. The strain circulating in Indonesia is the most deadly strain with 141 confirmed cases and 115 deaths. Indonesia is no longer reporting human cases since the end of 2008 and it is expected that the number of deaths associated with H5N1 infection has increased. From 2003, human cases of H5N1 infection have now been confirmed in several other countries, Indonesia, Egypt, Vietnam and China having the highest number of cases. As of 2003, the H5N1 virus has spread across Asia and into Europe, the Middle East, India and Africa, with outbreaks occurring in bird populations in more than 60 countries resulting in the massive cull of birds in some cases. The H5N1 virus continues to spread in bird populations increasing the possibility of eventually recombining or mutating and acquiring the ability to infect humans.

Vaccine manufacturers have developed vaccines for H5N1 strains. The first clinical trials with this vaccine demonstrated that conventional split vaccines, made in eggs had poor immunogenicity in humans. Two doses and proprietary adjuvants were required to elicit an adequate immune response.

Given these historical precedents, the current epidemiologic and ecological circumstances, the present level of understanding of influenza viruses and influenza infection, it is reasonable to assume that additional pandemics will occur. In any future pandemic, deaths, hospitalizations and direct costs are still expected to be substantial. Social disruption, interruption of commerce, school closings, and public unrest are likely when many people are ill at the same time.^11^

Limited global vaccine production capacity exists at this time. A 2009 report prepared in collaboration with the WHO and the International Federation of Pharmaceutical Manufacturers and Associations (IFPMA) has concluded that if a pandemic emerged during 2009, the most likely case is that manufacturers could produce 2.5 billion doses globally in the first 12 months after they received the production strain. It would take 4 years to produce enough vaccine to meet total global demand (at two doses for 6.7 billion people). In the best-case scenario, the industry could produce 7.7 billion doses in the first 12 months of a pandemic and could meet global demand in 1½ years. The authors of the report predicted that annual pandemic vaccine production capacity will rise to somewhere between 5 billion and 14.5 billion doses over the next 5 years.^19^

The development of effective vaccines to combat influenza will provide the primary preventative measure against pandemic influenza, despite the difficulties in developing pandemic influenza vaccines. Practical issues to consider include how to provide an adequate vaccine supply quickly and to use the available doses optimally. Plant-based vaccines offer manufacturing advantages as it requires only the genetic sequence coding for the HA protein of the selected virus to initiate vaccine production and no lengthy adaptation processes. Medicago has already demonstrated that it could produce the vaccine for use within 14 days of receiving the DNA sequence (see Medicago’s press release of May 12th 2009). Therefore, this technology can be a significant contributor to providing vaccine rapidly in the event of an influenza pandemic.

2.0 TRIAL OBJECTIVES

2.1 Primary Objective

The primary objective is to assess the safety and tolerability of two consecutive doses of plant-based H5 VLP, (H5N1) pandemic influenza vaccine combined with Alhydrogel®, given 21 days apart, at three dose levels: 5µg, 10µg and 20µg., compared to the placebo, (100mM phosphate buffer + 150mM NaCl + 0.01% Tween 80) and combined with Alhydrogel® 1%.

2.1.1 Primary Endpoints

Safety will be evaluated through reported solicited local events as follows: erthyema (redness), swelling and pain at the injection site. The following systemic symptoms will be solicited; headache, fever, muscle aches, joint aches, fatigue, chills, feeling of general discomfort or uneasiness, swelling in the axilla, groin, neck and chest. The occurrence of any adverse events or serious adverse events, physical examination findings, clinical laboratory results, oral temperature and vital signs will be assessed.

2.1.2 Summary of Statistical Methodology for the Primary Objective

Safety endpoints will be compared (each vaccine dose level versus placebo) using Fisher’s exact tests.

2.2 Secondary Objective

The secondary objective of this study measures the capacity of the H5 VLP vaccine to induce specific antibodies against the virus. This is referred to adaptive immunity and the measurement of specific antibodies represents only a part of the overall immune response that is induced by a vaccine. The levels of antibodies induced by a vaccine will differ according to the vaccine type (split vaccine, whole viruses or VLPs), the selected strain and the age of the subject. The European Committee for Proprietary Medicinal Products has established criteria on which seasonal vaccines obtain licensure in a given country. These criteria are based on the level of antibodies induced and the percentage of subject achieving a target antibody titer. If these criteria are met, a seasonal vaccine should be effective in 70% of the target population. However, in the case of a pandemic with a highly virulent strain, no one can predict if achieving these criteria will provide the same level of protection. In addition, improved efficacy of a given vaccine is desirable in the case of a virus showing a high morbidity and mortality rate as is expected in the case of a severe pandemic. In this clinical trial and in accordance with the most recent EMEA (CHMP) guideline entitled “*Guideline on Influenza Vaccines Prepared from viruses with the potential to cause a pandemic and intended for use outside of the core dossier context*,” February 2007 – EMEA/CHMP/VWP/263499/2006: “all three criteria (seroprotection rate, GMT increase and response rate) as defined below should be fulfilled.”

The secondary objective of this study is to assess the immunogenicity of two doses, 21 days apart, of plant-based H5 VLP pandemic influenza vaccine combined with the adjuvant Alhydrogel® 1%, at three dose levels, 5µg, 10µg and 20µg compared to the placebo, 100mM phosphate buffer + 150mM NaCl + 0.01% Tween 80 combined with Alhydrogel®, based on serum hemagglutination-inhibiting (HAI) antibody response assessed by geometric mean titres (GMTs) elicited following the first and second vaccine dose, respectively. Serum HAI immunogenicity will be assessed in terms of Geometric mean titres (GMTs) of hemagglutination inhibition (HAI) antibody on Day 0, Day 21, Day 42. Follow-up serology samples for GMTs will be taken at Day 228. GMTs will be analyzed as follows:

- Seroconversion factor or GMFR (Geometric Mean Fold Rise): is the geometric mean of the ratio of GMTs (Day 21/Day 0 and Day 42/Day 0).
- Seroconversion rate: the proportion of subjects in a given treatment group with either a ≥ 4-fold increase in reciprocal HAI titres between Day 0 and Days 21 and 42; or a rise of undetectable HAI titre (i.e. <10) pre-vaccination, (Day 0) to an HAI titer of ≥40 at Day 21 and 42 post-vaccination.
- Seroprotection rate: the proportion of subjects in a given treatment group attaining a reciprocal HAI titer of ≥40 at 21 and 42 days post-vaccination (the percentage of vaccine recipients with a serum HAI titer of at least 1:40 following vaccination).

2.3 Exploratory endpoints

More research is being done at characterizing more extensively the type of immune response induced by a given vaccine and how it could correlate with protection and cross-reactivity with other viruses. A vaccine inducing an immune response against drifted strains will present a significant advantage in the event of a pandemic outbreak. Recently, researchers from Novartis^[[7]](#footnote-8)^ have shown that the H5N1 subunit vaccine formulated with the MF59 adjuvant induced a large pool of H5N1 –specific memory B cells and H5-CD4+ T cells that were broadly cross-reactive against drifted H5N1 strains. These authors have also shown that the level of specific CD4+ T cells after the first dose of vaccine predicted the rise in antibody and their persistence over 6 months.

Both the innate and the adaptive arms of the immune system have been the target of vaccine design. Innate immunity is non-specific but is activated very quickly (within minutes) after an infection contrary to adaptive immunity. Innate immunity leads to a rapid burst of inflammatory cytokines and to the activation of antigen-presenting cells. Some adjuvants have been developed in order to trigger innate immunity which will eventually activate adaptive immunity.

Activation of CD8+ T cells of the adaptive immunity is also important in the clearance of viral infection and this feature is not measured by the measurement of specific antibodies.

An investigation of the mechanisms of action of the plant-made H5 VLP vaccine by isolating PBMCs from subjects and by characterizing the subset of cells that are induced will be conducted.

3.0 INVESTIGATOR/S AND TRIAL ORGANIZATION

**Study Centre**

Brian Ward, M.D, Principal Investigator

MUHC Vaccine Study Centre

14770 Pierrefonds Blvd., Suite 204

Pierrefonds, Québec H9H 4Y6

Deirdre McCormack, MUHC Vaccine Study Centre Manager

14770 Pierrefonds Blvd., Suite 204

Pierrefonds, Québec H9H 4Y6

**Clinical Chemistry and Haematology Laboratory**

McGill University Health Centre - Montreal Children's Hospital
2300 Tupper, C403,
Montreal, Qc H3H 1P3

**Serological Laboratory**

Michèle Dargis

Manager, Analytical Development

Medicago R&D Inc.

1020, route de l’Eglise, bureau 600

Sainte-Foy, Québec G1V 3V9

**Clinical Study Management**

Medicago R & D Inc.

1020, route de l’Eglise, bureau 600

Sainte-Foy, Québec G1V 3V9

Project Leader: Sonia Trepanier

**Data Safety Monitoring**

Brian Ward, M.D, Principal Investigator

MUHC Vaccine Study Centre

14770 Pierrefonds Blvd., Suite 204

Pierrefonds, Québec H9H 4Y6

Dr. Scott Halperin

Canadian Centre for Vaccinology

Dalhousie University – IWK Health Centre

5850/5980 University Avenue

P.O. Box 9700

Halifax, Nova Scotia B3K 6R8

**Data Management**

PharmaNet Specialized Pharmaceutical Services (SPS)

415 McFarlan Road, Suite 201

Kennett Square, PA 19348

Project Manager:  Derya Puchalski

**Biostatistics and Medical Writing**

Anapharm Inc.

2500 Einstein

Québec (Québec) G1P 0A2

Senior Project Manager: Yan Grimard

Senior Biostatistician, Biometry: Claude Lapointe

**Clinical Monitoring**

AC BioResearch Inc.

389 Woodcroft

Hudson, Québec. J0P 1H0

Clinical Monitor: Gail Clements

4.0 INDEPENDENT ETHICS COMMITTEE (IEC) / INSTITUTIONAL REVIEW BOARD (IRB)

4.1 Ethical Conduct of the Study

The Independent Ethics Committee for this study will be the McGill University Health Centre (MUHC) Biomedical D Research Ethics Board.

The study will be conducted in accordance with the ethical principles that have their origins in the most recent update of the Declaration of Helsinki, International Conference on Harmonization (ICH) Guideline E6, GCP rules and applicable Canadian regulatory requirements.

5.0 INVESTIGATIONAL PLAN

5.1 Description of the Overall Trial Design and Plan

This will be a single-centre, randomized, partially double-blind, dose-escalation, placebo-controlled clinical trial of 48 healthy, male and female subjects, 18 to 60, years of age. Subjects will be screened up to thirty (-30) days in advance of study entry and will demonstrate a satisfactory baseline medical assessment by history, physical examination, haematological and biochemical analysis (stable health status with no exclusionary medical or psychiatric conditions). Study subjects will be randomized into 4 treatment groups of 12. Treatment Groups 1, 2 and 3 will be comprised of twelve (12) subjects each who will receive a 5µg, 10µg or 20µg intra-muscular dose of H5 VLP (H5N1) pandemic influenza vaccine on Day 0 and Day 21. Treatment group 4 consisting of 12 subjects, will be randomized to receive an intramuscular injection of the placebo preparation, 100mM phosphate buffer + 150mM NaCl + 0.01% Tween 80 combined with Alhydrogel® 1 % (final concentration of 0.4%) on Days 0 and 21.

Study subjects in each dose-escalation step will be observed for at least 2 hours after each dose for any signs and symptoms of local and systemic intolerance to the study vaccine. Vital signs (BP, HR, RR and oral temperature will be taken/assessed hourly. Any unusual signs or symptoms reported during the initial 2 hours of observation will prompt continued close monitoring. All data will be recorded in the source document during the post observation period. Solicited local and systemic events, as well as any unsolicited adverse events will be recorded by subjects in a Memory Aid (MA) for the 21 days following each administration of vaccine. Seven-day safety data for all subjects will be tabulated and reviewed by independent, external medical advisors, prior to permitting vaccination to proceed to Treatment Group 2 and 3. Subjects in all treatment groups will return to the clinic on Day 21; at that time they will be asked about the occurrence of solicited local and systemic events, as well as any unsolicited adverse events occurring from Day 7 to Day 21; all such data will be recorded. Following administration of the second vaccination on Day 21, subjects will be provided a second Memory Aid for the next 21 days and will be requested to record solicited local and systemic events and any unsolicited adverse events. Seven-day safety data for all subjects will then be collected, tabulated and reviewed by independent, external medical advisors. Subjects will return to the clinic on Day 42 and will be asked about the occurrence of any adverse events occurring from Day 28 to Day 42. At Day 42, study subjects will be provided a Memory Aid and will be instructed to record any spontaneous events occurring from Day 42 to Day 228, end of study. Monthly telephone calls for collection of adverse events or serious adverse events will be made to all subjects following the day 42 visit until the time of the final visit at Day 228. Subjects will be requested to bring their Memory Aid with them upon their return to the clinic 6 months (Day 228) post injection. At the time of the final visit, all safety data will be recorded.

Serology samples assessing Geometric mean titres (GMTs) of hemagglutination inhibition (HAI) antibody) will be taken from all study subjects at pre-dosing on Day 0, in addition to Day 21, and Day 42. Follow-up serology for GMTs will be taken at Day 228.

Cellular immune response assays will also be taken on Days 0, 21, 42 and 228.

5.1.1 Trial Design

Subjects will be screened up to thirty (-30) days in advance of study enrolment, and will demonstrate a satisfactory baseline medical assessment by history, physical examination, haematological and biochemical analysis (stable health status with no exclusionary medical or psychiatric conditions). Forty-eight (48) study subjects will be randomized into 4 treatment groups of 12, described in Table 4 below:

**Table 4 Study Design**

| ***Test Material*** | ***Treatment Group*** | ***No. of Subjects*** | ***Dose Level*** | ***Administered on Day*** |
| --- | --- | --- | --- | --- |
| Plant-based H5 VLP (H5N1) pandemic influenza vaccine combined with Alhydrogel® 1% | Treatment Grp 1 | 12 | 5 µg | 0 / 21 |
| Plant-based H5 VLP (H5N1) pandemic influenza vaccine combined with Alhydrogel® 1% | Treatment Grp 2 | 12 | 10 µg | 0 / 21 |
| Plant-based H5 VLP (H5N1) pandemic influenza vaccine combined with Alhydrogel® 1% | Treatment Grp 3 | 12 | 20 µg | 0 / 21 |
| 100mM phosphate buffer + 150 mM NaCl + 0.01% Tween 80 combined with Alhydrogel®1% | Treatment Grp 4 (Placebo) | 12 | N/A | 0 / 21 |

The first cohort will consist of sixteen subjects; 12 subjects will be randomized to receive a 5µg dose of plant-based H5 VLP (H5N1) pandemic influenza vaccine and 4 subjects will be randomized to receive the placebo preparation. A telephone call to obtain safety data and to remind study subjects to continue completing the Memory Aid will be made at Day 1 (~24 hours post vaccination). Eight (8) days following each vaccination, subjects will be telephoned by clinic staff to review and record safety data. Seven-day (7) safety data for subjects in the first cohort will be tabulated and reviewed by the Investigator and 2 medical advisors, prior to permitting the second cohort to receive a higher dose (10µg) of study vaccine. The same procedure will be repeated with the second cohort of sixteen subjects. Following a favourable review of the second cohort’s seven-day safety data by the medical advisors, cohort 3 subjects will be permitted to be randomized to the highest dose level (20µg). There will be a total of four (4) seven-day safety reviews by the Investigator and medical advisors.

The safety experts will be provided the following blinded solicited local and systemic symptoms tabulated for the first 7 days post-vaccination: erythema (redness), swelling and pain at the injection site. The following blinded systemic symptoms will be provided for review: occurrence of headache, fever, myalgia (muscle aches), arthralgia (joint aches), fatigue, chills, and feelings of general discomfort or uneasiness, swelling in the axilla, groin, neck or chest, as well as the occurrence of any adverse or serious adverse events. Oral temperature results from the first seven days post vaccination will also be provided. These data may be un-blinded based on the reviewer’s medical judgement. Refer to Section 9.1.8 for further information.

5.1.2 Trial Design and Plan

Subjects will be enrolled in the study in a staggered fashion. Refer to Figure 2 below. Study enrolment will begin with two (2) subjects from Cohort 1 on Day 0; one subject will be randomized to receive the 5µg dose of plant-based H5 VLP (H5N1) pandemic influenza vaccine combined with Alhydrogel® intramuscularly and one subject will be randomized to receive the placebo preparation, 100mM phosphate buffer + 150mM NaCl + 0.01% Tween 80 combined with Alhydrogel®. Subsequently, the following day, 3 subjects will be randomized to receive H5 VLP (H5N1) pandemic influenza vaccine and 1 subject will be randomized to receive the placebo preparation. On Day 2 of the study, 4 subjects will be randomized to receive H5 VLP (H5N1) pandemic influenza vaccine and 1 subject will be randomized to receive the placebo preparation. On Day 3 of the study, 4 subjects will be randomized to receive H5 VLP (H5N1) pandemic influenza vaccine and 1 subject will be randomized to receive the placebo preparation, for a total Cohort of 16 subjects. Once all Cohort 1 subjects have completed Day 7, a blinded safety report will be generated and reviewed by a panel consisting of the Investigator and two independent, expert medical advisors. A favourable 7-day safety report will permit the initiation of the vaccination of Cohort 2 at the 10µg dose level. A second blinded safety report will be conducted following the second vaccination at the 5µg dose level. A blinded seven-day safety report evaluating the safety of subjects receiving the 10µg dose will be generated in the same manner, in addition to a safety report being generated seven days following the second 10µg dose of study vaccine. Study vaccine dose escalation to 20µg will only be permitted if safety data is favourable, as judged by the Investigator and medical safety experts. Safety reports will be generated following the first and second dose of vaccine at the 5 µg and 10µg dose levels, and after the first dose of the 20µg dose level is completed, for a total of 5 reports.

The proposed randomization schedule is described below.

**Figure 2 Enrolment Schedule**

| 0 1 2 3…  10-12 13 14 15  … 21 22-24 25 26 27…  33 à 36  42 à 45 à 48  54 à 57  66 à 69  78 Interim Study Rpt.  228  1s 3s 4s 4s  1p 1p 1p 1p  **Cohort 1**  **5 µg**  Safety Rev # 1  **Co - 1**  Serology Sample and Vaccine #2  **Co 1**  blood draw  5 µg  Serology Sample and Vaccine # 2  **Co 2**  Serology Sample and Vaccine # 2  **Co 3**  blood draw  10 µg  blood draw  20 µg  Last blood draw  1s 3s 4s 4s  1p 1p 1p 1p  **Cohort 2**  **10 µg**  Safety Rev # 1 Co - 2  1s 3s 4s 4s  1p 1p 1p 1p  **Cohort 3**  **20 µg**  - 242  **End of**  **Study**  s; subject, p; placebo, 6 months ~ 160 days |
| --- |

5.1.3 Inclusion Criteria

- Male and female adults, 18 to 60 years of age
- Healthy as judged by the Principal Investigator (PI) and determined by medical history, physical examination, vital signs, screening laboratories and medical history conducted no more than -30 days prior to study vaccine administration
- BMI of ≥18 and ≤29
- Comprehension of the study requirements, expressed availability for the required study period and ability to attend scheduled visits
- Accessible by telephone on a consistent basis
- In the opinion of the Investigator, competence and willingness to provide written, informed consent for participation after reading the informed consent form. The subject must have adequate opportunity to discuss the study with an Investigator or qualified designee
- If female and capable of child-bearing, have a negative urine pregnancy test result at study entry and agree to employ adequate birth control measures for the duration of the study

5.1.4 Exclusion Criteria

- Presence of significant acute or chronic uncontrolled medical or neuropsychiatric illness. “Uncontrolled” is defined as:
- Requiring a new medical or surgical treatment within one month prior to study vaccine administration
- Requiring a change in medication dosage in one month prior to test article administration due to uncontrolled symptoms or drug toxicity (elective dosage adjustments in stable subjects are acceptable), or
- Hospitalization or an event fulfilling the definition of a serious adverse event within one month prior to test article administration
- Any medical or neuropsychiatric condition which, in the Investigator’s opinion, would render the subject incompetent to provide informed consent or unable to provide valid safety observations and reporting
- Any confirmed or suspected immunosuppressive condition or immunodeficiency including history of human immunodeficiency virus (HIV) infection or presence of lymphoproliferative disease
- Presence of any febrile illness, oral temperature of >38.0 C within 24 hours of test article administration. Such subjects may be re-evaluated for enrolment after resolution of illness
- History of autoimmune disease
- Administration of any vaccine (including any other influenza vaccine) within 30 days before study enrolment or planned administration within the period from the first vaccination up to blood sampling at Day 42 or within 30 days prior to blood sampling at Day 228. Immunization on an emergency basis of a tetanus and diphtheria toxoids adsorbed for adult use (Td) will be allowed provided the vaccine is not administered within two weeks prior to test article administration. Receipt of any other emergency immunization (e.g. rabies) will result in a case-by-case review of continued participation.
- Use of any investigational or non-registered product within 90 days prior to study enrolment or planned use during the study period. Subjects may not participate in any other drug study while participating in this study

⦁ Treatment with systemic glucocorticoids at a dose exceeding ≥ 10 mg of prednisone per day, or equivalent for more than 7 consecutive days or for 10 or more days in total, within one month of first test article administration, or any other cytotoxic or immunosuppressant drug or any immune globulin preparation within three months of vaccination. Nasal or inhaled glucocorticoids are allowed

- Any significant disorder of coagulation or treatment with coumadin derivatives or heparin. Persons receiving prophylactic anti-platelet medications, e.g., low-dose aspirin, and without a clinically apparent bleeding tendency are eligible
- History of previous H5N1 vaccination
- History of severe allergic reactions or anaphylaxis
- History of allergy to any of the constituents of H5 VLP (H5N1) study vaccine, Alhydrogel® (aluminium hydroxide), or the phosphate buffer.
- History of tobacco allergy
- Has received a blood transfusion or immunoglobulins within 90 days of study entry
  - If female, and of childbearing potential, has not been consistently using effective birth control for the 28 days prior to study entry. An example of highly effective birth control is oral contraceptives, hormone implants, abstinence (confirmed by Investigator), or male condom plus spermicide. All female subjects, regardless of birth control history must provide a urine sample for pregnancy screening. Effective birth control must be used for the duration of the study. The subject must have no plan to become pregnant during the study period. Females who are post-menopausal for at least one (1) year (no spotting at all) will not require a urine pregnancy test.
- Among female subjects, either known pregnancy or urine beta-human chorionic gonadotropin (ß-hCG) test results consistent with pregnancy prior to test article administration on Day 0
- Female subjects who are lactating
- Vital sign abnormalities: systolic blood pressure ≥150 mmHg, diastolic blood pressure ≥90 mmHg, resting pulse rate < 40 bpm or > 100 bpm
- Cancer or treatment for cancer within 3 years of test article administration. Persons with a history of cancer who are disease-free without treatment for 3 years or more are eligible. Persons with treated and uncomplicated basal cell carcinoma of the skin are eligible

5.1.5 Study Visit Procedures

5.1.5.1 Screening Procedures (up to 30 days prior to study vaccination)

The following procedures will be performed either at screening or in combination with Day 0:

- Review and signature of the Informed Consent Form (ICF). The Investigator, or his/her designee, will fully inform the subject of the nature and scope of the study, potential risks and benefits of participation, the study procedures involved and will answer all questions for the subject prior to requesting the subject’s signature on the ICF. Informed consent will be obtained prior to performing any study-related procedures. Source documentation of the ICF being signed prior to any study-related procedures being performed is necessary and a copy of the ICF must be provided to the subject.
- Collect and review demographic data and medical history. Demographic data includes sex, date of birth, age, body mass index, weight and height, race and ethnicity and current drug and alcohol use. The medical history should record significant problems active at the time of screening and within the prior year. Problems that have been clinically inactive within the prior year, but which might alter the subject’s current or future medical management, should also be noted (e.g. known mitral valve prolapse or history of seizure disorder). Completely resolved past problems with no impact on current medical management (e.g. a healed fracture) may be omitted.
- Review and record current and previous (up to 30 days prior) medication use.
- Review of inclusion and exclusion criteria.
- Record influenza immunizations received within 24 months prior to the administration of study vaccine; record any adverse reactions following these immunizations.
- Perform vital signs measurements, including oral temperature, heart rate and resting blood pressure. Blood pressure will be taken after the participant has been in a seated position for 3 minutes. Blood pressure should be taken by cuff (manual or automated is acceptable), although the same method should be used throughout the study. Screening measurement of blood pressure may be repeated one time following a 5-minute resting period in a seated position, if judged necessary. All values are to be recorded in the source documentation with the reason for repeating. Pre-vaccination blood pressure results must be found to be within the following ranges: systolic blood pressure is systolic, ≤150 mmHg and diastolic ≤90 mmHg. Heart rate or pulse must be > 40 beats per minute (bpm) and <100 bpm.
- Screening blood samples (20 mL) for biochemistry and haematology analysis will be taken.
- Perform urinalysis (dip stick) on all subjects.
- Perform urine pregnancy testing all females of child-bearing potential participants. If screening and treatment are not done on the same day, an additional pregnancy test must be done prior to treatment on Day 0.

5.1.5.2 Dosing Visit (Day 0)

Emergency equipment will be available on site and appropriate treatment instituted as soon as possible in the event of anaphylaxis or any other immediate hypersensitivity reaction. Medical site personnel will be on site for vaccine administration and remain for at least 2 hours after administration of the vaccine to the last subject. .The Investigator (or medical designee) will be on call for the remainder of the study. If necessary, a physician should be immediately available at the clinical site to administer treatment or to apply procedures for any immediate adverse event(s).

The following procedures will be performed:

- Record changes in medical history and medications and confirm that the subject continues to meet all inclusion and no exclusion criteria since screening
- General physical examination of the major body systems, measurement of vital signs, BMI, weight (kg) / [height (m)]^2^ or weight (lb) / [height (in)]^2^ X 703
- Record vital signs
- Obtain an oral temperature (ºC) using a digital thermometer
- Perform urine pregnancy testing on all female participants of child-bearing potential. No study vaccine will be administered until a negative result is obtained and documented.
- Once eligibility is confirmed, the subject will be randomized to treatment or placebo
- Pre-vaccination blood samples of approximately 10mL for serology and 20mL for cellular immune assays will be drawn
- Document the assigned randomization number. The study vaccine will be administered intramuscularly (i.m.) into the deltoid muscle of the non-dominant arm using a needle of sufficient length to reach the substance of the muscle (at least 1 inch or 2.5 cm or longer based on the subject’s weight). Whenever possible, the injection will be given in the opposite arm from which blood was drawn.

5.1.5.3 Minimum 2-hour Post-Dose Observation Period

- Subjects will remain in clinic for at least 2 hours post-vaccination.
- During the observation period in clinic, vital signs (BP, HR, RR and oral temperature will be taken/assessed hourly. Any unusual signs or symptoms reported during the initial 2 hours of observation will prompt continued close monitoring All data will be recorded in the source document during the post observation period.
- During the observation period, subjects will be provided a Memory Aid (MA), a template for measuring solicited local reactions and an oral digital thermometer to record daily temperature in degrees Celsius. The clinic staff will provide training to the subjects during this time. The subjects will be:
  - Instructed on how to measure their oral temperature with the digital thermometer supplied to them for purposes of the study. From the evening of Day 0 to Day 7 following each vaccination, temperature will be taken at approximately the same time each evening and the findings will be recorded in the MA.
  - Instructed by study staff on how to measure erythema (redness) and swelling 0-20 mm, 21-50 mm and > 50 mm diameter at the injection site using the simple template supplied and to evaluate pain at the injection site. Local tolerance reactions will be assessed every day starting from the evening the injection was administered on Day 0 and continuing and up to Day 7 following each vaccination and recorded on the MA.
  - Requested to grade, on a daily basis from the evening of Day 0 through to Day 7 following each vaccination, each of the solicited systemic reactions as described in Appendix A, the MA.
  - Requested to record their individual data in their MA as described above
  - Requested to use their MA to support their recall in providing information during the scheduled telephone interview conducted on Days 1 and 8 following each vaccination
  - Advised that they will be asked about the occurrence of any symptoms or events requiring medical attention and the use of concomitant medication during each 21-day post dose period as well as throughout the 160 day follow-up period
  - Requested to bring their MA to each clinic visit. The completed MA will not be collected by the study site.
  - Advised on emergency contact information and instructions for contacting study personnel. Subjects will be advised to immediately contact the Investigator, or his designee, in the event of a serious adverse event or medical emergency. Subjects will be provided with a telephone contact number and be instructed to call if any reaction to vaccination is significant or concerning.
  - Advised to notify their health care professional(s) (e.g., primary care physician) that they are participating in a clinical research study of an influenza vaccine.
  - Provided with a date and time to return to the clinic for the Day 21 and Day 42 follow-up visits.

5.1.5.4 Days 1 and 8 Telephone Contact (acceptable interval +/- 1 day), following vaccination 1 and 2)

- Subjects will be asked to use their MA to support their recall in providing information during the scheduled telephone interview conducted on Days 1 and 8 following each vaccination
- Telephone calls will be conducted at Days 22 and 29 (one and eight days following the second vaccination).
- The highest oral temperature, the greatest dimension of local redness and swelling, and the highest symptom grade experienced for each solicited local and systemic reaction from Day 0 to Day 1 and Day 2 – Day 7, will be ascertained and recorded.
- Unsolicited adverse events and/or solicited local and systemic reactions of greater than (>) grade 2 (moderate) severity will be reported promptly (24-48 hours) to the Investigator, who may request the subject come to the clinic for evaluation at his/her discretion. The Investigator will document review of these AEs on the source document.
- If any of the solicited local or systemic reactions noted at the Day 8 telephone contact persist beyond Day 7, these will be considered an AE. The subject will be requested to note when the AEs resolve and report this information to the Investigator or clinic staff at the Day 21 or Day 42 visit.
- Subjects will also be asked about any difficulties in completing the MA, any change in health, any visits to health care facilities and / or medical practitioners and use of any concomitant medications

The subject will be reminded of the date and time of their Day 21 and Day 42 visits.

- - - 1. Day 21 Visit, second vaccination, (acceptable interval +/- 2 days) and

Day 42, (acceptable interval +5 days)

The following procedures will be performed:

- Oral temperature in ºCelsius will be taken and recorded.
- Blood samples of approximately 20mL for biochemistry and haematology, 10mL for serologic testing and 20mL for cellular immune response assays will be taken.
- A urine pregnancy test on all female subjects of child-bearing potential.
- Subjects will be asked about any change in their health, any visits to health care facilities and / or medical practitioners and use of any concomitant medications
- Physical examinations will not be routinely performed at Day 21 or Day 42 visits, unless new complaints or concerns are raised by either the study subject or study staff, and if deemed to be necessary by the Investigator.
- On Day 21, subjects will be vaccinated in the same manner and with the same dose of vaccine (or placebo) that they received at the Dosing visit (Day 0).
- At the Day 21 visit, subjects will again be instructed to take and record their oral temperature on a daily basis for 7 days at approximately the same time each evening and the findings will be recorded in the MA. Subjects will be asked to take their temperature at any other time they feel feverish and to record the highest temperature of the day in the Memory Aid. If the subject becomes feverish (defined as a temperature of >38.0ºC degrees Celsius, they may take OTC antipyretics (acetaminophen, aspirin or ibuprofen) and will be advised to increase the frequency of their temperature measurements to approximately every four (4) hours, until no longer febrile.
- Provision of the subject with a new MA for the collection of solicited local and systemic events and any adverse events occurring following the Day 21 visit and a date and time for the Day 42 visit (Acceptable interval is + 5 days).
- Telephone calls will be conducted at Days 22 and 29 (one and eight days following the second vaccination).
- Provision of a new MA for collection of safety data at the Day 42 visit and a date and time for the final visit at Day 228.
- Monthly telephone calls for collection of adverse events or serious adverse events will be made to all subjects following the day 42 visit until the time of the final visit at Day 228.

5.1.5.6 Final Visit - Day 228 (Acceptable interval is +14days)

The following procedures will be performed:

- A blood sample of approximately 10mL will be drawn for influenza serology testing to evaluate antibody persistence for approximately 6 months following vaccination. A final 20 mL blood sample will also be collected for cellular immune assay analysis. For females of child-bearing potential, a urine pregnancy analysis will be performed.
- Study subjects will be asked to bring their MAs to the visit and will be asked about any change in their health, any visits to health care facilities and / or medical practitioners and use of any concomitant medications since the last contact.

5.2 Discussion of the Trial Design

This study is a randomized, partially double-blind, placebo-controlled, dose-escalation study evaluating two doses, 21 days apart and three dose levels of plant-based H5 VLP (H5N1) influenza vaccine compared to the placebo, 100mM phosphate buffer + 150mM NaCl + 0.01% Tween 80 combined with Alhydrogel® (aluminium hydroxide) in study subjects 18 – 60 years of age.

5.3 Selection of the Trial Population

5.3.1 Recruitment Procedures

Subjects enrolled in this study will be healthy adults, aged 18 to 60 years and will be volunteer members of the community at large. Following IRB approval, potential subjects will be contacted and will be invited to participate in this trial, if they meet all inclusion criteria and do not meet any exclusion criteria.

5.3.2 Participant Information and Consent

Voluntary written informed consent must be obtained from each subject prior to performing any study-related procedures. Each subject should be given both verbal and written information describing the nature and duration of the clinical study. The informed consent process should take place under conditions where the subject has adequate time to consider the risks and benefits associated with his/her participation in the study. Subjects should not be screened or treated until they have signed an approved informed consent form written in a language that is understandable to the subject.

The Independent Research Ethics Committee (IEC/IRB) approved informed consent form should be signed and dated by the subject and the physician (or their nominated designee) who conducted the informed consent discussion. Each subject should receive a copy of the signed and dated written informed consent form along with any other written information provided to the subject.

The Investigator is responsible for assuring the appropriate content of the informed consent form and that informed consent is obtained from each subject in accordance with all applicable regulations and guidelines. The original signed informed consent form should be retained in the Investigator’s files.

The Investigator should maintain a log of all subjects who sign the informed consent form and indicate if the subject received study drug or, if not, the reason why. The subject’s available medical records should also document that the informed consent form was signed and dated prior to any study-related procedures being performed.

5.3.5 Temporary Contraindications

- Temperature >38.0ºC.
- Acute cold symptoms such as minor URTI symptoms that typically resolve in 48-72 hours.

5.3.6 Removal of Participants from Treatment or Assessment

Subjects will be advised that they are free to withdraw from the study at any time without prejudice to their future medical care by the physician or the institution. Subjects who withdraw or are withdrawn from the study after dosing will not be replaced. The primary reason for withdrawal must be recorded in the subject’s medical record and captured in the CRF.

5.3.6.1 Conditions for Withdrawal

Every reasonable effort should be made to ensure that each subject complies with the protocol and completes all study visits. However, a subject may withdraw or be withdrawn from participation if:

- The subject withdraws consent, or
- In the opinion of the Investigator the subject displays non-compliance to the terms of their participation in the clinical trial, or
- Safety reasons as judged by the Investigator and / or Medicago R&D Inc., or
- BGTD, Health Canada or the IRB/IEC terminates the clinical trial.

In case of withdrawal due to a serious adverse event, a subject will not be replaced. Drop-outs will be those subjects who leave the study earlier than planned for whatever reason; drop-outs will not be replaced. Drop-outs who receive at least one dose of study medication will be asked to return to the study site for a final assessment. All drop-outs must be reported to Medicago R&D Inc. The reason for drop-out should be documented in the subjects’ records and in the appropriate section of the CRF. The CRF must be completed up to and including the time of drop-out/final assessment.

5.3.6.2 Lost to Follow-up Procedures

Every attempt will be made to contact study subjects who are lost to follow-up. At least 3 telephone contacts will be attempted and recorded in the source document. As a last resort, one registered letter requesting contact with the site will be sent to any subject with whom the clinic staff no longer has contact.

5.3.6.3 Screen Failures

Screen failures are subjects that have signed the Informed Consent Form, but who were not randomized due to failure on one or more inclusion or exclusion criteria. Eligible/not treated subjects are subjects that have signed the Informed Consent Form and are eligible for enrolment, but were either not randomized or randomized and not treated. Tracking of both classes of screen failures will be carried out by the completion of the demography and termination CRF pages. Neither class of subjects will receive a safety follow-up.

5.3.6.4 Follow-up of Discontinuations

Subjects who receive at least one study vaccine and are discontinued will be followed for safety until the end of study, Day 228, if so allowed by the subject.

5.3.7 Medical History

The medical history should record significant problems active at the time of screening and within the prior year. Problems that have been clinically inactive within the prior year, but which might alter the subject’s current or future medical management, should also be noted (e.g. known mitral valve prolapsed or history of seizure disorder). Completely resolved past problems with no impact on current medical management (e.g. a healed fracture) may be omitted.

5.4 Modification of the Trial and Protocol

Modifications to the protocol should be made as an amendment and should be approved/acknowledged both by Medicago R&D Inc. and the IEC/IRB prior to being implemented unless the amendment is made to eliminate an immediate hazard to the clinical study subjects. Medicago R&D Inc. shall be responsible for notifying the regulatory authority, BGTD, Health Canada, of any substantial amendments to the protocol.

The Investigator is responsible for notifying the IEC/IRB of all protocol amendments.

Administrative amendments (e.g. changes in telephone number, etc.) will not require approval by the IEC/IRB unless requested by them.

5.5 Interruption of the Trial

The study may be interrupted or terminated by the Investigator following consultation with the Sponsor, by the Sponsor, or by regulatory authorities. The Investigator will immediately, on discontinuance of the clinical trial at the clinical site, inform both the clinical trial subjects and the IRB/IEC responsible for the study of the discontinuance, provide them with reasons for the discontinuance and advise them in writing of any potential risks to the health of the clinical trial subjects or other persons. It is the Sponsor’s responsibility to report discontinuance of the study to the regulatory agencies, within the appropriate timeframe and providing them with the reasons for the discontinuance, and advise them in writing of any potential risks to the health of clinical trial subjects or other persons. The Sponsor must then inform the Investigator that the appropriate notifications were made.

6.0 TREATMENTS

6.1 Vaccines Administered

This is a study of two (2) i.m. doses of plant-based H5 VLP (H5N1) pandemic influenza vaccine, at three dose levels (5µg, 10µg or 20µg), administered 21 days apart. Each vial of study vaccine will be combined with Alhydrogel® 1% (aluminium hydroxide, composed of Alhydrogel® 2% from Brennentag diluted in a ratio 1:1 with sterile water).

6.2 Identity of Investigational Product

The investigational product is a H5N1 (virus strain A/Indonesia/5/05, clade 2.1) pandemic influenza vaccine consisting of hemagglutinin (H5) protein VLPs produced in a plant-based *(N. benthamiana)* transient expression system. The transfer vector used to insert the plasmids (containing the constructs for proteins of interest) into the plant for expression is a bacterium, *Agrobacterium tumefaciens.*

Transient expression is a protein expression technology which does not require or use the stable integration process of a foreign gene into the host genome. Transient expression can be achieved by various means, but generally implies the use of a “transfer vector,” bacterial, viral or purely mechanical, which mediates the passage of genetic information in the form of a polynucleotide (in this example, a strand of DNA) into a host cell. Once in the cell, the genetic information is generally used directly as a template for transcription into mRNA molecules which are subsequently translated into a protein by the host cellular machinery. For further information refer to the Investigator Brochure.

6.2.1 Composition

The H5 VLP vaccine is a clear liquid suspension consisting of Hemagglutinin (H5) protein VLP’s (virus strain A/Indonesia/5/05, clade 2.1) of an approximate molecular weight of 72 kDa, in a PBS buffer (100mM PO_4_, 150mM NaCl, 0.01%Tween 80; pH 7-7.6). The H5 protein concentration in the vaccine will be 50µg/ml, 25µg/ml or 12.50µg/ml for final dosages of 20µg, 10µg or 5µg, respectively.

6.2.2 Preparation and Administration

Subjects will receive two 0.5ml intra-muscular doses of H5 VLP (H5N1) pandemic influenza vaccine combined with Alhydrogel® 1%, in the deltoid muscle, preferably in the non-dominant arm, twenty-one (21) days apart. Whenever possible, the injection will be given in the opposite arm from which serum samples are drawn. The H5 VLP influenza vaccine will be contained in a 2ml borosilicate vial (type 1) consisting of a volume of 0.6 ml of vaccine at concentrations of 50µg/mL, 25µg/mL or 12.5µg/mL, for the doses of 20µg, 10µg and 5µg, respectively. For the final dose administered, each of these will be diluted with 0.15mL of 1% Alhydrogel® (provided separately, 1.0mL per vial) to yield a total of 0.75mL of formulated vaccine in each vial. Each subject will be administered 0.5mL, resulting in final dosages of 20µg, 10µg or 5µg, respectively. Rubber stoppers should never be removed from any vial. The remaining 0.25 ml of vaccine in each vial will be discarded. The 0.5mL vaccine dose administered as well as the discarded 0.25mL will be recorded on the study vaccine accountability log.

Study vaccine or placebo will be administered by an un-blinded staff member who will not participate in any other aspects of the study.

The following steps should be taken to prepare study vaccine mixed with 1% Alhydrogel®:

- 1. Clean the surface of the H5 VLP vaccine vial (0.6mL) stopper with an isopropyl alcohol swab and allow the antiseptic to dry completely.
  2. Vigorously shake the Alhydrogel® 1% vial by inverting 5 – 6 times to ensure the suspension is mixed appropriately (uniform distribution of the white gel).
  3. Clean the surface of the vial stopper of the Alhydrogel® 1% with an isopropyl alcohol swab and allow the antiseptic to dry completely.
  4. Draw a volume of air equal to the amount of Alhydrogel® 1% (0.15mL) to be withdrawn from the vial into a disposable 1 mL plastic syringe.
  5. Pierce the vial stopper of Alhydrogel® 1% vial with the sterile needle attached to the syringe.
  6. Turn the vial upside down and inject the air from the syringe.
  7. Immerse the needle into the Alhydrogel® 1% suspension, keeping the needle immersed; withdraw the desired volume of 0.15mL.
  8. Inject immediately the content of the syringe into the study vaccine vial that has been wiped with an isopropyl alcohol swab at step 1 (total volume now 0.6mL + 0.15mL = 0.75mL.
  9. Invert and shake the study vaccine vial 5 to 6 times to ensure the investigational product and Alhydrogel® 1% are combined appropriately.
  10. Each subject will be administered 0.5mL, resulting in final dosages of 20µg, 10µg or 5µg, respectively
  11. The remaining 0.25 ml of vaccine in each vial will be discarded and recorded in the study vaccine accountability log.
  12. Discard the remaining 0.85mL of Alhydrogel® 1% and record in the study vaccine accountability log.
  13. DO NOT VORTEX the vaccine preparations.
  14. **Reconstituted study vaccine and Alhydrogel® 1% MUST be administered to the subject within 5 to 45 minutes.**

The needle on the H5 VLP influenza vaccine combined with Alhydrogel® 1% syringe should be changed prior to administration to the subject. A separate sterile syringe and needle should be used for each injection to prevent transmission of any infectious agents.

**Plant-based H5 VLP (H5N1) pandemic influenza vaccine must not be administered intravenously.**

Prior to vaccine administration to the study subject, clean the skin over the deltoid muscle with a suitable antiseptic (e.g., isopropyl alcohol) and wipe dry with a clean, dry gauze. The needle used for the injection should be of sufficient length to reach the substance of the muscle (at least one inch or 2.5 cm or longer based on the subject’s weight). Following needle insertion into the deltoid muscle, the end of the syringe should be stabilized and the piston of the syringe pulled back to ensure the needle has not entered a blood vessel. If blood is aspirated, the needle should be withdrawn, and a new injection prepared and administered. Documentation of the blood-aspirated dose should be noted on the site vaccine accountability log for monitoring purposes.

The placebo preparation will be a phosphate buffer (100 mM phosphate buffer + 150 mM NaCl + 0.01% Tween 80) combined with Alhydrogel® 1%. The phosphate buffer (labelled placebo) will be contained in a 2 ml borosilicate vial (type 1) consisting of a volume of 0.6 ml of placebo per vial. For placebo administration, 0.6ml vials will be diluted with 0.15ml of 1% Alhydrogel® (provided separately, 1 ml per vial) to yield a total of 0.75ml of formulated placebo in each vial. Each subject will be administered 0.5ml of the placebo. The remaining 0.25 ml of placebo in each vial will be discarded. Administration of the placebo preparation and discarding of remaining placebo will be recorded in the study vaccine accountability log.

Alhydrogel 1% should be stored at room temperature in a secured location. Further specific information relating to storage and shipment of study vaccines and the placebo preparation will be presented in the Study Procedures Manual.

6.2.3 Precautions for Use

Plant-based H5 VLP (H5N1) pandemic influenza vaccine must not be administered intravenously. The test articles should be stored at 2ºC to 8ºC. Do not freeze vaccine as this destroys activity. **Do not use vaccine that has been frozen.**

6.2.4 Dose Selection and Timing

Thirty-six (36) subjects will be randomized to receive two (2) i.m. injections, 21 days apart, of either 5µg or 10µg or 20µg H5 VLP (H5N1) pandemic influenza vaccine combined with Alhydrogel® 1%.

H5 VLP influenza vaccine lot number:

Dose of 5 µg Lot number: PS-130709-111 Expiry date: October 23^rd^ 2009

Dose of 10 µg Lot number: To be confirmed Expiry date: To be confirmed

Dose of 20 µg Lot number: To be confirmed Expiry date: To be confirmed

6.3 Identity of the Control Product

The control product (placebo) will consist of a 100mM phosphate buffer + 150mM NaCl + 0.01% Tween 80 and which will be combined with Alhydrogel® 1% in vials.

Lot Number: E101 and WFI 1:1 Lot Number : E201

Expiry date: December, 2010

6.3.1 Composition

The control product (placebo) will consist of a volume of 0.6mL -100mM phosphate buffer + 150 mM NaCl + 0.01% Tween 80 and will be combined with 0.15mL Alhydrogel® 1%.

6.3.2 Preparation and Administration

Subjects will receive two intra-muscular doses of 0.5mL, 100mM phosphate buffer + 150mM NaCl + 0.01% Tween 80 combined with Alhydrogel 1% (final concentration of 0.4%).

As the volumes and color of the study vaccine and the placebo control and adjuvant, as described above, are the same, the randomization of each subject to treatment or placebo will be maintained. However, the dose-concentration will not be blinded to the clinic staff.

6.3.3 Precautions for use

There are no known precautions for use.

6.4 Identity of Other Products

There will be no other products tested in this clinical trial.

6.5 Product Logistics

6.5.1 Labelling and Packaging

It is the responsibility of the Sponsor to ensure that supplies provided for this study are manufactured under Good Manufacturing Practices (GMP) and are suitable for human use. It is the responsibility of the Sponsor to ship a sufficient amount of dosage units to allow the clinical site to maintain an appropriate supply for the study.

The study vaccine and placebo will be labelled in accordance with all applicable regulatory requirements. All products will be placed in secondary packaging and appropriately labelled.

6.5.2 Storage and Shipment Conditions

Included in the shipment to the clinical site will be the test article, study vaccine H5 VLP (H5N1) pandemic influenza vaccine vials and Alhydrogel® 1%, the control product. A shipment requisition, preparation and expedition form will be included in the shipment, as well as an electronic temperature monitor device known as a “Temptale” and an electronic temperature monitoring device form will also be included. It is requested that the “Temptale” device be stopped upon receipt of the vaccine and the exact date and time is recorded on the “Temptale” form. A pre-addressed envelope for the “Temptale” return to Medicago will be included in the shipment. The vaccines will be immediately placed in the site refrigerator which has been stabilized at a temperature of 2ºC to 8 ºC.

Written authorization will be required from the Project Leader, Sonia Trepanier, prior to administering the study vaccine.

The study vaccines will be stored in a secured, monitored refrigerator and maintained between 2ºC and 8 ºC. A manual log and calibrated thermometer or other temperature recording device will be used to document refrigerator temperature from the time of vaccine receipt and twice (2X) per day during the dosing period. This documentation will be maintained and made available to the Sponsor or designee for review. The site should ensure that appropriate back-up procedures are established for maintenance of the refrigerator temperatures, in the event of a power failure. Such procedures may include an alarm system, back-up electrical system/generator, or a documented contingency plan for maintaining proper refrigeration.

In the event the refrigerator deviates from the 2ºC to 8 ºC required temperature range, the site will do the following:

- As soon as an excursion is discovered, immediately quarantine product involved and contact the Project Leader, Sonia Trepanier at Tel: (418) 658-9393 Ext. 137 or Fax: (418) 658-6699.
- **DO NOT USE PRODUCT** if temperature excursion is outside of the 2ºC to 8 ºC range. Additional vaccine may need to be provided and the site will be advised regarding the immediate return of the compromised product (instruction to be provided by the Sponsor or their designee);
- Report the deviation to the Project Leader, Sonia Trepanier, as well as the Clinical Monitor by preparing a memo that will include the following information:
  - A complete list of the product involved including the protocol number, Investigator name, product name, lot numbers, number of vials, date, the name of the person to contact for additional information, and the signature of the person completing the memo and date.
  - An explanation of the temperature deviation including the date and time the product was received and placed in the refrigerator, the date and time the temperature was last within the normal range, the total amount of time the refrigerator was out of range (hours and minutes) and the out of range temperatures reached.
  - Attach temperature logs for the out of range period to the memo.
  - Forward the memo, along with copies of the appropriate temperature logs, to the Project Leader and Clinical Monitor.

The site should maintain an S.O.P. relating to cold-chain maintenance. The Investigator has overall responsibility for ensuring that study vaccines are stored in a safe, limited access location under the specified appropriate storage conditions. Responsibility may be delegated to a nominated member of the study staff, but this delegation must be documented.

6.5.3 Product Accountability

The study vaccines (test articles) must be used only as directed in the protocol. Vaccines used during the course of the study will be recorded on the Study Vaccine Accountability Log. Any unused vaccine will also be accounted for on the Study Vaccine Accountability Log. The Investigator will be provided with the appropriate forms to ensure accurate, written records are maintained for receipt and disposition of all vaccines received from Medicago Inc.

6.5.4 Replacement Doses

Study vaccine replacement doses will be shipped according to dose level and may be used in the event of breakage or accidental loss while being prepared. All instances must be documented on the Study Vaccine Accountability Log.

6.5.5 Return of Unused Products

The Investigator or designee will sign all study vaccine accountability forms at the end of the vaccination period. Used and unused test article vials must be retained until the study monitor completes reconciliation of test article delivery records with disposition records and accountability of used and unused vials. All test articles must be accounted for and all discrepancies documented accurately. After drug accountability monitoring is completed, the Investigator or designee will return used and unused vials in accordance with instruction provided to:

**Medicago R&D Inc.**

1020, route de l'Église, bur. 600

Sainte-Foy, Quebec

Canada, G1V 3V9

Tel : (418)

Fax : (418)

**Attn: M. Sylvain Rousseau**

6.6 Randomization/Allocation Procedures

The site will be provided a pre-determined sequence of randomization numbers according to the randomization code. Potential study subjects will be screened in a sequential fashion. Once screening is completed and study eligibility is confirmed, the randomization numbers of the pre-determined sequence will be allocated sequentially to subjects within the appropriate treatment group. Once a randomization number has been assigned, it will not be re-used in any event. No subjects will be entered into the study more than once. If a randomization number has been allocated incorrectly, no attempt will be made to remedy the error once study vaccine has been dispensed. The subject will continue with the randomization number and study vaccine. The study staff will notify the Sponsor as soon as the error is discovered without disclosing the study vaccine administered. Admission of subsequent eligible subjects will continue using the next unallocated number in the sequence.

Once it is determined that a study subject is eligible for randomization, a three-digit randomization number, (e.g. 101, 102, 103) and treatment will be assigned. This randomization number and treatment will be recorded along with the two-digit screening number for each subject on the Study Vaccine Accountability Log.

6.7 Blinding and Code Breaking Procedures

The randomization code will not be broken except in emergency cases wherein the identification of the treatment is necessary for appropriate treatment of the subject. A sealed envelope containing the un-blinded randomization schedule may be kept in a locked, secured location at the clinical site. In such circumstances, the un-blinded randomization code may be opened with the permission of the Sponsor Medical Monitor and the treatment assignment would be revealed.

6.8 Concomitant Therapy

Subjects should be instructed not to introduce any new medications without consulting or notifying the Investigator or designee. Any new or changed medications reported by the subject post vaccination and through the Day 42 visit, will be recorded. Since AEs may be secondary to new medications, interviewers will explore the reasons for the new medications and document these AEs. Concomitant medications will include prescription and over-the-counter drugs, vitamins, herbal products and nutritional supplements (any product viewed by the subject as distinct from food). Investigators and other study staff are encouraged to capture the most detailed description of vitamins, herbals, and supplements possible, as otherwise these products may be difficult to classify.

7.0 SPECIMENS AND CLINICAL SUPPLIES

7.1 Management of Samples

Screening blood samples for biochemistry and haematology; urinalysis and urine for pregnancy testing will be performed. Biochemistry, haematology sampling as well as urinalysis will be repeated at Day 21 and Day 42. Serology samples for immunogenicity analysis will also be taken. Refer to the Study Laboratory Manual for complete information on the handling and shipment of all laboratory samples.

7.1.1 Sample Collection

Blood samples will be collected for screening haematology as follows: haemoglobin, hematocrit (packed cell volume, PCV, red blood cells, platelets, mean cell haemoglobin (MCH), mean cell concentration (MCHC), mean cell volume (MCV), white cell count (total, WBC), neutrophils, lymphocytes, monocytes, eosinophils, and basophils. The following biochemistry tests be will conducted: sodium, potassium, urea, creatinine, glucose, alkaline phosphatase, alanine transferase (ALT), serum glutamate pyruvate transaminase (SGPT), bilirubin, albumin, urate, aspartate transferase (AST, SGOT), gamma glutamyl transferase (GGT), chloride, cholesterol and triglyceride. Urinalysis testing will include pH, specific gravity, glucose, protein and blood. A pregnancy urinalysis will also be conducted. Refer to the study Laboratory Manual for further information.

Four (4) samples for serological analysis will be collected from each study subject (~10mL each time or 2X clot-tube or red stopper). HAI antibody titres will be determined based on sera drawn immediately prior to administration of the first dose of study vaccine. Twenty-one (21) days later, prior to administration of the second dose of vaccine, a serology sample will be drawn. Serology samples will also be drawn at the time of the Day 42 visit and the final visit on Day 228. Should a subject be discontinued from the study, a sample will be drawn at the time of the final safety visit. Four (4) samples for cell-mediated assays will also be collected from each study subject (~2mL or 4X 5mL EDTA tube) prior to the first immunization and at Days 21, 42 and 228. These samples will be cryopreserved on the day of collection and held for later testing.

7.1.2 Sample Preparation

Preparation of the laboratory samples for the collection and shipping of biochemistry, haematology and urinalysis will be described in the Study Laboratory Manual.

Ship samples to Laboratory on the day of collection. Label each tube with the label provided and complete the subject ID number, date, visit #.

Blood samples for serology will be collected in one (1)10mL serum separator tube. The samples will be kept at room temperature for a maximum of 30 minutes and stored in the fridge for a maximum 24 hours until centrifuged at the appropriate speed (1200g) for 10 minutes at 4ºC or for five (5) minutes at ambient temperature. Serum will be divided into 4 aliquots into 4 separate polypropylene tubes as soon as possible: two of at least 0.5mL (when possible) and the other two containing equal amounts of the remainder of the serum (when possible).

Medicago R&D Inc. will measure HAI titres in serum samples using a validated method. Serum samples from subjects who do not complete the study through Day 42, due to adverse events, lost to follow-up or insufficient amount of serum will be analyzed and reported, but the results will not be used for the study analysis.

Samples for cell-based assays will be shipped at room temperature to Dr Ward’s laboratory at the MUHC-RI to arrive within 4 hours of collection (Attention Angela Brewer, R3-103, Montreal General Hospital, 1650 Cedar Av, Montreal, QC, H3G 1A4). There, peripheral blood mononuclear cells will be isolated and cryopreserved in 2-4 aliquots (depending upon cell counts) at ~5x10^6^ cells/mL. These cells will be held in liquid N vapour until used.

7.1.3 Sample Storage and Shipment

Serology serum samples should be stored at approximately minus -20º C or lower, until analysis.

The Day 0, Day 21 and Day 42 samples and reserve samples will be sent to Medicago R&D Inc. in eight (8) shipments; Day 0 samples, Day 0 back-up samples, Day 21 samples, Day 21 back-up samples, Day 42, Day 42 back-up samples, Day 228 samples and Day 228 back-up samples. Once the analytical laboratory confirms the receipt of the first shipment, the second set of aliquots of reserve samples may be sent. No samples should be shipped on a Friday or the weekend.

All shipments must be accompanied by the shipping manifest provided by the study Sponsor.

The shipment will be delivered to the following address:

Michèle Dargis

Manager, Analytical Development

Medicago R&D Inc

1020, route de l’Eglise, bureau 600

Sainte-Foy, Québec G1V 3V9

Tel (418) 658-9393 Ext. 150

7.2 Clinical Supplies

Medicago R & D Inc. will supply the clinical study site with all necessary HAI testing supplies (sampling tubes, aliquot tubes, aliquot labels, aliquot storage and shipping boxes and accompanying manifests). Disposable syringes, needles and SST tubes will not be supplied by the study Sponsor. Complete study sample management procedures are described in the Study Laboratory Manual provided by the Sponsor.

8.0 ASSESSMENTS METHODS AND ENDPOINTS

8.1 Safety Endpoints, Immunogenicity Endpoints, and Assessments Methods

8.1.1 Endpoints

8.1.1.1 Primary Endpoints

Safety and tolerability endpoints to be collected include solicited symptoms, oral temperature and local injection site pain, erthyema (redness) and swelling. Systemic complaints reasonably anticipated to occur as a result of receipt of the test articles will be graded as mild, moderate or severe as follows: headache, muscle aches, joint aches, fatigue, chills, feelings of general discomfort, or uneasiness, swelling in the axilla, groin, neck or chest. These signs and symptoms will be measured and reported by each study subject from the time of study vaccine administration to (Day 0 – Day 1 and Day 2 – Day 7) seven days post-vaccination.

- - Percentage, intensity and relationship of immediate complaints for at least 2 hours post-vaccination)
  - Percentage, intensity and relationship to vaccination of solicited local and systemic signs and symptoms 7 days following each dose of study vaccine
  - Percentage, intensity and relationship of solicited and unsolicited local and systemic signs and symptoms 21 days following each dose of study vaccine
  - Occurrence of all adverse events and serious adverse events
  - The number and percentage of subjects with normal and abnormal haematological and biochemical values at Screening, Day 21 and 42
  - The number and percentage of subjects with normal and abnormal urine values at Screening, Day 21 and 42

Any adverse events or serious adverse event will be collected throughout the course of the study. The collection of adverse events will begin following the signature of the Informed Consent Form.

Any clinical laboratory test result that meets the criteria for an AE in the absence of appropriate and/or adequate clinical diagnosis should be reported as an A/E, unless the result is considered normal for the current trial subject population.

8.1.1.2 Secondary Endpoints

Geometric mean titres: (GMTs) of hemagglutination inhibition (HAI) antibody will be evaluated on Day 0, Day 21, Day 42. Follow-up serology samples for GMTs will be taken at Day 228. GMTs will be analyzed as follows:

- Seroconversion factor or GMFR (Geometric Mean Fold Rise): is the geometric mean of the ratio of GMTs (Day 21/Day 0 and Day 42/Day 0).
- Seroconversion rate: the proportion of subjects in a given treatment group with either a ≥ 4-fold increase in reciprocal HAI titres between Day 0 and Days 21 and 42; or a rise of undetectable HAI titre (i.e. < 10) pre-vaccination, (Day 0) to an HAI titer of ≥40 at Day 21 and 42 post-vaccination.
- Seroprotection rate: the proportion of subjects in a given treatment group attaining a reciprocal HAI titer of ≥40 at 21 and 42 days post-vaccination (the percentage of vaccine recipients with a serum HAI titer of at least 1:40 following vaccination).

8.1.1.3 Exploratory Endpoints

The nature of the cell-based immune response will be assessed using two different methodologies:

- Flow cytometry will be used to assess the percentage and activation state of PBMC subsets following re-stimulation with vaccine antigen (and control antigen) in vitro (e.g. CD69 on lymphocytes, HLA class II on monocytes). Flow cytometry will also be used to assess intracellular cytokine production by antigen re-stimulated T cells (e.g. IFNγ production by CD8 cells).
- The profile of cytokines produced by PBMCs from subjects cultured in presence of vaccine antigen (vs. control antigen) will be assessed using multi-plex technology. The cytokines measured will include both Th1 type (IL-2, IL-15, IL-17, IFN-γ, and Th2 type (IL-4, IL-5, IL-6, IL-10, IL-13) as well as inflammatory/chemokine type (IL-1α, IL-1β, IL6, IL-8, TNF-α, RANTES). Antigen-specific lymphoproliferation will also be assessed using aliquots of these PBMC by either BrdU incorporation or CFSE.

8.1.2 Assessment Methods

These data are exploratory only and would not normally be included in any submission documentation. Straightforward statistical analysis (e.g: t-tests or ANOVA) will be used to compare the relative proportions of cells (e.g: CD4/CD8 rations) as well as the proportions of cells expressing particular activation markers or cytokines.

8.1.3 Handling of Missing Data and Outliers

8.2 Efficacy Endpoints and Assessment Methods

The EMEA (CHMP) has issued requirements (Note for Guidance on Harmonisation of Requirements for Influenza Vaccines), 12 March 1997 - CPMP/BWP/214/96, regarding the need to test annual modifications to influenza vaccine strains. The purpose is to verify the tolerance or incidence of adverse reactions and immunogenicity of the hemagglutinin of the vaccine strains, including the titer and frequency of anti-HA antibody responses.

In this clinical trial and in accordance with the most recent EMEA (CHMP) guideline entitled “*Guideline on Influenza Vaccines Prepared from viruses with the potential to cause a pandemic and intended for use outside of the core dossier context*,” February 2007 – EMEA/CHMP/VWP/263499/2006: “all three criteria (seroprotection rate, GMT increase and response rate) as defined below should be fulfilled.”

- Number of seroconversions or significant increase in anti-haemagglutinin antibody titer >40%;
- Mean geometric increase > 2.5;
- The proportion of subjects achieving an HAI titer of ≥40 should be 70%

9.0 SERIOUS ADVERSE EVENTS

9.1 Reporting of Serious Adverse Events

A Serious Adverse Event (SAE) (experience) or reaction is any untoward medical occurrence (whether considered to be related to study vaccines or not) that at any dose:

- Results in Death;
- Is life-threatening (at the time of the event);
- *NOTE: The term “life-threatening in the definition of “serious” refers to an event in which the patient was at risk of death at the time of the event; it does not refer to an event which hypothetically might have caused death if it were more severe.*
- Requires inpatient hospitalization (≥ 24 hours) or prolongation of existing hospitalization (elective hospitalizations/procedures for pre-existing conditions that have not worsened are excluded);
- Results in persistent or significant disability/incapacity; or
- Is a congenital abnormality/birth defect.

Medical and scientific judgment should be exercised in deciding whether expedited reporting is appropriate in other situations, such as important medical events that may not be immediately life-threatening or result in death or hospitalization but may jeopardize the patient or may require intervention to prevent one of the other outcomes listed in the definition above. These should be considered serious. Examples of such events are intensive treatment in an emergency room for allergic bronchospasm, blood dyscrasias or convulsions that do not result in hospitalization, or development of drug dependency or drug abuse.

9.1.1 Adverse Events

An adverse event or adverse experience (AE) is defined as any untoward medical occurrence in a patient or clinical investigation subject administered a pharmaceutical product and which does not necessarily have to have a causal relationship with the treatment. An adverse event can be any favourable and unintended sign (including an abnormal laboratory finding, for example), symptom, or disease temporally associated with the use of a medicinal product, whether or not considered to be a medicinal product.

The following information regarding each AE will be obtained: date and time of onset and resolution (duration), intensity (defined below), whether it was serious, any required treatment or action taken, outcome, relationship to the investigational vaccine, and whether the AE caused withdrawal from the study.

The intensity of all AEs will be graded as mild, moderate, or severe using the following definitions:

**Mild:** The subject is aware of the AE but the event causes no limitation of usual activities.

**Moderate:** The subject is aware of the AE and the event causes some limitation of usual activities.

**Severe:** The AE is of such severity it results in an inability to carry out usual activities.

The Investigator will be instructed to closely monitor each subject who experiences an AE (whether ascribed to the investigational product or not) until the outcome of the AE has been determined.

Any adverse events or serious adverse events occurring following the signature of the informed consent form will be recorded as pre-dose events.

Adverse events and/or solicited local and systemic reactions of greater than (>) grade 2 (moderate) severity will be reported promptly (24-48 hours) to the Investigator, who may request the subject come to the clinic for evaluation at his/her discretion. The Investigator will document review of these AEs on the source document.

If any of the solicited local or systemic reactions noted at the Day 7 telephone contact persist beyond Day 8, these will be considered an AE. The subject will be requested to note when the AEs resolve and report this information to the Investigator or clinic staff at the Day 21 or Day 42 visit.

In the event of a clinically important adverse event, a suitable sample will be collected for drug assay or for additional laboratory tests. The Investigator must ensure that the sample is properly labelled and stored. The Investigator and others responsible for care of the subjects should institute any supplementary investigations of significant adverse events based on the clinical judgment of the likely causative factor. This may include seeking a further opinion from a specialist in the field of the adverse event.

9.1.2 Expectedness of an Adverse Drug Reaction

An “unexpected” adverse reaction is one, the nature or severity of which, is not consistent with information in the relevant source document(s). Until source documents are amended, expedited reporting is required for additional occurrences of the reaction.

9.1.3 Initial SAE Reporting by the Investigator

Serious adverse events related to pre-treatment study procedures will be reported from the time the Informed Consent is signed. All post-vaccination SAEs will be reported from the time of receiving the study vaccine through the Final Safety Visit, Day 228. The study Investigator must report via telephone, all SAEs, whether related to the test article or not, to the Medical Monitor, within 24 hours (one business day) of the Investigator’s learning of the event. The Investigator must also complete, sign and date the SAE report form, and fax a copy to:

Scott Halperin, M.D.,

Telephone: (902) 470-8502 Pager: (902) 458-0492

Mobile: (902) 449-8502

A copy must also be faxed to:

Attn: Sonia Trepanier, Project Leader

Medicago R&D Inc.

1020, route de l’Eglise, bureau 600

Sainte-Foy, Québec G1V 3V9

Tel (418) 658-6699

Fax (418) 658-(418) 658-6699 Mobile: (418) 655-7158

9. 1.4 Follow-up Reporting by the Investigator

Serious adverse events, regardless of causality, will be followed to resolution until the event returns to the baseline condition, the event stabilizes, or if ongoing at Day 42, the SAE will be followed until resolution and until 30 days following the final safety visit on Day 228. Where appropriate, documentation of any medical tests or examinations performed will be provided to document resolution of the event.

9.1.5 Reporting of SAEs Occurring after Subject Trial Termination

All SAEs will be followed until resolution or for a period of 30 days from the final study visit on Day 228.

9.1.6 Causal Relationship

The Investigator must make the determination of relationship to the test article for each adverse event. The Investigator should decide whether, in his/her medical judgement, there is a reasonable possibility that the event may have been caused by the investigational vaccine. If there is any valid reason, even if undetermined or untested, for suspecting a possible cause-and-effect relationship between the investigational vaccine and the occurrence of the adverse event, then the adverse event should be considered “definitely related,” “probably related” or “possibly related”. Otherwise, if no valid reason exists for suggesting a possible relationship, then the adverse event should be classified as “probably not related.” The following guidance may be helpful:

**Probably Not Related**: There is no medical evidence to suggest that the AE is related to the test article. The event can be readily explained by the subject’s underlying medical condition or concomitant therapy or lacks a plausible temporal relationship to the test article.

**Possibly Related**: A direct cause and effect relationship between the test article and the AE has not been demonstrated but there is a reasonable possibility that the event was caused by the test article.

**Probably Related**: There probably is a direct cause and effect relationship between the AE and the test article. A plausible biologic mechanism and temporal relationship exist and there is no, more likely explanation.

**Definitely Related**: There is a direct cause and effect relationship between the AE and the test article.

The medical assessment of intensity will be determined by using the following definitions:

**Mild:** The adverse event is easily tolerated and does not interfere with usual activity.

**Moderate:** The adverse event interferes with daily activity, but the subject is still able to function.

**Severe:** The adverse event is incapacitating and the subject is unable to work or complete usual activity.

The outcome may be classified as resolved, ongoing, resolved with sequelae, or death.

9.1.7 Reporting of SAEs to Health authorities and IEC/IRBs

Medicago R&D Inc. will be responsible for reporting SAEs that are deemed possibly related to test article and unexpected (Unexpected refers to not appearing in the package labelling or in the study vaccine Investigator’s Brochure to regulatory authorities in an expedited manner.

The Investigator will be responsible for reporting all SAEs directly to their relevant ethical review body (IEC/IRB) as soon as possible, and will also provide the ethical review body with any safety reports prepared by the study Sponsor.

9.1.8 Seven (7) - Day Safety Monitoring

A review of 7-day safety data will be carried out to ensure ongoing safety of study subjects as well as maintaining study scientific integrity. This review will detect any early negative trends in the safety data and may necessitate a decision to return to a lower dose level. A telephone call reminding study subjects to complete the Memory Aid will be made at Day 1 post-vaccination. Eight (8) days following each vaccination, subjects will be telephoned by clinic staff to review and record 7-day safety data. Early accumulated safety outcome data which will include all self-reported solicited local and systemic symptoms, any adverse and / or serious adverse events occurring following administration of each injection at the first two dose levels, will be collected and tabulated.

The safety experts will be provided the following blinded 7-day solicited local and systemic symptoms:

- Occurrences of erthyema, swelling and pain at the injection site
- Occurrences of fever, headache, muscle aches, joint aches, fatigue, chills, and feelings of general discomfort or uneasiness
- Swelling in the axilla, groin, neck or chest
- Occurrence of any adverse or serious adverse events
- Oral temperature results from the first seven days post vaccination will also be provided
- Concomitant medications, doctor or emergency room or hospital visits associated with an adverse event or serious adverse event

Safety data for all subjects in the first cohort receiving the 5µg dose or placebo, will be tabulated and reviewed by the Investigator and 2 medical advisors, prior to permitting the second cohort to receive a higher dose (10µg) or placebo, of study vaccine. The same procedure will be repeated with the second cohort of sixteen subjects. Following a favourable review of the second cohort, who received 10µg of study vaccine or placebo, this cohort’s seven-day safety data, will be reviewed. Following a favourable review, cohort 3 subjects will be permitted to be randomized to the highest dose level (20µg). There will be one safety review following the first dose of the 20µg dose level. In total, there will be five (5) seven-day safety reviews following each injection from cohort 1 and cohort 2. Due to the timeframe of the analysis involved, the seven – Day safety data may not be monitored for the purposes of the 7-day safety report, but these data will be monitored in advance of the interim study report.

This safety data may be un-blinded based on the reviewers’ medical judgement.

Stopping rules or conditions for stopping this clinical trial would occur if there was clear evidence of harm or harmful side effects such as Serious Adverse Events related to the treatment (study vaccine). A Serious Adverse Event which was thought to be unrelated to the study vaccine would not warrant stopping the trial.

Based on the medical experts’ opinion, the following criteria may result in the stopping of the clinical trial:

- - - More than one (1) SAE probably related to the study vaccine.
    - Solicited Local injection site symptom reactions >grade 2 (moderate) severity = > 70%
    - Solicited Systemic symptom reactions > grade 2 (moderate) = > 50%

Refer to a sample outline of the safety narrative in Appendix C.

10.0 DATA COLLECTION AND MANAGEMENT

10.1 Data Collection, CRF Completion

None of the data to be collected during this trial will be estimated, if missing. Once the final study subject has completed the study evaluations and the CRF have been entered and queries resolved, the database can be locked following appropriate QC/Quality Assurance (QA) procedures.

The Investigator must submit a completed CRF for each subject who receives the study vaccine. CRFs should be signed and dated by the Investigator to document his review of the data and acknowledgement that the data are accurate. The CRFs should be completed in blue indelible ink and be legible. If an entry on a CRF requires change, the correction should be made as follows:

Draw a single line through the incorrect entry.

Enter the correct data and initial and date the change. Correction fluids, markers, erasures or any form of obliteration on CRFs are not permitted under any circumstances.

All data fields should be completed. Where a page or pages are unused, the subject’s initials and number must still be entered in the appropriate fields, with a single line drawn through the remainder of the page.

The CRFs should be completed in black pen and be legible. CRF completion guidelines will be issued.

10.2 Data Management

Data management will be performed at PharmaNet Specialized Pharmaceutical Services (SPS).

11.0 STATISTICAL METHODS AND DETERMINATION OF SAMPLE SIZE

11.1 Statistical Methods

Data analysis will be performed at Anapharm Inc.

An interim clinical study report analyzing safety and immunogenicity at 42 days post-vaccination will be completed.

All descriptive and inferential statistical analyses will be performed using SAS^®^ Version 8.2 or higher statistical software. Descriptive statistics for continuous variables will consist of the mean, median, geometric means (where appropriate), standard deviation, minimum, and maximum values. For categorical variables, the number and percent of each category will be displayed. Frequency tables for categorical variables may be performed using Microsoft^®^ Office Excel 2003 or higher version.

For the analysis of safety and immunogenicity endpoints, a two-sided alpha level of 5% will be used. All statistical tests will be two-tailed unless otherwise stated. No attempt will be made to control type I error rate inflation attributable to multiple comparisons.

Demography will be compared among treatment groups using Fisher’s exact test for categorical factors and the Kruskal-Wallis test for continuous factors.

The calculations and statistical analyses will be further described in a statistical analysis plan.

11.1.1 Hypotheses and Statistical Methods for Primary Endpoints

11.1.1.1 Hypotheses

To be defined in the statistical analysis plan.

11.1.2 Statistical Analysis

11.1.2.1 Immunogencity

The geometric Mean Titers (GMTs) with 95% confidence intervals (CI) will be determined at Day 0, Day 21 and Day 42 for each treatment group. Summary statistics of the immunogenicity endpoints (seroconversion factor (GMFR), seroconversion rate and seroprotection rate) will be calculated for each treatment group at each of Day 21 and Day 42.

Seroconversion factor (GMFR) will be compared between treatment groups using ANOVA at each of Day 21 and Day 42. Statistical analyses will be performed on log-transformed data. If a statistically significant treatment effect is found, multiple comparisons will be done using Duncan’s multiple range test.

Seroconversion rates and seroprotection rates will be compared (each vaccine dose level versus placebo) using Fisher’s exact tests at each of Day 21 and Day 42.

11.1.2.2 Safety

Safety endpoints will be compared (each vaccine dose level versus placebo) using Fisher’s exact tests.

11.1.3 Hypotheses and Statistical Methods for Exploratory Endpoints

(Refer to Statistical Analysis Plan)

11.2 Population to be Analyzed

11.2.1 Definition of Population

11.2.1.1 Intent-to-Treat Safety Analysis Set

The Safety Set is defined as all subjects who received any study treatment.

11.2.1.2 Full Analysis (Intent-to-Treat) Immunogenicity Population

The Full Analysis Set will consist of the subset of the Safety Set with a Day 0 and any post-vaccine immunogenicity assessment. Subjects who received the wrong treatment will be analyzed as randomized.

11.2.1.3 Per-Protocol Immunogenicity Population

The Per Protocol Set will consist of the subset of the Full Analysis Set who completed the study with no major protocol violation. For the Day 21 analysis, this should include the subjects who received the first vaccine dose and had Day 0 and Day 21 immunogenicity measurements. For the Day 42 analysis this should include the subjects who received both vaccine doses and had Day 0 and Day 42 immunogenicity measurements.

11.2.2 Populations Used in Analysis

Safety analyses will be carried out on the Safety Set. Primary immunogenicity analyses will be performed on the Per Protocol Set. If necessary, the Full Analysis Set could also be used for immunogenicity analyses.

11.3 Determination of Sample Size and Power Calculation

The sample size is limited as this is a first-in-human study. Nevertheless, 12 subjects per treatment groups will allow observing an adverse reaction having 10% incidence with about 72% probability. An adverse reaction having 5% incidence will be observed with about 46% probability.

12.0 ETHICAL AND LEGAL ISSUES AND INVESTIGATOR / SPONSOR RESPONSIBILITIES

12.1 Ethical Conduct of the Trial / Good Clinical Practice

The study will be conducted in accordance with the ethical principles that have their origins in the most recent update of the Declaration of Helsinki, International Conference on Harmonization (ICH) Guideline E6, GCP rules and applicable Canadian regulatory requirements.

The Investigator is responsible for obtaining written approval for the clinical study protocol (including all protocol amendments), the written subject informed consent form, informed consent updates, subject recruitment procedures (e.g. advertisements) and any other written information to be provided to the subject which complies with local regulatory requirements.

The Investigator is responsible for maintaining a copy of the approval document in the study documentation files and for providing a copy to Medicago R&D Inc.

The Investigator should submit written reports of the clinical study status to the IEC/IRB annually or more frequently if requested by the IEC/IRB. A final study notification should be forwarded to the Independent Research Ethics Committee within 90 days after the study has been completed or in the event of premature termination of the study. Copies of all clinical study status reports (including termination) should be provided to Medicago R&D Inc. The end of the trial is defined as the date of the last contact with any participant in the trial. The end of the trial must be notified to the IEC/IRB and Health Canada within 90 days or within 15 days if the trial is halted or terminated early.

Following the reporting of serious adverse reactions and events to Medicago R&D Inc. (Section 9.1.1) the Investigator must also promptly inform the IEC/IRB of these; or other safety related information reported from Medicago R&D Inc.

Modifications to the protocol should be made as an amendment and should be approved/acknowledged both by Medicago R&D Inc. and the IEC/IRB prior to being implemented unless the amendment is made to eliminate an immediate hazard to the clinical study subjects. Medicago R&D Inc. shall be responsible for notifying the appropriate regulatory authorities of any substantial amendments to the protocol.

The Investigator is responsible for notifying the Independent Research Ethics Committee of all protocol amendments.

Administrative amendments (e.g. changes in telephone number, etc.) will not require approval by the Independent Research Ethics Committee unless requested by the IEC/IRB.

12.2 Source Documents and Source Data

Source documents are original documents, data and records of clinical findings, observations and other activities in a clinical study necessary for the reconstruction and evaluation of the study. Source documents will include: all logs and notes from the study site, laboratory records specific to this study and relevant: hospital records, clinical and office charts, laboratory notes, memoranda, evaluation checklists, pharmacy dispensing records, recorded data from automated instruments, copies or transcriptions certified after verification as being accurate copies, microfiches, photographic negatives, microfilm or magnetic media, x-rays, subject files and records kept at the pharmacy, at the laboratories and at medical/technical departments involved in the clinical study.

12.3 Confidentiality of Data and Access to Subject Records

Data collected during this study will be used to support the development, registration and future marketing of H5 VLP (H1N1) pandemic influenza vaccine. All data collected during the study will be controlled by the Sponsor or designee and the Sponsor will abide by all relevant data protection laws. After subjects have consented to take part in the study, their medical records and data collected during the study will be reviewed by representatives of the Sponsor and / or the company organizing the research on the Sponsor’s behalf to confirm that the data collected is accurate and for the purpose of analyzing the results. These records and data may additionally be reviewed by auditors, interested commercial parties or by regulatory authorities. The subject’s name, however, will not be disclosed outside the clinical site but rather by a unique subject number. The results of this study may be used in submissions to other countries throughout the world, which have ensured an adequate level of protection for personal data.

To ensure compliance with relevant regulations, data generated by this study must be available for inspection upon request by the Sponsor or an authorized representative of Medicago R&D Inc., regulatory authorities and the IRB/IEC.

12.4 Monitoring, Auditing and Archiving

12.4.1 Monitoring

The study will be monitored by representatives of Medicago R&D Inc. throughout its duration by means of personal visits to the Investigator’s facilities and other communications. Monitoring visits by Medicago R&D Inc should be scheduled at mutually agreeable times periodically throughout the study. These visits will be conducted to evaluate the progress of the study, verify the rights and well-being of the subjects are protected, verify the reported clinical study data are accurate, complete and verifiable from source documents, and the conduct of the study is in compliance with the approved protocol and amendments, GCP and applicable regulatory requirements. A monitoring visit should include a review of the essential clinical study documents (regulatory documents, CRFs, source documents, drug disposition records, subject informed consent forms, etc.) as well as discussion on the conduct of the study with the Investigator and staff.

The Study Monitor should conduct these visits as frequently as appropriate for the clinical study. The Investigator and staff should be available during these visits to facilitate the review of the clinical study records and resolve/document any discrepancies found during the visit.

12.4.2 Audits and Inspections

In accordance with the principles of GCP, the study may be inspected by regulatory authorities, the Sponsor or assigned designee. The Sponsor is entitled to access information about the status of the study and to review the original study documents or site documents supporting the study.

During the course of the study, Quality Assurance auditors may audit the investigational site. As Medicago R&D Inc representatives, they are entitled to access all subject and study information, excluding the randomized treatment assignments.

12.4.3 Archiving

The Investigator will retain the source documentation including: the original informed consent forms, study documentation (e.g. IRB/IEC approval letters, etc.) and a copy of the completed CRFs for 25 years as per Canadian Food and Drug Regulations; Part C, Division 5, Section C.05.012 (3) (h). After 25 years, the Investigator must contact Medicago R&D Inc. prior to the disposal of any study documents (Adopted from ICH guidelines, GCP, Section 4.9.5). If before the 25 year retention period is completed, the Investigator or institution can no longer maintain the study documentation, Medicago R&D Inc. should be contacted to make other arrangements for storage.

12.5 Financial Contract and Insurance Coverage

Medicago R&D Inc. has suitable insurance to cover clinical trials and product liability. The Investigator site has suitable insurance to cover negligence and default liability.

12.6 Stipends for Participation

Subject stipends for clinical trial participation must be clearly outlined in the Informed Consent Form and approved by the IEC/IRB.

13.0 REFERENCES

^1^ Hilleman, Maurice M., (2002) ‘Realities and enigmas of human viral influenza; pathogenesis, epidemiology and control’, *Vaccine*, 20, Issues 25-26, pp. 3068-3087.

^2^ Clark, Tristan, Stephenson, Iain, Stephenson (2009) “Influenza A/H1N1 in 2009: a pandemic in evolution,” *Expert Reviews Vaccines* 8 (7), pp. 819-822.

^3^ Gϋrtler, Lutz. Virology of Human Influenza. (2006) In “Influenza Report 2006,” Kamps, S.B., Hoffman, C., Preiser, W., eds, Flying Publisher, Paris, pp. 87-91.

**^4^** Canadian Immunization Guide, Seventh Edition (2006) Public Health Agency of Canada, [www.phac-aspc.gc.ca/publicat/cig-gci/index-eng.php](http://www.phac-aspc.gc.ca/publicat/cig-gci/index-eng.php)

^5^ Wright, P. & Webster, R. (2001). Orthomyxoviruses. In *Fields Virology*, 4th edn, Knipe, D., Howley, P. eds, Lippincott Williams & Wilkins, Philadelphia, pp. 1533–1579.

^6^ World Health Organization, Avian Influenza Facts Sheet, [www.who.int/mediacentre/facts](http://www.who.int/mediacentre/facts), February 2006.

^7^ Barry, John M., White (2009) Paper on Novel H1N1 prepared for the MIT Center for Engineering Systems Fundamentals, Working Paper Series, ESD-WP-2009-07.

^8^ [www:nature.com/news/2009/090512/full/459144a.html](http://www.nature.com/news/2009/090512/full/459144a.html)

^9^ Jackson, S., Van Hoeven, N., Chen, L.M., Maines, T., Cox, N., Katz, J., Donis, R., (2009) Reassortment between avian H5N1 and human H3N2 influenza viruses in ferrets: a public health risk assessment, *Journal of Virology,* online 6/05/09.

^10^ Ehrlich, Hartmut J. et al,(2008), A Clinical Trial of a Whole-Virus H5N1 Vaccine Derived from Cell Culture, (2008) *New England Journal of Medicine,* 358, No. 24, June 12, 2008, pp.2573-2584.

^11^ Thompson, W.W. et al, (2003) Mortality Associated with Influenza and Respiratory Synctial Virus in the United States, *Journal of the American Medical Association*, (2) 289, p. 179-186.

^12^ Hampson, A.W., MacKenzie, J.S., (2006) “The Influenza Viruses,” *Medical Journal of Australia*, Nov. 20; 185 (10 Suppl): 539-43.

^13^ Munoz, Flor M., et al (2000) “Current Research on Influenza and other Respiratory Viruses, II International Symposium” *Antiviral Research* 46, Issue 2, pp 91-124.

**^14^** The Canadian Pandemic Influenza Plan for the Health Sector**,** [www.phac-aspc.gc.ca/publicat/cig-gci/index-eng.php](http://www.phac-aspc.gc.ca/publicat/cig-gci/index-eng.php)

^15^ Herring, Ann D., Sattenspiel, (2004) Death in Winter: Spanish Flu in the Canadian Sub-artic In ‘”The Spanish Influenza Pandemic 1918-19 – New Perspectives” Phillips, Howard, Killingray, David, eds, Routledge-Oxford University Press, New York, pp. 156-172.

^16^ U.S. Department of Health and Human Services, Pandemic and Pandemic Threats since 1900, http://www.pandemicflu.gov/general/historicaloverview

^17^ Nicholson, Karl G. et al, (2001) “Safety and antigenicity of non-adjuvanted and MF 59-adjuvanted influenza A/Duck/Singapore/97 (H5N3) vaccine: a randomized trial of two potential vaccines against H5N1 influenza.” *The Lancet* – Early Report Vol. 357, Issue 9272, June 16, pp. 1937-1943.

^18^ Snacken, René et al, (1999) “The Next Influenza Pandemic: Lessons from Hong Kong, 1997, *Emerging Infectious Diseases,*  Vol. 5, No. 2, March-April, www.cdc.gov/ncidod/eid/vol5no2/snacken.htm

^19^ International Federation of Pharmaceutical Manufacturers Association (IFPMA) ifpma.org/Influenza/content/pdfs/Publications/2009_05_Strategies_for_global_access_to_pandemic_influenza_vaccines.pdf

**APPENDIX A – SAMPLE MEMORY AID – Day 0 – Day 7 (Page 1 of 4)**

| **Protocol: CP-H5VLP-001 Memory Aid (MA)** | | **Subject ID No. and Subject Initials:** | **PM, Day 1 Of Dose** | **1 Day after** | **2 Days after** | **3 Days after** | **4 Days after** | **5 Days after** | **6 Days after** | **7 Days after** |
| --- | --- | --- | --- | --- | --- | --- | --- | --- | --- | --- |
| **DAILY TEMPERATURE** | | **Record your oral temperature here (degrees Celsius) ►** |  |  |  |  |  |  |  |  |
| **Fill in today’s column by entering the WORST grade for each symptom that you have had in the last day (24 hours).** | | | | | | | | | | |
| **SYMPTOMS** | | **If you did not have the symptoms, enter “0”** | **PM, Day 1**  **Of Dose** | **1 Day after** | **2 Days after** | **3 Days after** | **4 Days after** | **5 Days after** | **6 Days after** | **7 Days after** |
| ► | Redness where the injection was given | 0 = None 1= More than 0 but NOT more than 20 mm 2= More than 20 mm but NOT more than 50 mm 3= More than 50 mm |  |  |  |  |  |  |  |  |
| ► | Swelling where the injection was given |  |  |  |  |  |  |  |  |  |
| ► | Pain where the injection was given | 0 = None 1= More than 0 but NOT more than 20 mm 2= More than 20 mm but NOT more than 50 mm 3= More than 50 mm |  |  |  |  |  |  |  |  |

|  | **APPENDIX A - Sample Memory Aid – Day 0 – Day 1 (page 2 of 4)** | | | | | | | | | |
| --- | --- | --- | --- | --- | --- | --- | --- | --- | --- | --- |
| ► | Headache | 0 = None  1 = Mild - Noticeable, but doesn’t interfere with my work / normal activities.  2 = Moderate - Bad enough to limit my work / normal activities.  3 = Severe - Bad enough to prevent my work / normal activities |  |  |  |  |  |  |  |  |
| ► | Muscle aches |  |  |  |  |  |  |  |  |  |
| ► | Joint aches |  |  |  |  |  |  |  |  |  |
| ► | Fatigue |  |  |  |  |  |  |  |  |  |
| ► | Chills |  |  |  |  |  |  |  |  |  |
| ► | Feelings of general discomfort or uneasiness |  |  |  |  |  |  |  |  |  |
| ► | Swelling in the axilla (*R/L) |  |  |  |  |  |  |  |  |  |
| ► | Swelling in the groin (R/L) |  |  |  |  |  |  |  |  |  |
| ► | Swelling in the neck |  |  |  |  |  |  |  |  |  |
| ► | Swelling in the chest |  |  |  |  |  |  |  |  |  |
| **Please report any other problem not listed above to the Study Investigator.**  **Symptoms listed above which persist after the time of this 7- day telephone contact should be considered as an adverse event.** | | | | | | | | | | |

*R = Right

*L = Left

**APPENDIX – A SAMPLE MEMORY AID – Day 0 – Day 7 (Page 3 of 4)**

Use the space below to record important information that you want to tell us about changes in your health. Feel free to make a note of any health problems you experience, worsening of old problems, or new medicines you take.

- It is especially important that you make a note of problems that require an unplanned visit to your doctor, a hospital stay or a trip to the emergency room.
- It is also important that you make a note of changes in your medications. Each medication must have a problem associated with it but each problem does not require a medication associated with it.

| **Problem/Severity**  Mild = Noticeable, but doesn’t interfere with my work / normal activities.  Moderate = Bad enough to limit my work / normal activities.  Severe = Bad enough to prevent my work / normal activities | **Date it started**  **(dd/mm/**  **Yyyy)** | **Do you still have it?** | **If it is gone, when did it end?** | **Did you make an unplanned visit or call to a doctor about it?** | **Did you go to an emergency room or hospital? Did you stay in hospital?** | **Put an “X” in this box once you have told us about this problem** |
| --- | --- | --- | --- | --- | --- | --- |
| 1. |  |  |  |  |  |  |
| 2. |  |  |  |  |  |  |
| 3. |  |  |  |  |  |  |
| 4. |  |  |  |  |  |  |

| **DAY 0 – DAY 7 - SAMPLE MEMORY AID – (Page 4 of 4)**  **CHANGES IN YOUR MEDICATIONS** | | | | |
| --- | --- | --- | --- | --- |
| **NAME OF MEDICINE** | **DATE MEDICINE WAS STOPPED/STARTED/CHANGED** | | **WHY WAS MEDICINE STOPPED/STARTED/CHANGED?** | Number above (1,2,3 of the associated problem) |
| 1. |  |  |  |  |
| 2. |  |  |  |  |

**APPENDIX – A SAMPLE MEMORY AID – PROTOCOL - CP-H5VLP-001 – Day 8 – 21 and Day 29 – 42 (Page 1 of 4)**

Use the space below to record important information that you want to tell us about changes in your health. Feel free to make a note of any health problems you experience, worsening of old problems, or new medicines you take.

- It is especially important that you make a note of problems that require an unplanned visit to your doctor, a hospital stay or a trip to the emergency room.
- It is also important that you make a note of changes in your medications. Each medication must have a problem associated with it but each problem does not require a medication associated with it.

| **Problem/ Severity**  Mild = Noticeable, but doesn’t interfere with my work / normal activities.  Moderate = Bad enough to limit my work / normal activities.  Severe = Bad enough to prevent my work / normal | **Date it started**  **(dd/mm/yyyy)** | | | **Do you still have it? (Yes or No)** | **If it is gone, when did it end? (dd/mm/yyyy)** | **Did you make an unplanned visit or call to a doctor about it?** | | **Did you go to an emergency room or hospital? Did you stay in hospital?** | **Put an “X” in this box once you have told us about this problem** |
| --- | --- | --- | --- | --- | --- | --- | --- | --- | --- |
| 1. |  | | |  |  |  | |  |  |
| 2. |  | | |  |  |  | |  |  |
| 3. |  | | |  |  |  | |  |  |
| 4. |  | | |  |  |  | |  |  |
| **CHANGES IN YOUR MEDICATIONS** | | | | | | | | | |
| **NAME OF MEDICINE** | | **DATE MEDICINE WAS STOPPED/STARTED/CHANGED** | | | | | **WHY WAS MEDICINE STOPPED/STARTED/CHANGED** | | |
| 1. | | |  | | | |  | | |
| 2. | | |  | | | |  | | |

**APPENDIX B – SAMPLE TELEPHONE FOLLOW-UP SCRIPT – DAY 0 – DAY 7 (Page 1 of 3)**

| **Protocol: CP-H5VLP-001 Telephone Follow-up Script** | | **Subject ID No. and Subject Initials:** | **PM, Day 1**  **Of Dose** | **1 Day after** | **2 Days after** | **3 Days after** | **4 Days after** | **5 Days after** | **6 Days after** | **7 Days after** |
| --- | --- | --- | --- | --- | --- | --- | --- | --- | --- | --- |
| **DAILY TEMPERATURE** | | **What is your oral temperature? Record (degrees Celsius) ►** |  |  |  |  |  |  |  |  |
| **Remind the subject to use their Memory Aid as a tool to answer the following questions** | | | | | | | | | | |
| **SYMPTOMS** | | **GRADING (Enter “0” if no symptom occurred)** | **PM, Day of Dose** | **1 Day after** | **2 Days after** | **3 Days after** | **4 Days after** | **5 Days after** | **6 Days after** | **7 Days after** |
| ► | Redness where the injection was given | 0 = None 1= More than 0 but NOT more than 20 mm 2= More than 20 mm but NOT more than 50 mm 3= More than 50 mm |  |  |  |  |  |  |  |  |
| ► | Swelling where the injection was given |  |  |  |  |  |  |  |  |  |
| ► | Pain where the injection was given | 0 = None 1= More than 0 but NOT more than 20 mm 2= More than 20 mm but NOT more than 50 mm 3= More than 50 mm |  |  |  |  |  |  |  |  |

**APPENDIX B – DAY 0 – DAY 1 TELEPHONE SCRIPT continued (Page 2 of 3)**

| **Symptom** | **Grading (Enter “0” if symptom did not occur** | **PM, Day of**  **Dose** | **1 Day after** | **2 Days after** | **3 Days after** | **4 Days after** | **5 Days after** | **6 Days after** | **7 Days after** |
| --- | --- | --- | --- | --- | --- | --- | --- | --- | --- |
| **Remind the subject to use their Memory Aid as a tool to answer the following questions** | | | | | | | | | |
| Headache | 0 = None  Mild = Noticeable, but doesn’t interfere with my work / normal activities.  Moderate = Bad enough to limit my work / normal activities.  Severe 3 = Bad enough to prevent my work / normal activities |  |  |  |  |  |  |  |  |
| Joint Aches |  |  |  |  |  |  |  |  |  |
| Fatigue |  |  |  |  |  |  |  |  |  |
| Muscle Aches |  |  |  |  |  |  |  |  |  |
| Feelings of general discomfort or uneasiness |  |  |  |  |  |  |  |  |  |
| Chills |  |  |  |  |  |  |  |  |  |
| Swelling in the axilla (R. or L.)  Swelling in the groin (R. or L.) |  |  |  |  |  |  |  |  |  |
| Swelling in the neck |  |  |  |  |  |  |  |  |  |
| Swelling in the chest |  |  |  |  |  |  |  |  |  |

**APPENDIX B – DAY 0 – DAY 7 TELEPHONE SCRIPT continued (Page 3 of 3)**

| **Question 1.** Has the subject had problems or questions about completing the Memory Aid? | **Yes/ No (Check one)** | If Yes, check here if further training was provided: |
| --- | --- | --- |
| 2. Has any previously unreported change occurred in the health of the subject since the vaccination? | Yes  No | If yes, please describe: |
| 3. Has the subject had any **NEW** health problem since the last contact that resulted in a telephone call to a physician’s office? | Yes  No | If yes, please describe: |
| 4. Has the subject had any **NEW** health problem that resulted in a visit to a physician? | Yes  No | If yes, please describe: |
| 5. Has the subject had any **NEW** health problem since the last contact that resulted in a visit to the Emergency Room (ER)? | Yes  No | If yes, please describe: |
| 6. Has the subject had any **NEW** health problem since the last contact that resulted in a hospitalization? (SAE) | Yes  No | If yes, please describe: |
| 7. Has the subject had any **NEW** health problem since the last contact that resulted in any new medications? | Yes  No | If yes, please describe: |
| **1. Questions 3. - 7: If the answer is YES, the event must be described in the SOURCE DOCUMENTATION and in the appropriate CRF pages. If the event meets SAE criteria, please report as per protocol.** | | |
|  | | |
| 1. **Remind the subject their next visit will be on __________________ at _______________________ (Day and Time)** 2. **Day 1 Call Completed by: ______________________ Da/Mo/YY _______________________** 3. **Day 8 Call Completed by: ______________________ Da/Mo/YY _______________________** | | |

**APPENDIX C – SEVEN (7) DAY SAFETY SUMMARY (SAMPLE)**

1. Narrative / Trial Summary
   1. Summary of Main Findings
   2. Discussion of Issues or Problems
2. Study Description
3. Brief statement of the Purpose of trial
4. Recruitment Status
   1. Enrollment status – (# screened, # enrolled, # vaccinated, # withdrawn or dropped out)
   2. Demographics (age, gender, height, weight, race, ethnicity)
5. Safety Assessments
   1. Percentage, intensity and relationship of solicited local symptoms, Mild/Moderate/Severe (injection site redness, swelling and pain):

- 2 hours post-vaccination
- Day 0 – Day 1 (~24 hours post-vaccination)
- Day 2 - 7 post-vaccination

1. Percentage, intensity and relationship of solicited systemic symptoms (fever, headache, fatigue, chills, muscle aches, joint aches, feelings of uneasiness and general discomfort, swelling of the groin, right or left, axilla, right or left, neck or chest occurring:

- 2 hours post-vaccination
- Day 0 – Day 1 (~24 hours post-vaccination)
- 7 days post-vaccination

1. Percentage, intensity and relationship of any adverse events or serious adverse events occurring during the first 7 days post-vaccination
   1. Based on the medical experts’ opinion, the following criteria may result in the stopping of the clinical trial at any dose:

- More than one (1) SAE probably related to the study vaccine.
- Solicited Local injection site symptom reactions (greater than (>) grade 2 (moderate) severity = > 70%
- Solicited Systemic symptom reactions (greater than (>) grade 2 (moderate) = > 50%

**Table 1 STUDY CP-H5VLP-001 – Enrolment Status**

| Dose Level (1µg or 5µg) | Dose 1 or Dose 2 | No. of subjects screened | No. of screen failures | No. of subjects enrolled | No. of subjects completing Day 7 | No. of subjects withdrawn or drop-out |
| --- | --- | --- | --- | --- | --- | --- |
|  |  |  |  |  |  |  |
|  |  |  |  |  |  |  |
|  |  |  |  |  |  |  |
|  |  |  |  |  |  |  |

**Table 2 STUDY CP-H5VLP-001 - Summary of Demographics and Baseline Characteristics**

Dose 1 or Dose 2 - Dose Level (1µg or 5µg)

| Parameter | n (%) |
| --- | --- |
| Age (years) – Mean (SD) |  |
| Gender = n (%) |  |
| Male |  |
| Female |  |
| Height (cm) – Mean (SD) |  |
| Weight (kg) – Mean (SD) |  |
| Race = n (%) |  |
| Caucasian |  |
| Black or African American |  |
| Asian |  |
| Ethnicity = n (%) |  |
| Hispanic or Latino |  |
| Not Hispanic or Latino |  |
| Received prior influenza immunization 24 months previously |  |
| Had Adverse reaction to prior influenza vaccine |  |

**Table 3 STUDY CP-H5VLP-001 Number and percentage incidence of solicited systemic reactions (Mild, Moderate, Severe) by symptom and day – Dose 1 or 2. Dose Level (1µg or 5µg)**

| Symptom | 2-6 hours post vaccination  n (%) | Day 0  Evening  n (%) | Day 1  n (%) | Day 2  n (%) | Day 3  n (%) | Day 4  n (%) | Day 5  n (%) | Day 6  n (%) | Day 7  n (%) | Total  n (%) |
| --- | --- | --- | --- | --- | --- | --- | --- | --- | --- | --- |
| Fever (Mild, Moderate or Severe) |  |  |  |  |  |  |  |  |  |  |
| Muscle Aches |  |  |  |  |  |  |  |  |  |  |
| Joint Aches |  |  |  |  |  |  |  |  |  |  |
| Fatigue |  |  |  |  |  |  |  |  |  |  |
| Chills |  |  |  |  |  |  |  |  |  |  |
| Malaise (uneasiness and discomfort) |  |  |  |  |  |  |  |  |  |  |
| Swelling of the neck |  |  |  |  |  |  |  |  |  |  |
| Swelling of the groin (R./L) |  |  |  |  |  |  |  |  |  |  |
| Swelling of the axilla (R/L) |  |  |  |  |  |  |  |  |  |  |
| Swelling of the chest |  |  |  |  |  |  |  |  |  |  |

**Table 4. STUDY CP-H5VLP-001 Number and incidence of mild/moderate/severe solicited local reactions by symptom and day – Dose 1**

**or 2. Dose Level (1µg or 5µg)**

| Symptom | 2 to 6 hours post vaccination | Day 0  evening | Day 1 | Day 2 | Day 3 | Day 4 | Day 5 | Day 6 | Day 7 | Total |
| --- | --- | --- | --- | --- | --- | --- | --- | --- | --- | --- |
| Redness (at injection site) Mild/Moderate/Severe |  |  |  |  |  |  |  |  |  |  |
| Swelling (at injection site) Mild/Moderate/Severe |  |  |  |  |  |  |  |  |  |  |
| Pain (at injection site) Mild/Moderate/Severe |  |  |  |  |  |  |  |  |  |  |

**Table 5 STUDY CP-H5VLP-001 Number and percentage incidence of Adverse Event and / or Serious Adverse Events – Dose 1 or 2. Dose Level (5µg, 10µg, 20µg)**

| AE diagnosis (if possible) | 2 hours post vaccination  n (%) | Day 0  Evening  n (%) | Day 1  n (%) | Day 2  n (%) | Day 3  n (%) | Day 4  n (%) | Day 5  n (%) | Day 6  n (%) | Day 7  n (%) | Total  n (%) |
| --- | --- | --- | --- | --- | --- | --- | --- | --- | --- | --- |
| 1. |  |  |  |  |  |  |  |  |  |  |
| 2. |  |  |  |  |  |  |  |  |  |  |
| 3. |  |  |  |  |  |  |  |  |  |  |
| 4. |  |  |  |  |  |  |  |  |  |  |
| 5. |  |  |  |  |  |  |  |  |  |  |
| 6. |  |  |  |  |  |  |  |  |  |  |
| 7. |  |  |  |  |  |  |  |  |  |  |

**Table 6 STUDY CP-H5VLP-001 Number of subjects visiting a health care facility and / or requiring new medications**

**– Day 0 – Day 7**

| Subject ID number | Reason | New Concomitant Medications | Doctor’s visit | Emergency Room Visit | Hospitalization |
| --- | --- | --- | --- | --- | --- |
|  |  |  |  |  |  |
|  |  |  |  |  |  |
|  |  |  |  |  |  |
|  |  |  |  |  |  |
|  |  |  |  |  |  |

1. HA content measured by the BCA method [↑](#footnote-ref-2)
2. HA content measured by the BCA method [↑](#footnote-ref-3)
3. This study compared two different types of VLP. The H5 VLP vaccine was used as the control antigen. [↑](#footnote-ref-4)
4. HA content measured by the BCA method [↑](#footnote-ref-5)
5. HA content measured by the BCA method [↑](#footnote-ref-6)
6. HA content measured by the SRID method [↑](#footnote-ref-7)
7. 7Galli G., Medini D., Borgoni E. *et al*, Adjuvanted H5N1 vaccine induces early CD4+ T cell response that predicts long-term persistence of protective antibody levels, PNAS Early Edition, 10.1073/pnas.0813390106 [↑](#footnote-ref-8)
